# Supplementary material for: Physical frailty, genetic risk, mediating biomarkers, and risk of suicide attempt: A prospective cohort study
Source: PLoS Med. 2026 Apr 6;23(4):e1005045. doi: 10.1371/journal.pmed.1005045 (PMC13065332; doi:10.1371/journal.pmed.1005045)
Supplement: S2 Checklist — This checklist is copyrighted by the Equator Network under the Creative Commons Attribution 3.0 Unported (CC BY 3.0) license.1Skrivankova VW, Richmond RC, Woolf BAR, Yarmolinsky J, Davies NM, Swanson SA, et al. Strengthening the Reporting of Observational Studies in Epidemiology using Mendelian Randomization (STROBE-MR) Statement. JAMA. 2021; under review. 2. Skrivankova VW, Richmond RC, Woolf BAR, Davies NM, Swanson SA, VanderWeele TJ, et al. Strengthening the Reporting of Observational Studies in Epidemiology using Mendelian Randomisation (STROBE-MR): Explanation and Elaboration. BMJ. 2021;375:n2233. (DOCX) [file pmed.1005045.s002.docx]

**STROBE-MR checklist of recommended items to address in reports of Mendelian randomization studies**^1^ ^2^

| **Item No.** | **Section** | **Checklist item** | **Page No.** | **Relevant text from manuscript** |
| --- | --- | --- | --- | --- |
| 1 | **TITLE and ABSTRACT** | Indicate Mendelian randomization (MR) as the study’s design in the title and/or the abstract if that is a main purpose of the study | 1 and 2 | Mendelian randomization (MR) analyses were utilized to examine the association between genetically determined physical frailty and SA. |
|  | **INTRODUCTION** |  |  |  |
| 2 | **Background** | Explain the scientific background and rationale for the reported study. What is the exposure? Is a potential causal relationship between exposure and outcome plausible? Justify why MR is a helpful method to address the study question | 5 and 6 | Exposure: Physical frailty, representing a modifiable aging process, is characterized by reduced multisystem physiological reserve and heightened vulnerability to stressors, leading to impaired homeostasis and increased risk of adverse outcomes such as disability, hospitalization, and mortality.  Plausibility of causal relationship: Frailty has been increasingly recognized as relevant to mental health outcomes. Conceptually, frailty may heighten susceptibility to suicide-related outcomes through multiple interrelated pathways, including functional impairment, loss of independence, chronic inflammation, endocrine dysregulation, metabolic disturbances, and a reduced capacity to cope with psychological and physical stressors.  Why MR is a helpful method to address the study question: These observations support a biologically and clinically plausible hypothesis that FP-measured physical frailty may be associated with increased risk of SA. Hence, a comprehensive study integrating a prospective cohort design with Mendelian randomization (MR) is warranted to better characterize this association.” “Further, we employed two-sample MR analyses as a complementary approach to explore whether the observed association shows genetic support consistent with the cohort findings. Compared with conventional observational analyses, this approach is less susceptible to confounding and reverse causation. |
| 3 | **Objectives** | State specific objectives clearly, including pre-specified causal hypotheses (if any). State that MR is a method that, under specific assumptions, intends to estimate causal effects | 6 and 11 | Against this background, the present study aimed to examine the association between FP-measured physical frailty and risk of SA in a large prospective cohort from the UK Biobank. Further, we employed two-sample MR analyses as a complementary approach to explore whether the observed association shows genetic support consistent with the cohort findings. Two-sample MR analyses were conducted as a secondary and supportive analysis to provide genetic evidence. Compared with conventional observational analyses, this approach is less susceptible to confounding and reverse causation [37]. |
|  | **METHODS** |  |  |  |
| 4 | **Study design and data sources** | Present key elements of the study design early in the article. Consider including a table listing sources of data for all phases of the study. For each data source contributing to the analysis, describe the following: | S1 Methods | UK Biobank-based summary statistics for physical frailty were obtained from a recent study based on 386,565 participants of European descent [5]. We extracted a total of 30 highly associated SNPs (P < 5 × 10−8) that were clumped for independence at r2 < 0.001 with a window of 10,000 kb based on European ancestry reference data from the 1000 Genomes Project. These SNPs were used as instrument variables (IVs). For the outcome of SA, IVs were obtained from the Psychiatric Genomics Consortium (PGC) GWAS. GWAS summary data for SA can be applied via the PGC SUI Data Access Portal (https://pgc.unc.edu/for-researchers/data-access-committee/data-access-portal/). The GWAS summary statistics for SA were consistent with those used to construct the polygenic risk score (PRS). The original GWAS included a total of 15 cohorts and represents the largest genome-wide association study of SA to date [6]. To avoid potential sample overlap, we utilized revised summary statistics that excluded the UK Biobank cohort, resulting in a meta-analysis based on the remaining 14 cohorts, comprising 33,353 SA cases and 444,626 controls. Exposure and outcome data were then harmonized to ensure alignment of the effect alleles. This P-value threshold was selected to ensure that each phenotype included at least three independent IVs, thereby meeting the minimum requirements for MR methods such as MR-Egger regression. For causal effect estimation, we primarily applied the inverse-variance weighted (IVW) method [7], which combines ratio estimates of individual variants using the inverse of their variance as weights. Causal effect estimates are reported as odds ratios (ORs) with corresponding 95% confidence intervals (CIs). As this was a two-sample MR analysis based on GWAS summary statistics, covariate adjustment was performed within the original GWAS analyses rather than in the MR models themselves. To assess the robustness of the findings, we performed several sensitivity analyses. First, Cochran’s Q test was used to evaluate heterogeneity among the IVs [8, 9]. If significant heterogeneity was detected (P < 0.05), we applied a multiplicative random-effects model for validation. Second, the MR-Egger intercept test was used to detect horizontal pleiotropy [10, 11]. When pleiotropy was present (P < 0.05), we employed the MR-PRESSO method to identify and exclude potentially pleiotropic SNPs, and then recalculated pleiotropy-corrected causal estimates. Additionally, we conducted supplementary analyses using the weighted median and MR-Egger methods to further validate the results [10, 11]. Leave-one-out (LOO) analyses identified whether any single SNP drove the causal estimates [8]. |
|  | a) | Setting: Describe the study design and the underlying population, if possible. Describe the setting, locations, and relevant dates, including periods of recruitment, exposure, follow-up, and data collection, when available. |  | Does not apply |
|  | b) | Participants: Give the eligibility criteria, and the sources and methods of selection of participants. Report the sample size, and whether any power or sample size calculations were carried out prior to the main analysis | S1 Methods | UK Biobank-based summary statistics for physical frailty were obtained from a recent study based on 386,565 participants of European descent [5]. We extracted a total of 30 highly associated SNPs (P < 5 × 10−8) that were clumped for independence at r2 < 0.001 with a window of 10,000 kb based on European ancestry reference data from the 1000 Genomes Project. These SNPs were used as instrument variables (IVs). For the outcome of SA, IVs were obtained from the Psychiatric Genomics Consortium (PGC) GWAS. GWAS summary data for SA can be applied via the PGC SUI Data Access Portal (https://pgc.unc.edu/for-researchers/data-access-committee/data-access-portal/). The GWAS summary statistics for SA were consistent with those used to construct the polygenic risk score (PRS). The original GWAS included a total of 15 cohorts and represents the largest genome-wide association study of SA to date [6]. To avoid potential sample overlap, we utilized revised summary statistics that excluded the UK Biobank cohort, resulting in a meta-analysis based on the remaining 14 cohorts, comprising 33,353 SA cases and 444,626 controls. Exposure and outcome data were then harmonized to ensure alignment of the effect alleles. This P-value threshold was selected to ensure that each phenotype included at least three independent IVs, thereby meeting the minimum requirements for MR methods such as MR-Egger regression. For causal effect estimation, we primarily applied the inverse-variance weighted (IVW) method [7], which combines ratio estimates of individual variants using the inverse of their variance as weights. Causal effect estimates are reported as odds ratios (ORs) with corresponding 95% confidence intervals (CIs). As this was a two-sample MR analysis based on GWAS summary statistics, covariate adjustment was performed within the original GWAS analyses rather than in the MR models themselves. To assess the robustness of the findings, we performed several sensitivity analyses. First, Cochran’s Q test was used to evaluate heterogeneity among the IVs [8, 9]. If significant heterogeneity was detected (P < 0.05), we applied a multiplicative random-effects model for validation. Second, the MR-Egger intercept test was used to detect horizontal pleiotropy [10, 11]. When pleiotropy was present (P < 0.05), we employed the MR-PRESSO method to identify and exclude potentially pleiotropic SNPs, and then recalculated pleiotropy-corrected causal estimates. Additionally, we conducted supplementary analyses using the weighted median and MR-Egger methods to further validate the results [10, 11]. Leave-one-out (LOO) analyses identified whether any single SNP drove the causal estimates [8]. |
|  | c) | Describe measurement, quality control and selection of genetic variants | S1 Methods | UK Biobank-based summary statistics for physical frailty were obtained from a recent study based on 386,565 participants of European descent [5]. We extracted a total of 30 highly associated SNPs (P < 5 × 10−8) that were clumped for independence at r2 < 0.001 with a window of 10,000 kb based on European ancestry reference data from the 1000 Genomes Project. These SNPs were used as instrument variables (IVs). For the outcome of SA, IVs were obtained from the Psychiatric Genomics Consortium (PGC) GWAS. GWAS summary data for SA can be applied via the PGC SUI Data Access Portal (https://pgc.unc.edu/for-researchers/data-access-committee/data-access-portal/). The GWAS summary statistics for SA were consistent with those used to construct the polygenic risk score (PRS). The original GWAS included a total of 15 cohorts and represents the largest genome-wide association study of SA to date [6]. To avoid potential sample overlap, we utilized revised summary statistics that excluded the UK Biobank cohort, resulting in a meta-analysis based on the remaining 14 cohorts, comprising 33,353 SA cases and 444,626 controls. Exposure and outcome data were then harmonized to ensure alignment of the effect alleles. This P-value threshold was selected to ensure that each phenotype included at least three independent IVs, thereby meeting the minimum requirements for MR methods such as MR-Egger regression. For causal effect estimation, we primarily applied the inverse-variance weighted (IVW) method [7], which combines ratio estimates of individual variants using the inverse of their variance as weights. Causal effect estimates are reported as odds ratios (ORs) with corresponding 95% confidence intervals (CIs). As this was a two-sample MR analysis based on GWAS summary statistics, covariate adjustment was performed within the original GWAS analyses rather than in the MR models themselves. To assess the robustness of the findings, we performed several sensitivity analyses. First, Cochran’s Q test was used to evaluate heterogeneity among the IVs [8, 9]. If significant heterogeneity was detected (P < 0.05), we applied a multiplicative random-effects model for validation. Second, the MR-Egger intercept test was used to detect horizontal pleiotropy [10, 11]. When pleiotropy was present (P < 0.05), we employed the MR-PRESSO method to identify and exclude potentially pleiotropic SNPs, and then recalculated pleiotropy-corrected causal estimates. Additionally, we conducted supplementary analyses using the weighted median and MR-Egger methods to further validate the results [10, 11]. Leave-one-out (LOO) analyses identified whether any single SNP drove the causal estimates [8]. |
|  | d) | For each exposure, outcome, and other relevant variables, describe methods of assessment and diagnostic criteria for diseases | 11 | IVs for physical frailty were obtained from the UK Biobank GWAS summary statistics [38], and IVs for SA were obtained from the Psychiatric Genomics Consortium (PGC) GWAS used to construct the SA-PRS [25]. |
|  | e) | Provide details of ethics committee approval and participant informed consent, if relevant |  | Does not apply |
| 5 | **Assumptions** | Explicitly state the three core IV assumptions for the main analysis (relevance, independence and exclusion restriction) as well assumptions for any additional or sensitivity analysis | 11 | To ensure the reliability of MR analysis, the study adhered to three core assumptions: (1) instrumental variables (IVs) must be significantly correlated with the exposure factor (correlation assumption); (2) IVs must be independent of confounding factors (independence assumption); and (3) IVs must influence the outcome variable solely through the target exposure (exclusivity assumption). |
| 6 | **Statistical methods: main analysis** | Describe statistical methods and statistics used | S1 Methods | For causal effect estimation, we primarily applied the inverse-variance weighted (IVW) method [7], which combines ratio estimates of individual variants using the inverse of their variance as weights. Causal effect estimates are reported as odds ratios (ORs) with corresponding 95% confidence intervals (CIs). As this was a two-sample MR analysis based on GWAS summary statistics, covariate adjustment was performed within the original GWAS analyses rather than in the MR models themselves. To assess the robustness of the findings, we performed several sensitivity analyses. First, Cochran’s Q test was used to evaluate heterogeneity among the IVs [8, 9]. If significant heterogeneity was detected (P < 0.05), we applied a multiplicative random-effects model for validation. Second, the MR-Egger intercept test was used to detect horizontal pleiotropy [10, 11]. When pleiotropy was present (P < 0.05), we employed the MR-PRESSO method to identify and exclude potentially pleiotropic SNPs, and then recalculated pleiotropy-corrected causal estimates. Additionally, we conducted supplementary analyses using the weighted median and MR-Egger methods to further validate the results [10, 11]. Leave-one-out (LOO) analyses identified whether any single SNP drove the causal estimates [8]. |
|  | a) | Describe how quantitative variables were handled in the analyses (i.e., scale, units, model) | 7 | Following previous studies [20, 29], participants were categorized as non-frailty (0 points), pre-frailty (1-2 points), or frailty (≥ 3 points). |
|  | b) | Describe how genetic variants were handled in the analyses and, if applicable, how their weights were selected | S1 Methods | We extracted a total of 30 highly associated SNPs (P < 5 × 10−8) that were clumped for independence at r2 < 0.001 with a window of 10,000 kb based on European ancestry reference data from the 1000 Genomes Project. |
|  | c) | Describe the MR estimator (e.g. two-stage least squares, Wald ratio) and related statistics. Detail the included covariates and, in case of two-sample MR, whether the same covariate set was used for adjustment in the two samples | S1 Methods | For causal effect estimation, we primarily applied the inverse-variance weighted (IVW) method [7], which combines ratio estimates of individual variants using the inverse of their variance as weights. Causal effect estimates are reported as odds ratios (ORs) with corresponding 95% confidence intervals (CIs). As this was a two-sample MR analysis based on GWAS summary statistics, covariate adjustment was performed within the original GWAS analyses rather than in the MR models themselves. To assess the robustness of the findings, we performed several sensitivity analyses. First, Cochran’s Q test was used to evaluate heterogeneity among the IVs [8, 9]. If significant heterogeneity was detected (P < 0.05), we applied a multiplicative random-effects model for validation. Second, the MR-Egger intercept test was used to detect horizontal pleiotropy [10, 11]. When pleiotropy was present (P < 0.05), we employed the MR-PRESSO method to identify and exclude potentially pleiotropic SNPs, and then recalculated pleiotropy-corrected causal estimates. Additionally, we conducted supplementary analyses using the weighted median and MR-Egger methods to further validate the results [10, 11]. Leave-one-out (LOO) analyses identified whether any single SNP drove the causal estimates [8]. |
|  | d) | Explain how missing data were addressed |  | Used GWAS summary statistics |
|  | e) | If applicable, indicate how multiple testing was addressed |  | Does not apply |
| 7 | **Assessment of assumptions** | Describe any methods or prior knowledge used to assess the assumptions or justify their validity | S1 Methods | Second, the MR-Egger intercept test was used to detect horizontal pleiotropy [10, 11]. When pleiotropy was present (P < 0.05), we employed the MR-PRESSO method to identify and exclude potentially pleiotropic SNPs, and then recalculated pleiotropy-corrected causal estimates. Additionally, we conducted supplementary analyses using the weighted median and MR-Egger methods to further validate the results [10, 11]. |
| 8 | **Sensitivity analyses and additional analyses** | Describe any sensitivity analyses or additional analyses performed (e.g. comparison of effect estimates from different approaches, independent replication, bias analytic techniques, validation of instruments, simulations) | S1 Methods | To assess the robustness of the findings, we performed several sensitivity analyses. First, Cochran’s Q test was used to evaluate heterogeneity among the IVs [8, 9]. If significant heterogeneity was detected (P < 0.05), we applied a multiplicative random-effects model for validation. Second, the MR-Egger intercept test was used to detect horizontal pleiotropy [10, 11]. When pleiotropy was present (P < 0.05), we employed the MR-PRESSO method to identify and exclude potentially pleiotropic SNPs, and then recalculated pleiotropy-corrected causal estimates. Additionally, we conducted supplementary analyses using the weighted median and MR-Egger methods to further validate the results [10, 11]. Leave-one-out (LOO) analyses identified whether any single SNP drove the causal estimates [8]. |
| 9 | **Software and pre-registration** |  |  |  |
|  | a) | Name statistical software and package(s), including version and settings used | S1 Methods | In the primary analysis, we performed two-sample Mendelian randomization (MR) analyses using the TwoSampleMR package in R to evaluate the potential causal relationship between physical frailty and suicidal attempts (SA). |
|  | b) | State whether the study protocol and details were pre-registered (as well as when and where) |  | Does not apply |
|  | **RESULTS** |  |  |  |
| 10 | **Descriptive data** |  |  |  |
|  | a) | Report the numbers of individuals at each stage of included studies and reasons for exclusion. Consider use of a flow diagram |  | Does not apply |
|  | b) | Report summary statistics for phenotypic exposure(s), outcome(s), and other relevant variables (e.g. means, SDs, proportions) | S1 Methods | UK Biobank-based summary statistics for physical frailty were obtained from a recent study based on 386,565 participants of European descent [5]. We extracted a total of 30 highly associated SNPs (P < 5 × 10−8) that were clumped for independence at r2 < 0.001 with a window of 10,000 kb based on European ancestry reference data from the 1000 Genomes Project. These SNPs were used as instrument variables (IVs). For the outcome of SA, IVs were obtained from the Psychiatric Genomics Consortium (PGC) GWAS. GWAS summary data for SA can be applied via the PGC SUI Data Access Portal (https://pgc.unc.edu/for-researchers/data-access-committee/data-access-portal/). The GWAS summary statistics for SA were consistent with those used to construct the polygenic risk score (PRS). The original GWAS included a total of 15 cohorts and represents the largest genome-wide association study of SA to date [6]. To avoid potential sample overlap, we utilized revised summary statistics that excluded the UK Biobank cohort, resulting in a meta-analysis based on the remaining 14 cohorts, comprising 33,353 SA cases and 444,626 controls. Exposure and outcome data were then harmonized to ensure alignment of the effect alleles. This P-value threshold was selected to ensure that each phenotype included at least three independent IVs, thereby meeting the minimum requirements for MR methods such as MR-Egger regression. For causal effect estimation, we primarily applied the inverse-variance weighted (IVW) method [7], which combines ratio estimates of individual variants using the inverse of their variance as weights. Causal effect estimates are reported as odds ratios (ORs) with corresponding 95% confidence intervals (CIs). As this was a two-sample MR analysis based on GWAS summary statistics, covariate adjustment was performed within the original GWAS analyses rather than in the MR models themselves. To assess the robustness of the findings, we performed several sensitivity analyses. First, Cochran’s Q test was used to evaluate heterogeneity among the IVs [8, 9]. If significant heterogeneity was detected (P < 0.05), we applied a multiplicative random-effects model for validation. Second, the MR-Egger intercept test was used to detect horizontal pleiotropy [10, 11]. When pleiotropy was present (P < 0.05), we employed the MR-PRESSO method to identify and exclude potentially pleiotropic SNPs, and then recalculated pleiotropy-corrected causal estimates. Additionally, we conducted supplementary analyses using the weighted median and MR-Egger methods to further validate the results [10, 11]. Leave-one-out (LOO) analyses identified whether any single SNP drove the causal estimates [8]. |
|  | c) | If the data sources include meta-analyses of previous studies, provide the assessments of heterogeneity across these studies |  | Does not apply |
|  | d) | For two-sample MR:  i.  Provide justification of the similarity of the genetic variant-exposure associations between the exposure and outcome samples  ii.  Provide information on the number of individuals who overlap between the exposure and outcome studies | S1 Methods | The GWAS summary statistics for SA were consistent with those used to construct the polygenic risk score (PRS). The original GWAS included a total of 15 cohorts and represents the largest genome-wide association study of SA to date [6]. To avoid potential sample overlap, we utilized revised summary statistics that excluded the UK Biobank cohort, resulting in a meta-analysis based on the remaining 14 cohorts, comprising 33,353 SA cases and 444,626 controls. |
| 11 | **Main results** |  |  |  |
|  | a) | Report the associations between genetic variant and exposure, and between genetic variant and outcome, preferably on an interpretable scale |  | Does not apply |
|  | b) | Report MR estimates of the relationship between exposure and outcome, and the measures of uncertainty from the MR analysis, on an interpretable scale, such as odds ratio or relative risk per SD difference | 15 and 17 | In secondary analyses, MR results were directionally consistent with the findings from the cohort analysis, showing that genetically determined frailty was associated with an increased risk of SA (OR = 2.06, 95% CI: 1.21-3.52, P = 0.008) (Figure 1). Scatter plots and LOO plots are presented in S5 Fig. Estimates from MR analyses using the MR-Egger, weighted median, random-effects IVW method, MR-Egger intercept test for horizontal pleiotropy, and heterogeneity tests with Cochran’s Q statistic are provided in the S19 and S20 Tables. |
|  | c) | If relevant, consider translating estimates of relative risk into absolute risk for a meaningful time period |  | Does not apply |
|  | d) | Consider plots to visualize results (e.g. forest plot, scatterplot of associations between genetic variants and outcome versus between genetic variants and exposure) |  | Figure 1. Two-Sample MR analyses for the causal associations of genetically predicted physical frailty with the risk of suicide attempts |
| 12 | **Assessment of assumptions** |  |  |  |
|  | a) | Report the assessment of the validity of the assumptions |  | S5 Fig. Scatter plot and leave-one-out test for the causal association between physical frailty and suicide attempts.  S19 Table. Estimates from MR analysis using the IVW method for the association between frailty and SA, and replicated estimates from MR analyses using the MR-Egger regression and weighted median methods for the same association.  S20 Table. Replicated estimates from MR analysis using the random-effects IVW method for the association between frailty and SA, along with MR-Egger intercept test results for horizontal pleiotropy and heterogeneity test results using Cochran’s Q statistic. |
|  | b) | Report any additional statistics (e.g., assessments of heterogeneity across genetic variants, such as *I^2^*, Q statistic or E-value) |  | S5 Fig. Scatter plot and leave-one-out test for the causal association between physical frailty and suicide attempts.  S19 Table. Estimates from MR analysis using the IVW method for the association between frailty and SA, and replicated estimates from MR analyses using the MR-Egger regression and weighted median methods for the same association.  S20 Table. Replicated estimates from MR analysis using the random-effects IVW method for the association between frailty and SA, along with MR-Egger intercept test results for horizontal pleiotropy and heterogeneity test results using Cochran’s Q statistic. |
| 13 | **Sensitivity analyses and additional analyses** |  |  |  |
|  | a) | Report any sensitivity analyses to assess the robustness of the main results to violations of the assumptions | 17 | Scatter plots and LOO plots are presented in S5 Fig. Estimates from MR analyses using the MR-Egger, weighted median, random-effects IVW method, MR-Egger intercept test for horizontal pleiotropy, and heterogeneity tests with Cochran’s Q statistic are provided in the S19 and S20 Tables. |
|  | b) | Report results from other sensitivity analyses or additional analyses | 17 | Scatter plots and LOO plots are presented in S5 Fig. Estimates from MR analyses using the MR-Egger, weighted median, random-effects IVW method, MR-Egger intercept test for horizontal pleiotropy, and heterogeneity tests with Cochran’s Q statistic are provided in the S19 and S20 Tables. |
|  | c) | Report any assessment of direction of causal relationship (e.g., bidirectional MR) |  | Does not apply |
|  | d) | When relevant, report and compare with estimates from non-MR analyses |  | Presented in the Results section. |
|  | e) | Consider additional plots to visualize results (e.g., leave-one-out analyses) | 17 | Scatter plots and LOO plots are presented in S5 Fig. Estimates from MR analyses using the MR-Egger, weighted median, random-effects IVW method, MR-Egger intercept test for horizontal pleiotropy, and heterogeneity tests with Cochran’s Q statistic are provided in the S19 and S20 Tables. |
|  | **DISCUSSION** |  |  |  |
| 14 | **Key results** | Summarize key results with reference to study objectives | 17 | Two-sample MR analyses provided complementary genetic evidence supporting the observed association. |
| 15 | **Limitations** | Discuss limitations of the study, taking into account the validity of the IV assumptions, other sources of potential bias, and imprecision. Discuss both direction and magnitude of any potential bias and any efforts to address them | 21 and 22 | Notably, subsequent two-step MR analyses did not provide statistically significant evidence supporting a causal mediating role for these biomarkers. The discrepancy between the observational mediation and MR findings may reflect several factors. First, genetic instruments for circulating biomarkers typically explain limited phenotypic variance, substantially reducing statistical power in MR analyses, particularly for rare and etiologically heterogeneous outcomes such as SA. |
| 16 | **Interpretation** |  |  |  |
|  | a) | Meaning: Give a cautious overall interpretation of results in the context of their limitations and in comparison with other studies | 18, 19, and 21 | Although a recent review suggested that frailty may be an important underlying factor in SA during later life, the evidence is primarily based on qualitative studies [16], with only one quantitative study available [15]. Using follow-up data from a large-scale cohort of U.S. veterans aged 65 years and older, Kuffel et al. demonstrated that a cumulative FI, composed of dozens of deficits, was associated with a greater than 40% increased risk of SA, supporting our findings [15, 20]. However, given the complexity and high cost of FI measurements, the user-friendly FP provides a more practical alternative for clinical and large-scale epidemiologic research. Despite its significance, there is still a notable gap in research investigating the relationship between FP and SA. The present study revealed, for the first time, that pre-frailty and frailty, as measured by FP, are significantly associated with an increased risk of developing SA. This association persisted even after adjusting for sociodemographic and lifestyle factors, psychiatric disorders, CVD, cancer, and genetic risk, as well as in sensitivity and subgroup analyses. Two-sample MR analyses provided additional support for the genetic association between FP-measured physical frailty and SA, suggesting that the observed association may be consistent with a potential causal relationship, while accounting for residual confounding and reverse causation. Notably, subsequent two-step MR analyses did not provide statistically significant evidence supporting a causal mediating role for these biomarkers. The discrepancy between the observational mediation and MR findings may reflect several factors. First, genetic instruments for circulating biomarkers typically explain limited phenotypic variance, substantially reducing statistical power in MR analyses, particularly for rare and etiologically heterogeneous outcomes such as SA. Second, the biomarkers identified in observational analyses may primarily represent downstream correlates of frailty or shared biological processes, rather than direct causal mediators in the frailty-SA pathway. Therefore, these findings should be interpreted as indicating potential biological pathways rather than confirmed causal intermediates. |
|  | b) | Mechanism: Discuss underlying biological mechanisms that could drive a potential causal relationship between the investigated exposure and the outcome, and whether the gene-environment equivalence assumption is reasonable. Use causal language carefully, clarifying that IV estimates may provide causal effects only under certain assumptions | 20-22 | Presented in the paragraph 5 of discussion section. |
|  | c) | Clinical relevance: Discuss whether the results have clinical or public policy relevance, and to what extent they inform effect sizes of possible interventions | 24 | Incorporating frailty assessment and management into primary suicide prevention strategies may facilitate the identification of high-risk individuals and inform future research into integrated biological and psychosocial interventions. |
| 17 | **Generalizability** | Discuss the generalizability of the study results (a) to other populations, (b) across other exposure periods/timings, and (c) across other levels of exposure | 23 | Third, as the study population comprised only White participants, further research is required to validate these findings in diverse racial and ethnic groups. Additionally, the lack of validation in an independent dataset is a limitation. |
|  | **OTHER INFORMATION** |  |  |  |
| 18 | **Funding** | Describe sources of funding and the role of funders in the present study and, if applicable, sources of funding for the databases and original study or studies on which the present study is based | 25 | BPL was supported by Natural Science Foundation of Shandong Province [No: ZR2021QH310] and Young Scholars Program of Shandong University. CXJ was supported by the National Natural Science Foundation of China [No: 82473710]. The funding organizations had no role in the design and conduct of the study; collection, management, analysis, and interpretation of the data; preparation, review, or approval of the manuscript; and decision to submit the manuscript for publication. |
| 19 | **Data and data sharing** | Provide the data used to perform all analyses or report where and how the data can be accessed, and reference these sources in the article. Provide the statistical code needed to reproduce the results in the article, or report whether the code is publicly accessible and if so, where | 24 | Data are available in a public, open access repository. This research has been conducted using the UK Biobank Resource under Application Number 91536. The UK Biobank data are available on application to the UK Biobank (www.ukbiobank.ac.uk/) with access fees. |
| 20 | **Conflicts of Interest** | All authors should declare all potential conflicts of interest | 24 | All authors declare that they have no competing interests. |

This checklist is copyrighted by the Equator Network under the Creative Commons Attribution 3.0 Unported (CC BY 3.0) license.

1. Skrivankova VW, Richmond RC, Woolf BAR, Yarmolinsky J, Davies NM, Swanson SA, et al. Strengthening the Reporting of Observational Studies in Epidemiology using Mendelian Randomization (STROBE-MR) Statement. JAMA. 2021;under review.

2. Skrivankova VW, Richmond RC, Woolf BAR, Davies NM, Swanson SA, VanderWeele TJ, et al. Strengthening the Reporting of Observational Studies in Epidemiology using Mendelian Randomisation (STROBE-MR): Explanation and Elaboration. BMJ. 2021;375:n2233.
