# Supplementary material for: Physical frailty, genetic risk, mediating biomarkers, and risk of suicide attempt: A prospective cohort study
Source: PLoS Med. 2026 Apr 6;23(4):e1005045. doi: 10.1371/journal.pmed.1005045 (PMC13065332; doi:10.1371/journal.pmed.1005045)
Supplement: S1 Text — (DOCX) [file pmed.1005045.s003.docx]

**S1 Text.** **Supplementary methods.**

**Blood biomarkers**

Blood samples were obtained from the consenting participants at baseline, processed into individual components, and stored at the UK Biobank’s facilities at a temperature of −80 ^◦^C or in a nitrogen atmosphere until analysis. The UK Biobank confirms the reliability of blood biomarkers through rigorous quality control measures and provides comprehensive information on assay performance at <https://biobank.ndph.ox.ac.uk/showcase/showcase/docs/serum_biochemistry.pdf> and a prior publication [1]. The selection of biomarkers that might mediate the associations between frailty and suicide attempt (SA) was based on the knowledge of relevant literature [2-4]. Finally, a total of 61 blood biomarkers were selected, including liver function [albumin, alanine aminotransferase, aspartate aminotransferase, direct bilirubin, gamma glutamyltransferase, total protein, and total bilirubin], renal function [phosphate, cystatin C, creatinine, urea, and urate], immunometabolic [apolipoprotein A, apolipoprotein B, cholesterol, glucose, gycated hemoglobin, high density lipoprotein cholesterol, low density lipoprotein direct, lipoprotein A, C-reactive protein, and triglycerides], endocrine (IGF-1, oestradiol, SHBG, and testosterone), bone and joint [alkaline phosphatase, calcium, rheumatoid factor, and vitamin D], red blood cell [red blood cell count, hemoglobin concentration, hematocrit percentage, mean corpuscular volume, mean corpuscular hemoglobin, mean corpuscular hemoglobin concentration, red blood cell distribution width, nucleated red blood cell count, nucleated red blood cell percentage, reticulocyte percentage, reticulocyte count, mean reticulocyte volume, mean sphered cell volume, immature reticulocyte fraction, high light scatter reticulocyte percentage, and high light scatter reticulocyte percentage count], white blood cell [white blood cell count, lymphocyte count, monocyte count, neutrophil count, eosinophil count, basophil count, lymphocyte percentage, monocyte percentage, neutrophil percentage, eosinophil percentage, and basophil percentage], and platelet (platelet count, platelet crit, mean platelet volume, and platelet distribution width).

**Mendelian randomization analyses**

In the primary analysis, we performed two-sample Mendelian randomization (MR) analyses using the TwoSampleMR package in R to evaluate the potential causal relationship between physical frailty and SA. UK Biobank-based summary statistics for physical frailty were obtained from a recent study based on 386,565 participants of European descent [5]. We extracted a total of 30 highly associated SNPs (*P* < 5 × 10^−8^) that were clumped for independence at r^2^ < 0.001 with a window of 10,000 kb based on European ancestry reference data from the 1000 Genomes Project. These SNPs were used as instrument variables (IVs). For the outcome of SA, IVs were obtained from the Psychiatric Genomics Consortium (PGC) GWAS. GWAS summary data for SA can be applied via the PGC SUI Data Access Portal (<https://pgc.unc.edu/for-researchers/data-access-committee/data-access-portal/>). The GWAS summary statistics for SA were consistent with those used to construct the polygenic risk score (PRS). The original GWAS included a total of 15 cohorts and represents the largest genome-wide association study of SA to date [6]. To avoid potential sample overlap, we utilized revised summary statistics that excluded the UK Biobank cohort, resulting in a meta-analysis based on the remaining 14 cohorts, comprising 33,353 SA cases and 444,626 controls. Exposure and outcome data were then harmonized to ensure alignment of the effect alleles. This *P*-value threshold was selected to ensure that each phenotype included at least three independent IVs, thereby meeting the minimum requirements for MR methods such as MR-Egger regression. For causal effect estimation, we primarily applied the inverse-variance weighted (IVW) method [7], which combines ratio estimates of individual variants using the inverse of their variance as weights. Causal effect estimates are reported as odds ratios (ORs) with corresponding 95% confidence intervals (CIs). As this was a two-sample MR analysis based on GWAS summary statistics, covariate adjustment was performed within the original GWAS analyses rather than in the MR models themselves. To assess the robustness of the findings, we performed several sensitivity analyses. First, Cochran’s Q test was used to evaluate heterogeneity among the IVs [8, 9]. If significant heterogeneity was detected (*P* < 0.05), we applied a multiplicative random-effects model for validation. Second, the MR-Egger intercept test was used to detect horizontal pleiotropy [10, 11]. When pleiotropy was present (*P* < 0.05), we employed the MR-PRESSO method to identify and exclude potentially pleiotropic SNPs, and then recalculated pleiotropy-corrected causal estimates. Additionally, we conducted supplementary analyses using the weighted median and MR-Egger methods to further validate the results [10, 11]. Leave-one-out (LOO) analyses identified whether any single SNP drove the causal estimates [8].

**Two‐Step Mendelian randomization analyses**

A two-step Mendelian randomization (MR) framework was applied to investigate whether the biomarker identified in the epidemiological mediation analysis potentially mediates the association between frailty and SA. This approach allows the total effect to be decomposed into two sequential causal components and provides complementary genetic evidence for potential mediation pathways [12]. In the first step, we estimated the causal effect of frailty (exposure) on the biomarker (intermediate phenotype) using two-sample MR. In the second step, we evaluated the causal effect of the biomarker (exposure) on SA (outcome). Genetic instruments for the biomarker were derived from large-scale GWAS using SNPs reaching genome-wide significance (*P* < 5 × 10^−8^) [13, 14]. These SNPs were clumped for linkage disequilibrium based on European ancestry reference data from the 1000 Genomes Project, applying an r² threshold of < 0.001 and a window size of 10,000 kb to ensure independence among instruments. For both steps, causal effect estimates were primarily derived using the inverse-variance weighted (IVW) method. To account for potential heterogeneity among instrumental variables, we additionally applied the multiplicative random-effects IVW model (IVW-MRE). To assess the robustness of the findings under different assumptions regarding horizontal pleiotropy, we conducted complementary analyses using the MR-Egger regression and weighted median methods. These approaches provide valid causal estimates under varying pleiotropy structures, assuming that pleiotropic effects are independent of instrument strength or that at least half of the instrumental weight is contributed by valid instruments. All MR analyses were performed using the TwoSampleMR package in R. Consistent criteria for instrument selection, harmonization of exposure and outcome alleles, and quality control were applied across both steps to ensure comparability. The two-step MR results were interpreted jointly to evaluate whether genetic evidence supported a causal mediating role of the biomarker in the frailty-SA pathway.

**Reference**

[1] Elliott P, Peakman TC. The UK Biobank sample handling and storage protocol for the collection, processing and archiving of human blood and urine. Int J Epidemiol. 2008; 37(2):234-244.

[2]Zhang B, You J, Rolls ET, et al. Identifying behaviour-related and physiological risk factors for suicide attempt in the UK Biobank. Nat Hum Behav. 2024; 8(9):1784-1797.

[3] Hoogendijk EO, Afilalo J, Ensrud KE, et al. Frailty: implications for clinical practice and public health. Lancet. 2019; 394(10206):1365-1375.

[4] Mishra M, Wu J, Kane AE, Howlett SE. The intersection of frailty and metabolism. Cell Metab, 2024, 36(5):893-911.

[5]Jiang R, Noble S, Rosenblatt M, Dai W, Ye J, Liu S, et al. The brain structure, inflammatory, and genetic mechanisms mediate the association between physical frailty and depression. Nat Commun. 2024; 15(1):4411.

[6]Docherty AR, Mullins N, Ashley-Koch AE, et al. GWAS Meta-Analysis of Suicide Attempt: Identification of 12 Genome-Wide Significant Loci and Implication of Genetic Risks for Specific Health Factors. Am J Psychiatry. 2023; 180(10):723-738.

[7] Burgess S, Butterworth A, Thompson SG. Mendelian randomization analysis with multiple genetic variants using summarized data. Genet Epidemiol. 2013;37(7):658-665.

[8] Burgess S, Thompson SG. Interpreting findings from Mendelian randomization using the MR-Egger method. Eur J Epidemiol. 2017;32(5):377-389.

[9] Greco MF, Minelli C, Sheehan NA, Thompson JR. Detecting pleiotropy in Mendelian randomisation studies with summary data and a continuous outcome. Stat Med. 2015;34(21):2926-2940.

[10] Bowden J, Davey Smith G, Burgess S. Mendelian randomization with invalid instruments: effect estimation and bias detection through Egger regression. Int J Epidemiol. 2015;44(2):512-525.

[11] Bowden J, Davey Smith G, Haycock PC, Burgess S. Consistent Estimation in Mendelian Randomization with Some Invalid Instruments Using a Weighted Median Estimator. Genet Epidemiol. 2016;40(4):304-314.

[12] Carter AR, Sanderson E, Hammerton G, Richmond RC, Davey Smith G, Heron J, et al. Mendelian randomisation for mediation analysis: current methods and challenges for implementation. Eur j Epidemiol. 2021;36:465-78.

[13] Vuckovic D, Bao EL, Akbari P, et al. The polygenic and monogenic basis of blood traits and diseases. Cell. 2020;182(5):1214-1231.e11.

[14] Sinnott-Armstrong N, Tanigawa Y, Amar D, et al. Genetics of 35 blood and urine biomarkers in the UK Biobank. Nat Genet. 2021;53(2):185-194.
