# Supplementary material for: Physical frailty, genetic risk, mediating biomarkers, and risk of suicide attempt: A prospective cohort study
Source: PLoS Med. 2026 Apr 6;23(4):e1005045. doi: 10.1371/journal.pmed.1005045 (PMC13065332; doi:10.1371/journal.pmed.1005045)
Supplement: S1 Appendix — Fig A. Flowchart of the selection of the study population from the UK Biobank study. Fig B. Directed acyclic graph depicting the hypothesized causal structure underlying the association between physical frailty and suicide attempt. Sociodemographic factors included age, sex, educational level, employment status, and Townsend Deprivation Index; lifestyle factors included smoking status, drinking frequency, and body mass index; medical histories included cardiovascular diseases, psychiatric disorders, and cancer. Fig C. Crude cumulative incidence of suicide attempt by frailty status. SA, suicide attempt. The P value was estimated using the log-rank test. Fig D. Dose-response associations between frailty scores and the risk of suicide attempt. SA, suicide attempt; CI, confidence interval; HR, hazard ratio. Model was adjusted for age, sex, education, employment, Townsend Deprivation Index, drinking frequency, smoking status, body mass index, genetic risk, preexisting psychiatric disorders, cardiovascular diseases, and cancer. P-values for overall and nonlinear associations were estimated using restricted cubic spline analysis based on the Cox proportional hazards model. Fig E. Scatter plot and leave-one-out test for the causal association between physical frailty and suicide attempt. MR, Mendelian randomization; IVW, inverse-variance weighted; SNP, single nucleotide polymorphism. Causal effect estimates in the scatter plot were obtained using the IVW method, IVW with multiplicative random-effects, MR-Egger regression, and the weighted median method. Corresponding statistical significance was assessed using these respective methods. The leave-one-out analysis was conducted using the IVW method, with each SNP sequentially removed. Horizontal lines represent 95% confidence intervals. (DOCX) [file pmed.1005045.s004.docx]

**S1 Appendix**

[**Fig A. Flowchart of the selection of the study population from the UK Biobank study 1**](#_Toc225718944)

[**Fig B. Directed acyclic graph depicting the hypothesized causal structure underlying the association between physical frailty and suicide attempt 2**](#_Toc225718945)

[**Fig C. Crude cumulative incidence of suicide attempt by frailty status 3**](#_Toc225718946)

[**Fig D. Dose-response associations between the frailty score and the risk of suicide attempt 4**](#_Toc225718947)

[**Fig E. Scatter plot and leave-one-out test for the causal association between physical frailty and suicide attempt 5**](#_Toc225718948)


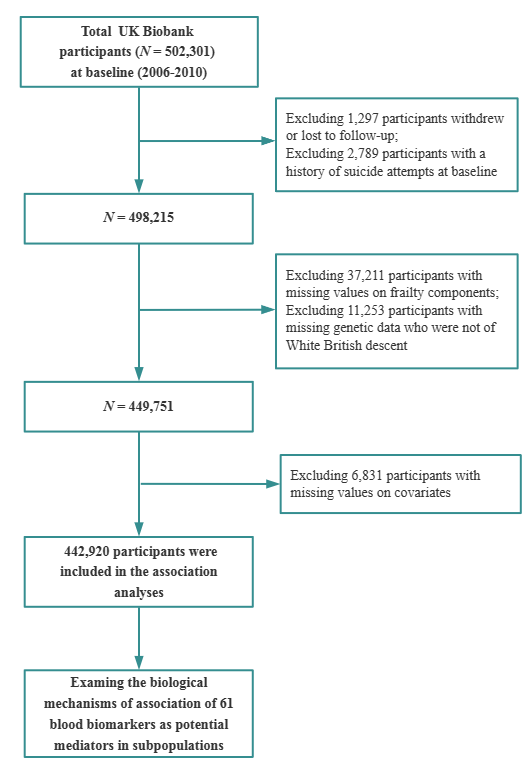


**Fig A. Flowchart of the selection of the study population from the UK Biobank study**

**
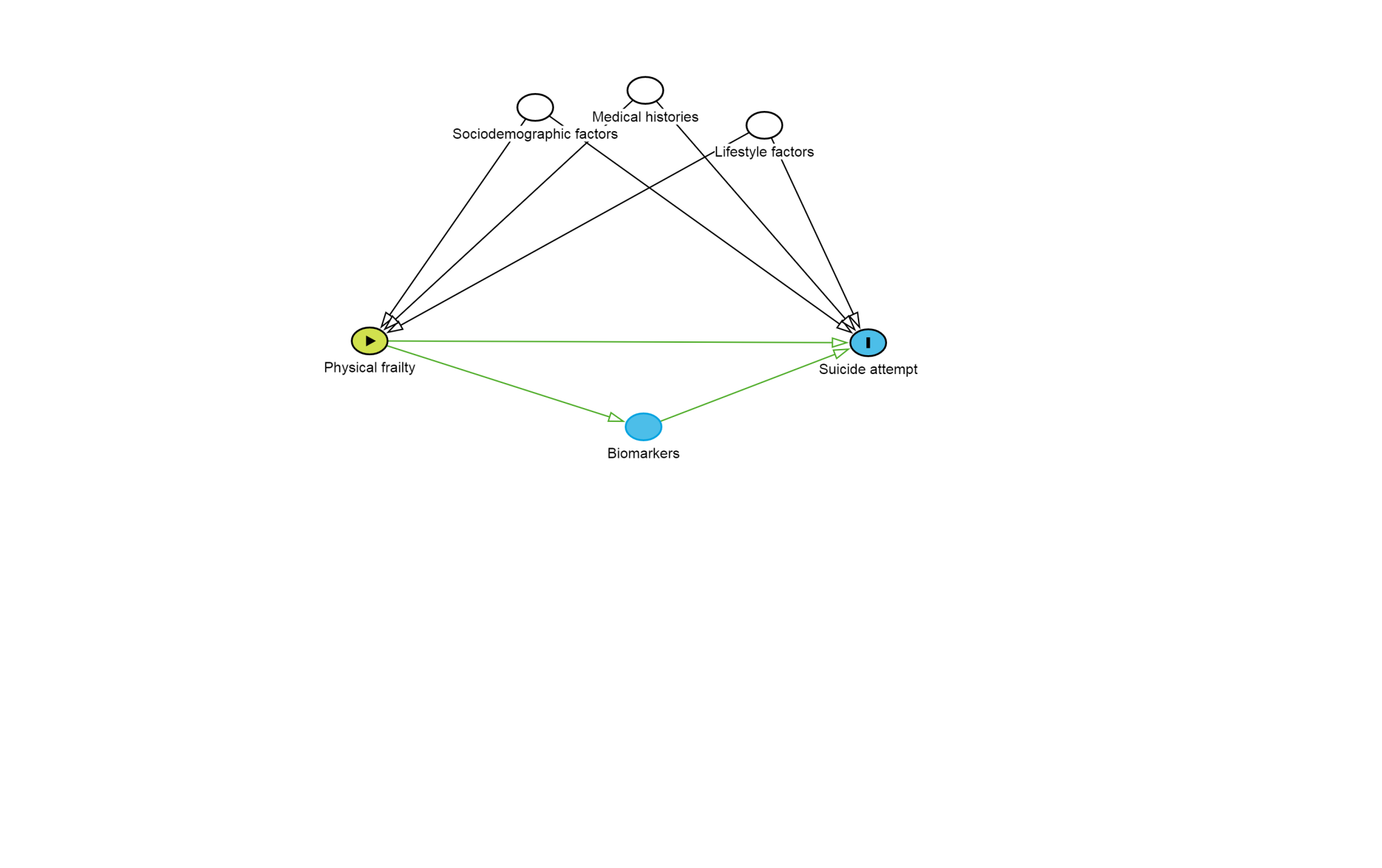
**

**Fig B. Directed acyclic graph depicting the hypothesized causal structure underlying the association between physical frailty and suicide attempt**

*Note*: Sociodemographic factors included age, sex, educational level, employment status, and Townsend Deprivation Index; lifestyle factors included smoking status, drinking frequency, and body mass index; medical histories included cardiovascular diseases, psychiatric disorders, and cancer.


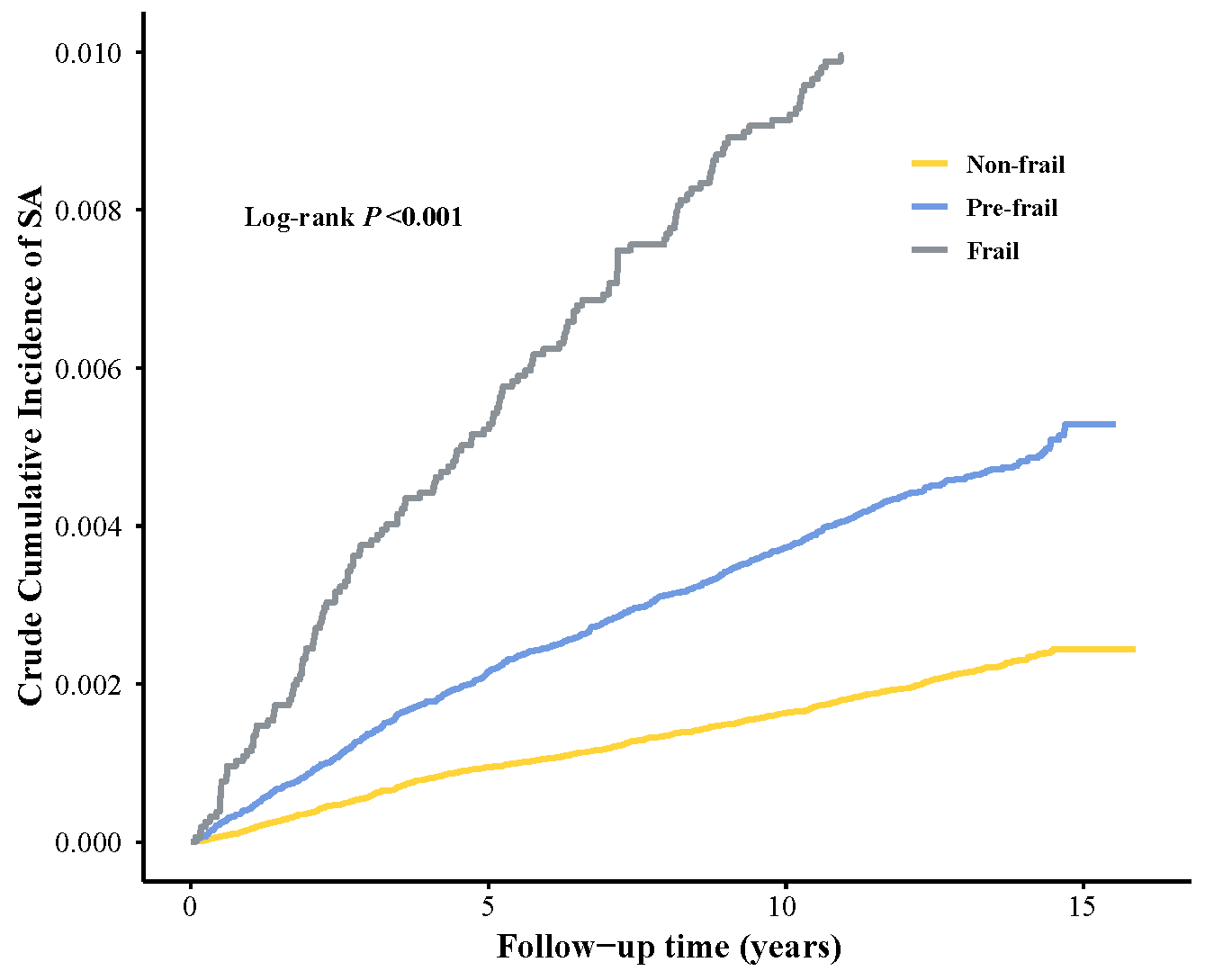


**Fig C. Crude cumulative incidence of suicide attempt by frailty status**

*Note*: the *P* value was estimated using the log-rank test. Abbreviations: SA, suicide attempt.

**
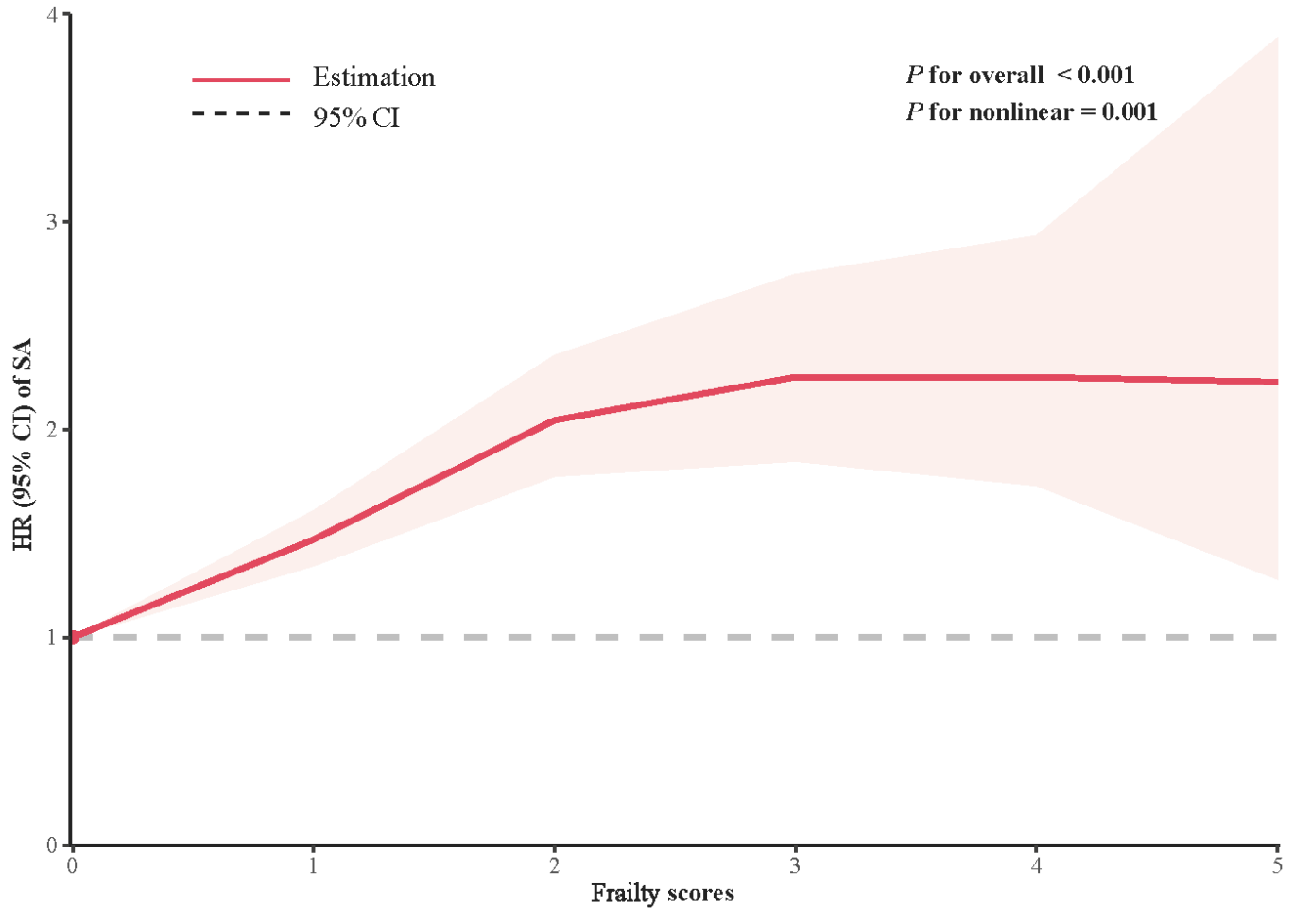
**

**Fig D. Dose-response associations between the frailty score and the risk of suicide attempt**

*Note*: *P*-values for overall and nonlinear associations were estimated using restricted cubic spline analysis based on the Cox proportional hazards model. Abbreviations: SA, suicide attempt; CI, confidence interval; HR, hazard ratio. *Note*: Model was adjusted for age, sex, education, employment, Townsend Deprivation Index, drinking frequency, smoking status, body mass index, genetic risk, pre-existing psychiatric disorders, cardiovascular diseases, and cancer.

**Fig E**. **Scatter plot and leave-one-out test for the causal association between physical frailty and suicide attempt**

Abbreviations: MR, Mendelian randomization; IVW, inverse-variance weighted; SNP, single nucleotide polymorphism. Note: Causal effect estimates in the scatter plot were obtained using the IVW method, IVW with multiplicative random effects, MR-Egger regression, and the weighted median method. Corresponding statistical significance was assessed using these respective methods. The leave-one-out analysis was conducted using the IVW method, with each SNP sequentially removed. Horizontal lines represent 95% confidence intervals.
