# Supplementary material for: Physical frailty, genetic risk, mediating biomarkers, and risk of suicide attempt: A prospective cohort study
Source: PLoS Med. 2026 Apr 6;23(4):e1005045. doi: 10.1371/journal.pmed.1005045 (PMC13065332; doi:10.1371/journal.pmed.1005045)
Supplement: S2 Appendix — Table A. Frailty definition and cutoff values in the UK Biobank. Table B. Detailed information on selected blood biomarkers in the study. Table C. Measures of covariates at baseline in the UK Biobank. Table D. Summary of missing data for covariates. Table E. Detailed information on selected genetic variants and their associations with the exposure and outcome. Table F. List of the 17 comorbidities, assigned weights and associated ICD-10 codes for construction of the Charlson Comorbidity Index. Table G. Baseline characteristics according to suicide attempt status. Table H. Risk of suicide attempt according to frailty status within genetic risk categories. Table I. RERI and AP for additive interaction between frailty status and genetic risk. Table J. Multivariable-adjusted Cox regression models for the association of blood biomarkers with suicide attempt. Table K. Multivariable-adjusted linear regression models for the associations between frailty scores and suicide attempt-related blood biomarkers. Table L. Two-step Mendelian randomization analyses evaluating the potential causal roles of candidate biomarkers in the association between physical frailty and suicide attempt. Table M. Associations of frailty status with the risk of suicide attempt after excluding the cases that occurred within the first 2-years of follow-up. Table N. Associations of frailty status with the risk of suicide attempt after using multiple imputation for missing covariates. Table O. Associations of frailty status with the risk of suicide attempt after excluding participants with baseline CVD, cancer, or higher deprivation. Table P. Associations of frailty status with the risk of suicide attempt after further adjustment for the Charlson Comorbidity Index. Table Q. E-values for the associations between frailty status and suicide attempt. Table R. Associations of frailty status with the risk of suicide attempt accounting for death as a competing risk. Table S. Stratified analysis for the associa [file pmed.1005045.s005.docx]

**S2 Appendix**

[**Table A. Frailty definition and cut-off values in the UK Biobank 1**](#_Toc225718778)

[**Table B. Detailed information on selected blood biomarkers in the study 4**](#_Toc225718779)

[**Table C. Measures of covariates at baseline in the UK Biobank. 7**](#_Toc225718780)

[**Table D. Summary of missing data for covariates 9**](#_Toc225718781)

[**Table E. Detailed information on selected genetic variants and their associations with the exposure and outcome 10**](#_Toc225718782)

[**Table F. List of the 17 comorbidities, assigned weights and associated ICD-10 codes for construction of the Charlson Comorbidity Index 11**](#_Toc225718783)

[**Table G. Baseline characteristics according to suicide attempt status 12**](#_Toc225718784)

[**Table H. Risk of suicide attempt according to frailty status within genetic risk categories 14**](#_Toc225718785)

[**Table I. RERI and AP for additive interaction between frailty status and genetic risk 15**](#_Toc225718786)

[**Table J. Multivariable-adjusted Cox regression models for the association of blood biomarkers with suicide attempt 16**](#_Toc225718787)

[**Table K. Multivariable-adjusted linear regression models for the associations between frailty scores and suicide attempt-related blood biomarkers 19**](#_Toc225718788)

[**Table L. Two-step Mendelian randomization analyses evaluating the potential causal roles of candidate biomarkers in the association between physical frailty and suicide attempt 21**](#_Toc225718789)

[**Table M. Associations of frailty status with the risk of suicide attempt after excluding the cases occurred within the first two-years of follow-up. 26**](#_Toc225718790)

[**Table N. Associations of frailty status with the risk of suicide attempt after using multiple imputation for missing covariates. 27**](#_Toc225718791)

[**Table O. Associations of frailty status with the risk of suicide attempt after excluding participants with baseline CVD, cancer, psychiatric disorders, or higher deprivation. 28**](#_Toc225718792)

[**Table P. Associations of frailty status with the risk of suicide attempt after further adjustment for the Charlson Comorbidity Index. 29**](#_Toc225718793)

[**Table Q. E-values for the associations between frailty status and suicide attempt. 30**](#_Toc225718794)

[**Table R. Associations of frailty status with the risk of suicide attempt accounting for death as a competing risk. 31**](#_Toc225718795)

[**Table S. Stratified analysis for the association of the frailty status with the risk of suicide attempt. 32**](#_Toc225718796)

[**Table T. Estimates from MR analysis using the IVW method for the association between frailty and suicide attempt, and replicated estimates from MR analyses using the MR-Egger regression and weighted median methods for the same association 34**](#_Toc225718797)

[**Table U. Replicated estimates from MR analysis using the random-effects IVW method for the association between frailty and suicide attempt, along with MR-Egger intercept test results for horizontal pleiotropy and heterogeneity test results using Cochran’s Q statistic. 35**](#_Toc225718798)

[**Table V. Associations between physical frailty and suicidal ideation, and mediating role of suicidal ideation in the frailty-SA association 36**](#_Toc225718799)

[**Table W. Comparison of baseline characteristics between included and excluded populations 37**](#_Toc225718800)

**Table A. Frailty definition and cut-off values in the UK Biobank**

| **Individual components** | **Criteria** | **Field IDs** |
| --- | --- | --- |
| **Weight loss** | “Compared with one year ago, has your weight changed?”  Response:   - Yes, lost weight = 1; - Other = 0; - Prefer not to answer = missing data. | 2306 |
| **Exhaustion** | “Over the past two weeks, how often have you felt tired or had little energy?”  Response:   - More than half the days or nearly every day = 1; - Other = 0; - Prefer not to answer = missing data. | 2080 |
| **Low physical activity** | “In the last 4 weeks did you spend any time doing the following? (You can select more than one answer)”  Response:   - Walking for pleasure (not as a means of transport) = 0; - Other exercises (eg: swimming, cycling, keep fit, bowling) = 0; - Strenuous sports = 0; - Light DIY (eg: pruning, watering the lawn): - Frequency of once per week or less=1; - Frequency of more than once per week=0; - Heavy DIY (eg: weeding, lawn mowing, carpentry, digging) = 0; - None of the above = 1; - Prefer not to answer = missing data. | 6164, 1011 |
| **Slow gait speed** | “How would you describe your usual walking pace?”  Response:   - Slow = 1; - Other = 0; - Prefer not to answer = missing data. | 924 |
| **Low grip strength** | Measured grip strength expressed in kg by sex- and BMI- adjusted cut-off points.  Cut-off points:  Men   - If BMI ≤24.0 kg/m^2^ & grip strength ≤29 kg - If 24.0< BMI ≤28.0 kg/m^2^ & grip strength ≤30 kg - If BMI >28.0 kg/m^2^ & grip strength ≤32 kg   Women   - If BMI ≤23.0 kg/m^2^ & grip strength ≤17 kg - If 23.0< BMI ≤26.0 kg/m^2^ & grip strength ≤17.3 kg - If 26.0< BMI ≤29.0 kg/m^2^ & grip strength ≤18 kg - If BMI >29.0 kg/m^2^ & grip strength ≤21 kg - If data on BMI or grip strength is not available = missing data. | 31, 21001, 46, 47 |

Abbreviations: BMI, body mass index.

**Table B. Detailed information on selected blood biomarkers in the study**

| **Blood Biomarkers** | **Measurement unit** | ***N*** | **Category** | **Field IDs in UK biobank** |
| --- | --- | --- | --- | --- |
| **Liver function** |  |  |  |  |
| Albumin | g/L | 386,928 | Blood biochemistry | 30600 |
| Alanine aminotransferase | U/L | 422,505 | Blood biochemistry | 30620 |
| Aspartate aminotransferase | U/L | 421,087 | Blood biochemistry | 30650 |
| Direct bilirubin | umol/L | 359,838 | Blood biochemistry | 30660 |
| Gamma glutamyltransferase | U/L | 422,454 | Blood biochemistry | 30730 |
| Total bilirubin | umol/L | 420,874 | Blood biochemistry | 30840 |
| Total protein | g/L | 386,497 | Blood biochemistry | 30860 |
| **Renal function** |  |  |  |  |
| Urea | mmol/L | 422,379 | Blood biochemistry | 30670 |
| Creatinine | umol/L | 422,457 | Blood biochemistry | 30700 |
| Cystatin C | mg/L | 422,631 | Blood biochemistry | 30720 |
| Phosphate | mmol/L | 386,180 | Blood biochemistry | 30810 |
| Urate | umol/L | 422,157 | Blood biochemistry | 30880 |
| **Immunometabolic** |  |  |  |  |
| Apolipoprotein A | g/L | 384,614 | Blood biochemistry | 30630 |
| Apolipoprotein B | g/L | 420,539 | Blood biochemistry | 30640 |
| Cholesterol | mmol/L | 422,658 | Blood biochemistry | 30690 |
| Glucose | mmol/L | 386,468 | Blood biochemistry | 30740 |
| Glycated haemoglobin (HbA1c) | mmol/mol | 421,434 | Blood biochemistry | 30750 |
| HDL cholesterol | mmol/L | 386,739 | Blood biochemistry | 30760 |
| LDL direct | mmol/L | 421,869 | Blood biochemistry | 30780 |
| Lipoprotein A | nmol/L | 337,928 | Blood biochemistry | 30790 |
| C-reactive protein | mg/L | 421,767 | Blood biochemistry | 30710 |
| Triglycerides | mmol/L | 422,331 | Blood biochemistry | 30870 |
| **Endocrine** |  |  |  |  |
| IGF-1 | nmol/L | 420,345 | Blood biochemistry | 30770 |
| Oestradiol | pmol/L | 69,213 | Blood biochemistry | 30800 |
| SHBG | nmol/L | 383,207 | Blood biochemistry | 30830 |
| Testosterone | nmol/L | 383,046 | Blood biochemistry | 30850 |
| **Bone and joint** |  |  |  |  |
| Alkaline phosphatase | U/L | 422,678 | Blood biochemistry | 30610 |
| Calcium | mmol/L | 386,793 | Blood biochemistry | 30680 |
| Rheumatoid factor | IU/ml | 37,193 | Blood biochemistry | 30820 |
| Vitamin D | nmol/L | 403,985 | Blood biochemistry | 30890 |
| **Red blood cell** |  |  |  |  |
| Red blood cell (erythrocyte) count | 10^12 cells/Litre | 429,982 | Blood count | 30010 |
| Hemoglobin concentration | grams/decilitre | 429,982 | Blood count | 30020 |
| Hematocrit percentage | % | 429,982 | Blood count | 30030 |
| Mean corpuscular volume | femtolitres | 429,980 | Blood count | 30040 |
| Mean corpuscular hemoglobin | picograms | 429,979 | Blood count | 30050 |
| Mean corpuscular hemoglobin concentration | grams/decilitre | 429,976 | Blood count | 30060 |
| Red blood cell (erythrocyte) distribution width | % | 429,980 | Blood count | 30070 |
| Nucleated red blood cell count | 10^9 cells/Litre | 429,195 | Blood count | 30170 |
| Nucleated red blood cell percentage | % | 429,192 | Blood count | 30230 |
| Reticulocyte percentage | % | 422,735 | Blood count | 30240 |
| Reticulocyte count | 10^12 cells/Litre | 422,735 | Blood count | 30250 |
| Mean reticulocyte volume | femtolitres | 422,735 | Blood count | 30260 |
| Mean sphered cell volume | femtolitres | 422,736 | Blood count | 30270 |
| Immature reticulocyte fraction | ratio | 422,735 | Blood count | 30280 |
| High light scatter reticulocyte percentage | % | 422,736 | Blood count | 30290 |
| High light scatter reticulocyte percentage count | 10^12 cells/Litre | 422,735 | Blood count | 30300 |
| **White blood cell** |  |  |  |  |
| White blood cell (leukocyte) count | 10^9 cells/Litre | 429,977 | Blood count | 30000 |
| Lymphocyte count | 10^9 cells/Litre | 429,204 | Blood count | 30120 |
| Monocyte count | 10^9 cells/Litre. | 429,204 | Blood count | 30130 |
| Neutrophil count | 10^9 cells/Litre | 429,204 | Blood count | 30140 |
| Eosinophil count | 10^9 cells/Litre | 429,204 | Blood count | 30150 |
| Basophil count | 10^9 cells/Litre | 429,204 | Blood count | 30160 |
| Lymphocyte percentage | % | 429,210 | Blood count | 30180 |
| Monocyte percentage | % | 429,210 | Blood count | 30190 |
| Neutrophil percentage | % | 429,210 | Blood count | 30200 |
| Eosinophil percentage | % | 429,210 | Blood count | 30210 |
| Basophil percentage | % | 429,210 | Blood count | 30220 |
| **Platelet** |  |  |  |  |
| Platelet count | 10^9 cells/Litre | 429,979 | Blood count | 30080 |
| Platelet crit | 10^9 cells/Litre | 429,976 | Blood count | 30090 |
| Mean platelet (thrombocyte) volume | femtolitres | 429,975 | Blood count | 30100 |
| Platelet distribution width | % | 429,975 | Blood count | 30110 |

**Table C. Measures of covariates at baseline in the UK Biobank.**

| **Variable** | **Filed ID** | **Question or description** | **Categories from raw data** | **Categories for the current study** |
| --- | --- | --- | --- | --- |
| Education | 6138 | Qualifications | College or University degree;  A levels/AS levels or equivalent;  O levels/GCSEs or equivalent;  CSEs or equivalent;  NVQ or HND or HNC or equivalent;  Other professional qualifications eg: nursing, teaching. | 0 = College or University degree;  1 = Others (Below College or University degree). |
| Employment | 6142 | Current employment status | In paid employment or self-employed;  Retired;  Looking after home and/or family;  Unable to work because of sickness or disability;  Unemployed;  Doing unpaid or voluntary work;  Full or part-time student;  None of the above. | 0 = Employed (including those paid employment or self-employed, retired, doing unpaid or voluntary work, or being full or part time students);  1 = Unemployed. |
| Townsend deprivation index | 22189 | Townsend deprivation index at recruitment | Continuous. | Continuous (values closer to 0 indicate greater deprivation). |
| Smoking | 20116 | The current/past smoking status of the participant. | Never;  Past;  Current. | 0 = Never or past smoker;  1 = Current smoker |
| Alcohol consumption | 1558 | About how often do you drink alcohol? | Never;  Special occasions only;  One to three times a month;  Once or twice a week;  Three or four times a week;  Daily or almost daily. | 0 = < 3 times/week;  1 = ≥ 3 times/week. |
| Body mass index | 21001 | Body mass index (BMI) | kg/m^2^ | 0 = < 25 kg/m^2^;  1 = ≥ 25 kg/m^2^. |
| Psychiatric disorders | 130836-130990 | First occurrences (ICD 10: F00-F99) | Date of first record less than date of baseline recruitment. | 1 = Yes;  0 = No. |
| Cardiovascular disease | 131296-131306;  131354;  131360-131368 | First occurrences (ICD 10: I20-I25, I50, and I60-I64 ) | Date of first record less than date of baseline recruitment. | 1 = Yes;  0 = No |
| Cancer | 40005 | Date of cancer diagnosis | Date of first record less than date of baseline recruitment. | 1 = Yes;  0 = No |

**Table D. Summary of missing data for covariates**

| Covariates | Total samples | Missing number | Percentage (%) |
| --- | --- | --- | --- |
| Sex | 449,751 | 0 | 0 |
| Age | 449,751 | 0 | 0 |
| Education | 449,751 | 3,875 | 0.87 |
| Employment | 449,751 | 1,330 | 0.30 |
| Townsend deprivation index | 449,751 | 547 | 0.12 |
| Smoking status | 449,751 | 1,364 | 0.30 |
| Drinking frequency | 449,751 | 251 | 0.06 |
| Body mass index | 449,751 | 0 | 0 |
| Cardiovascular diseases | 449,751 | 0 | 0 |
| Cancer | 449,751 | 2 | 0.0001 |
| Psychiatric disorders | 449,751 | 0 | 0 |

**Table E**. **Detailed information on selected genetic variants and their associations with the exposure and outcome**

| SNP | Effect allele (Exposure) | Other allele (Exposure) | Beta (Exposure) | SE (Exposure) | P-value (Exposure) | Effect allele (Outcome) | Other allele (Outcome) | Beta (Outcome) | SE (Outcome) | P-value (Outcome) |
| --- | --- | --- | --- | --- | --- | --- | --- | --- | --- | --- |
| rs10053447 | T | C | 0.012 | 0.002 | 3.10E-11 | T | C | 0.015 | 0.016 | 0.328 |
| rs1048027 | T | C | 0.011 | 0.002 | 3.80E-08 | T | C | 0.018 | 0.011 | 0.119 |
| rs10828258 | A | G | 0.014 | 0.002 | 7.50E-13 | A | G | 0.001 | 0.009 | 0.918 |
| rs11130207 | T | G | 0.015 | 0.002 | 5.30E-10 | T | G | 0.054 | 0.013 | 0.000 |
| rs11150602 | A | G | 0.011 | 0.002 | 2.00E-08 | A | G | 0.003 | 0.01 | 0.803 |
| rs11660938 | G | T | 0.011 | 0.002 | 1.50E-08 | G | T | 0.036 | 0.009 | 0.000 |
| rs11689546 | A | G | 0.012 | 0.002 | 2.90E-10 | A | G | 0.007 | 0.01 | 0.465 |
| rs12601919 | A | G | 0.013 | 0.002 | 3.20E-08 | A | G | 0.048 | 0.011 | 0.000 |
| rs12712072 | A | G | 0.015 | 0.002 | 1.60E-11 | A | G | 0.025 | 0.01 | 0.016 |
| rs13107325 | C | T | 0.031 | 0.003 | 7.60E-19 | C | T | 0.027 | 0.017 | 0.122 |
| rs1421085 | T | C | 0.013 | 0.002 | 3.00E-12 | T | C | 0.018 | 0.009 | 0.040 |
| rs17707300 | T | C | 0.012 | 0.002 | 2.50E-10 | T | C | 0.016 | 0.009 | 0.073 |
| rs17716502 | C | T | 0.013 | 0.002 | 9.00E-09 | C | T | 0.009 | 0.011 | 0.395 |
| rs2287234 | C | A | 0.011 | 0.002 | 1.10E-08 | C | A | 0.012 | 0.009 | 0.173 |
| rs2847308 | C | T | 0.012 | 0.002 | 5.90E-10 | C | T | 0.036 | 0.009 | 0.000 |
| rs28509789 | T | G | 0.012 | 0.002 | 3.30E-08 | T | G | 0.009 | 0.011 | 0.390 |
| rs303762 | T | C | 0.011 | 0.002 | 3.80E-08 | T | C | 0.023 | 0.009 | 0.013 |
| rs362307 | C | T | 0.029 | 0.003 | 1.50E-16 | C | T | 0.048 | 0.017 | 0.006 |
| rs3821269 | A | G | 0.011 | 0.002 | 2.50E-09 | A | G | 0.012 | 0.009 | 0.180 |
| rs4457304 | A | C | 0.01 | 0.002 | 2.00E-08 | A | C | 0.005 | 0.009 | 0.540 |
| rs4549685 | C | T | 0.011 | 0.002 | 1.80E-08 | C | T | 0.013 | 0.009 | 0.166 |
| rs62082234 | T | G | 0.011 | 0.002 | 4.10E-08 | T | G | 0.017 | 0.009 | 0.064 |
| rs62444907 | C | T | 0.019 | 0.003 | 3.10E-13 | C | T | 0.023 | 0.012 | 0.050 |
| rs6751993 | A | G | 0.015 | 0.002 | 2.50E-09 | A | G | 0.023 | 0.013 | 0.079 |
| rs724701 | A | G | 0.012 | 0.002 | 1.00E-08 | A | G | 0.012 | 0.01 | 0.233 |
| rs72709800 | G | A | 0.021 | 0.003 | 8.80E-10 | G | A | 0.004 | 0.016 | 0.800 |
| rs7703746 | G | A | 0.013 | 0.002 | 6.40E-13 | G | A | 0.002 | 0.009 | 0.779 |
| rs80032406 | T | C | 0.013 | 0.002 | 1.30E-09 | T | C | 0.016 | 0.01 | 0.098 |
| rs8044920 | C | T | 0.011 | 0.002 | 1.60E-08 | C | T | 0.013 | 0.009 | 0.161 |
| rs9953231 | G | A | 0.012 | 0.002 | 3.00E-08 | G | A | 0.023 | 0.01 | 0.024 |

*Note*: *P*-values were estimated using linear mixed models for the exposure GWAS and an inverse variance-weighted fixed-effects meta-analysis for the outcome GWAS. Abbreviations: SNP, single nucleotide polymorphism; SE, standard error; GWAS, Genome-wide association study.

**Table F. List of the 17 comorbidities, assigned weights and associated ICD-10 codes for construction of the Charlson Comorbidity Index**

| Num | Diseases | CCI Score |
| --- | --- | --- |
| 1 | Myocardial infarction | 1 |
| 2 | Congestive heart failure | 1 |
| 3 | Peripheral vascular disease | 1 |
| 4 | Cerebrovascular disease | 1 |
| 5 | Dementia | 1 |
| 6 | Chronic pulmonary disease | 1 |
| 7 | Rheumatic disease | 1 |
| 8 | Peptic ulcer disease | 1 |
| 9 | Diabetes without chronic complication | 1 |
| 10 | Diabetes with chronic complication | 2 |
| 11 | Moderate or severe liver disease | 3 |
| 12 | Renal disease | 2 |
| 13 | Hemiplegia or paraplegia | 2 |
| 14 | Any malignancy, including lymphoma and leukemia, except malignant neoplasm of skin | 2 |
| 15 | Metastatic solid tumor | 6 |
| 16 | Mild liver disease | 1 |
| 17 | AIDS HIV | 6 |

**Table G. Baseline characteristics according to suicide attempt status**

| **Characteristics** | **Overall** | **No** | **Yes** | ***P* value** |
| --- | --- | --- | --- | --- |
|  | **(*N* = 442,920)** | **(*N* = 441,402)** | **(*N* = 1,518)** |  |
| Age, mean (SD) | 56.5 (8.1) | 56.5 (8.1) | 53.7 (8.5) | <0.001 |
| Female, n (%) | 239,686 (54.1) | 238,867 (54.1) | 819 (54.0) | 0.919 |
| College or university degree, n (%) | 147,969 (33.4) | 147,610 (33.4) | 359 (23.6) | <0.001 |
| Employed^a^, n (%) | 409,567 (92.5) | 408,415 (92.5) | 1152 (75.9) | <0.001 |
| Townsend deprivation index, median (IQR) | -2.2 (-3.7, 0.4) | -2.2 (-3.7, 0.3) | -1.1 (-3.1, 2.5) | <0.001 |
| Drinking alcohol ≥ 3 times/week, n (%) | 197,052 (44.5) | 196,462 (44.5) | 590 (38.9) | <0.001 |
| Current smokers, n (%) | 44,973 (10.2) | 44,580 (10.1) | 393 (25.9) | <0.001 |
| Overweight or obesity^b^, n (%) | 295,013 (66.6) | 293,971 (66.6) | 1,042 (68.6) | 0.097 |
| Medical histories, n (%) |  |  |  |  |
| Psychiatric disorders | 73,022 (16.5) | 72,214 (16.4) | 808 (53.2) | <0.001 |
| CVD | 28,942 (6.5) | 28,800 (6.5) | 142 (9.4) | <0.001 |
| Cancer | 40,078 (9.0) | 39,939 (9.0) | 139 (9.2) | 0.918 |
| Frailty status, n (%) |  |  |  | <0.001 |
| Non-frail | 258,139 (58.3) | 257,569 (58.4) | 570 (37.5) |  |
| Pre-frail | 169,087 (38.2) | 168,305 (38.1) | 782 (51.5) |  |
| Frail | 15,694 (3.5) | 15,528 (3.5) | 166 (10.9) |  |
| Frailty components, n (%) |  |  |  |  |
| Weight loss | 67,567 (15.3) | 67,221 (15.2) | 346 (22.8) | <0.001 |
| Exhaustion | 53,643 (12.1) | 53,196 (12.1) | 447 (29.4) | <0.001 |
| Low physical activity | 44,416 (10.0) | 44,136 (10.0) | 280 (18.4) | <0.001 |
| Slow walking pace | 33,299 (7.5) | 33,049 (7.5) | 250 (16.5) | <0.001 |
| Low grip strength | 60,737 (13.7) | 60,460 (13.7) | 277 (18.2) | <0.001 |

Note: The significance of differences between groups were assessed using t tests for normally distributed continuous variables, the Kruskal-Wallis rank-sum test for non-normally distributed continuous variables, and the chi-square test for categorical variables. *P*-values were calculated based on these respective tests.

Abbreviations: BMI, body mass index; CVD, cardiovascular diseases; SD, standard deviation; IQR, interquartile range. ^a^Employed included those in paid employment or self-employed, retired, doing unpaid or voluntary work, or being full or part time students. ^b^BMI ≥ 25 kg/m^2^.

# Table H. Risk of suicide attempt according to frailty status within genetic risk categories

|  | **Genetic risk [HR (95% CI)]** | | | ***P* value for interaction^a^** |
| --- | --- | --- | --- | --- |
| **Frailty status** | **Low** | **Intermediate** | **High** |  |
| Frail | 1(Reference) | 1(Reference) | 1(Reference) | 0.198 |
| Pre-frail | 0.54 (0.37, 0.78) | 0.77 (0.56, 1.05) | 0.81 (0.62, 1.05) |  |
| Non-frail | 0.39 (0.26, 0.58) | 0.46 (0.33, 0.64) | 0.46 (0.35, 0.61) |  |

Abbreviations: CI, confidence interval; HR, hazard ratio.

Models were adjusted for age, sex, education, employment, Townsend Deprivation Index, drinking frequency, smoking status, body mass index, pre-existing psychiatric disorders, cardiovascular diseases, cancer, and the first 10 principal components of ancestry.

^a^*P* value for the multiplicative interaction between frailty status and genetic risk categories were calculated using the likelihood ratio test.

**Table I.** **RERI and AP for additive interaction between frailty status and genetic risk**

|  | **Genetic risk^a^** | | | |
| --- | --- | --- | --- | --- |
|  | Intermediate | | High | |
| **Frailty status** | RERI^b^ (95% CI) | AP^b^ (95% CI) | RERI^b^ (95% CI) | AP^b^ (95% CI) |
| Frailty scores ≥ 1 | 0.356 (-0.029, 0.740) | 0.165 (-0.012, 0.343) | 0.515 (0.113, 0.917) | 0.195 (0.045, 0.345) |

Abbreviation: AP, attributable proportion due to interaction; RERI, relative excess risk due to interaction.

Adjusted for age, sex, education, employment, Townsend Deprivation Index, drinking frequency, smoking status, body mass index, pre-existing psychiatric disorders, cardiovascular diseases, cancer, and the first 10 principal components of ancestry.

^a^Defined by polygenic risk score: low (lowest tertiles), intermediate (second tertiles), and high (highest tertiles) genetic predisposition.

^c^To estimate RERI and AP, the non-frailty (frailty scores = 0) and the low genetic risk groups were the reference categories.

An additive interaction effect was deemed significant if the 95% CIs for both the RERI and AP did not include 0.

**Table J. Multivariable-adjusted Cox regression models for the association of blood biomarkers with suicide attempt**

| **Blood Biomarkers** | **HR^a^ (95% CI)** | ***P* value** | ***FDR*** |
| --- | --- | --- | --- |
| **Liver function** |  |  |  |
| Albumin | 0.9657 (0.9154, 1.0188) | 2.02E-01 | 3.51E-01 |
| Alanine aminotransferase | 1.0148 (0.9672, 1.0648) | 5.49E-01 | 6.70E-01 |
| **Aspartate aminotransferase** | 1.0430 (1.0114, 1.0756) | 7.33E-03 | **2.24E-02** |
| Direct bilirubin | 1.0094 (0.9574, 1.064) | 7.28E-01 | 7.99E-01 |
| **Gamma glutamyltransferase** | 1.0670 (1.0341, 1.1010) | 5.03E-05 | **3.84E-04** |
| **Total bilirubin** | 0.9199 (0.8642, 0.9791) | 8.76E-03 | **2.54E-02** |
| **Total protein** | 0.9078 (0.8599, 0.9584) | 4.69E-04 | **2.60E-03** |
| **Renal function** |  |  |  |
| Urea | 0.9764 (0.9259, 1.0296) | 3.77E-01 | 5.15E-01 |
| Creatinine | 0.9591 (0.8971, 1.0254) | 2.20E-01 | 3.63E-01 |
| Cystatin C | 1.0206 (0.9761, 1.0672) | 3.69E-01 | 5.15E-01 |
| Phosphate | 1.0002 (0.9481, 1.0552) | 9.93E-01 | 9.93E-01 |
| Urate | 0.9673 (0.9092, 1.0292) | 2.94E-01 | 4.48E-01 |
| **Immunometabolic** |  |  |  |
| **Apolipoprotein A** | 1.0995 (1.0348, 1.1683) | 2.16E-03 | **8.24E-03** |
| Apolipoprotein B | 1.0062 (0.9554, 1.0597) | 8.15E-01 | 8.42E-01 |
| Cholesterol | 1.0230 (0.9704, 1.0784) | 3.98E-01 | 5.28E-01 |
| **Glucose** | 1.0684 (1.0282, 1.1102) | 7.24E-04 | **3.15E-03** |
| Glycated hemoglobin (HbA1c) | 1.0430 (0.9993, 1.0885) | 5.39E-02 | 1.22E-01 |
| **HDL cholesterol** | 1.0968 (1.0301, 1.1679) | 3.89E-03 | **1.32E-02** |
| LDL direct | 0.9872 (0.9366, 1.040) | 6.30E-01 | 7.25E-01 |
| Lipoprotein A | 0.9738 (0.9177, 1.0333) | 3.80E-01 | 5.15E-01 |
| C-reactive protein | 1.0365 (0.9937, 1.0811) | 9.58E-02 | 1.95E-01 |
| Triglycerides | 1.0438 (0.9954, 1.0946) | 7.67E-02 | 1.61E-01 |
| **Endocrine** |  |  |  |
| IGF-1 | 0.9443 (0.8958, 0.9955) | 3.34E-02 | 8.86E-02 |
| Oestradiol | 1.0177 (0.9165, 1.1300) | 7.43E-01 | 7.99E-01 |
| SHBG | 1.0366 (0.9778, 1.0990) | 2.28E-01 | 3.65E-01 |
| Testosterone | 1.0879 (0.9692, 1.2211) | 1.53E-01 | 2.75E-01 |
| **Bone and joint** |  |  |  |
| Alkaline phosphatase | 1.0205 (0.9709, 1.0726) | 4.25E-01 | 5.40E-01 |
| Calcium | 0.9891 (0.9377, 1.0433) | 6.87E-01 | 7.77E-01 |
| Rheumatoid factor | 1.0237 (0.8678, 1.2078) | 7.81E-01 | 8.21E-01 |
| **Vitamin D** | 1.0966 (1.0410, 1.1553) | 5.18E-04 | **2.63E-03** |
| **Red blood cell** |  |  |  |
| **Red blood cell (erythrocyte) count** | 0.8460 (0.7994, 0.8954) | 7.56E-09 | **4.61E-07** |
| **Hemoglobin concentration** | 0.8953 (0.8434, 0.9504) | 2.82E-04 | **1.91E-03** |
| **Hematocrit percentage** | 0.9125 (0.8600, 0.9681) | 2.43E-03 | **8.72E-03** |
| **Mean corpuscular volume** | 1.1333 (1.0779, 1.1916) | 9.97E-07 | **1.01E-05** |
| **Mean corpuscular hemoglobin** | 1.0829 (1.0344, 1.1336) | 6.52E-04 | **3.06E-03** |
| Mean corpuscular hemoglobin concentration | 0.9561 (0.9043, 1.0109) | 1.14E-01 | 2.17E-01 |
| Red blood cell (erythrocyte) distribution width | 1.0455 (1.0018, 1.0912) | 4.12E-02 | 1.05E-01 |
| Nucleated red blood cell count | 1.0119 (0.9754, 1.0497) | 5.29E-01 | 6.59E-01 |
| Nucleated red blood cell percentage | 1.0129 (0.9659, 1.0621) | 5.98E-01 | 7.01E-01 |
| Reticulocyte percentage | 1.0262 (1.0000, 1.0531) | 4.97E-02 | 1.17E-01 |
| Reticulocyte count | 1.0243 (0.9867, 1.0634) | 2.09E-01 | 3.54E-01 |
| **Mean reticulocyte volume** | 1.1159 (1.0613, 1.1733) | 1.83E-05 | **1.59E-04** |
| **Mean sphered cell volume** | 1.1357 (1.0816, 1.1926) | 3.26E-07 | **4.97E-06** |
| **Immature reticulocyte fraction** | 1.0997 (1.0442, 1.1582) | 3.21E-04 | **1.96E-03** |
| High light scatter reticulocyte percentage | 1.0110 (0.9972, 1.0250) | 1.20E-01 | 2.22E-01 |
| High light scatter reticulocyte percentage count | 1.0467 (1.0071, 1.0879) | 2.02E-02 | 5.60E-02 |
| **White blood cell** |  |  |  |
| **White blood cell (leukocyte) count** | 1.0501 (1.0201, 1.0810) | 9.63E-04 | **3.92E-03** |
| Lymphocyte count | 0.9128 (0.8354, 0.9974) | 4.36E-02 | 1.06E-01 |
| Monocyte count | 1.0187 (0.9843, 1.0543) | 2.90E-01 | 4.48E-01 |
| **Neutrophil count** | 1.1181 (1.0696, 1.1689) | 8.14E-07 | **9.93E-06** |
| Eosinophil count | 1.0079 (0.9609, 1.0573) | 7.46E-01 | 7.99E-01 |
| Basophil count | 1.0129 (0.9701, 1.0576) | 5.61E-01 | 6.70E-01 |
| **Lymphocyte percentage** | 0.8612 (0.8166, 0.9083) | 3.72E-08 | **1.13E-06** |
| Monocyte percentage | 0.9773 (0.9251, 1.0325) | 4.13E-01 | 5.36E-01 |
| **Neutrophil percentage** | 1.1471 (1.0890, 1.2084) | 2.35E-07 | **4.78E-06** |
| Eosinophil percentage | 0.9723 (0.9215, 1.0258) | 3.04E-01 | 4.52E-01 |
| Basophil percentage | 1.0194 (0.9769, 1.0638) | 3.75E-01 | 5.15E-01 |
| **Platelet** |  |  |  |
| **Platelet count** | 1.0689 (1.0182, 1.1221) | 7.16E-03 | **2.24E-02** |
| Platelet crit | 1.0491 (0.9977, 1.1032) | 6.17E-02 | 1.34E-01 |
| Mean platelet (thrombocyte) volume | 0.9583 (0.9101, 1.0091) | 1.06E-01 | 2.09E-01 |
| Platelet distribution width | 1.0052 (0.9551, 1.0579) | 8.43E-01 | 8.57E-01 |

^a^Models were adjusted for age, sex, education, employment, Townsend Deprivation Index, drinking frequency, smoking status, body mass index, genetic risk, pre-existing psychiatric disorders, cardiovascular diseases, cancer, and frailty scores. Biomarkers were standardized by z-score before analyses.

Bolded indicated that statistical significance. *P*-values were estimated using Cox proportional hazard models.

Abbreviations: SA, suicide attempt; FDR, false discovery rate; CI, confidence interval; HR, hazard ratio.

**Table K. Multivariable-adjusted linear regression models for the associations between frailty scores and suicide attempt-related blood biomarkers**

| **Selected biomarkers** | **Beta^a^ (95% *CI*)** | ***P* value** | ***FDR*** |
| --- | --- | --- | --- |
| **Liver function** |  |  |  |
| Aspartate aminotransferase (L3) | 0.0031 (-0.0006, 0.0069) | 1.03E-01 | 1.03E-01 |
| Gamma glutamyltransferase (L5) | 0.0760 (0.0723, 0.0798) | 2.00E-16 | **2.33E-16** |
| Total bilirubin (L6) | -0.0331 (-0.0368, -0.0293) | 2.00E-16 | **2.33E-16** |
| Total protein (F5) | -0.0065 (-0.0105, -0.0025) | 1.40E-03 | **1.47E-03** |
| **Immunometabolic** |  |  |  |
| Apolipoprotein A (I1) | -0.0786 (-0.0821, -0.0752) | 2.00E-16 | **2.33E-16** |
| Glucose (I4) | 0.0857 (0.0818, 0.0897) | 2.00E-16 | **2.33E-16** |
| HDL cholesterol (I6) | -0.0822 (-0.0855, -0.0788) | 2.00E-16 | **2.33E-16** |
| **Bone and joint** |  |  |  |
| Vitamin D (B4) | -0.1056 (-0.1094, -0.1017) | 2.00E-16 | **2.33E-16** |
| **Red blood cell** |  |  |  |
| Red blood cell count (R1) | -0.0231 (-0.0263, -0.0198) | 2.00E-16 | **2.33E-16** |
| Hemoglobin concentration (R2) | -0.0511 (-0.0541, -0.0481) | 2.00E-16 | **2.33E-16** |
| Hematocrit percentage (R3) | -0.0405 (-0.0436, -0.0374) | 2.00E-16 | **2.33E-16** |
| Mean corpuscular volume (R4) | -0.0232 (-0.0269, -0.0195) | 2.00E-16 | **2.33E-16** |
| Mean corpuscular haemoglobin (R5) | -0.0349 (-0.0386, -0.0312) | 2.00E-16 | **2.33E-16** |
| Mean reticulocyte volume (R12) | 0.0377 (0.0339, 0.0415) | 2.00E-16 | **2.33E-16** |
| Mean sphered cell volume (R13) | 0.0069 (0.0032, 0.0107) | 2.86E-04 | **3.16E-04** |
| Immature reticulocyte fraction (R14) | 0.0730 (0.0693, 0.0767) | 2.00E-16 | **2.33E-16** |
| **White blood cell** |  |  |  |
| White blood cell count (W1) | 0.0780 (0.0743, 0.0817) | 2.00E-16 | **2.33E-16** |
| Neutrophil count (W4) | 0.0884 (0.0847, 0.0921) | 2.00E-16 | **2.33E-16** |
| Lymphocyte percentage (W7) | -0.0418 (-0.0456, -0.0380) | 2.00E-16 | **2.33E-16** |
| Neutrophil percentage (W9) | 0.0426 (0.0387, 0.0464) | 2.00E-16 | **2.33E-16** |
| **Platelet** |  |  |  |
| Platelet count (P1) | 0.0381 (0.0344, 0.0418) | 2.00E-16 | **2.33E-16** |

^a^Models were adjusted for age, sex, education, employment, Townsend Deprivation Index, drinking frequency, smoking status, body mass index, genetic risk, pre-existing psychiatric disorders, cardiovascular diseases, and cancer.

Biomarkers were standardized by z-score before analyses.

Bolded indicated that statistical significance. *P*-values were estimated using linear regression models.

Abbreviations: FDR, false discovery rate; CI, confidence interval; HR, hazard ratio.

**Table L. Two-step Mendelian randomization analyses evaluating the potential causal roles of candidate biomarkers in the association between physical frailty and suicide attempt**

| **Exposures** | **Outcome** | **Methods** | **Number of SNPs** | **Beta** | **SE** | ***P*** | **OR** | **CI_lower** | **CI_upper** |
| --- | --- | --- | --- | --- | --- | --- | --- | --- | --- |
| Frailty | GGT | IVW | 6 | 0.165 | 0.333 | 0.619 | 1.180 | 0.615 | 2.264 |
| Frailty | GGT | MR Egger | 6 | -0.699 | 0.853 | 0.458 | 0.497 | 0.093 | 2.644 |
| Frailty | GGT | Weighted median | 6 | 0.401 | 0.138 | 0.004 | 1.493 | 1.139 | 1.958 |
| Frailty | GGT | IVW (multiplicative random effects) | 6 | 0.165 | 0.333 | 0.619 | 1.180 | 0.615 | 2.264 |
| GGT | SA | IVW | 205 | 0.043 | 0.028 | 0.118 | 1.044 | 0.989 | 1.102 |
| GGT | SA | MR Egger | 205 | 0.031 | 0.049 | 0.532 | 1.031 | 0.937 | 1.135 |
| GGT | SA | Weighted median | 205 | 0.060 | 0.039 | 0.125 | 1.061 | 0.984 | 1.145 |
| GGT | SA | IVW (multiplicative random effects) | 205 | 0.043 | 0.028 | 0.118 | 1.044 | 0.989 | 1.102 |
| Frailty | TBIL | IVW | 6 | -0.412 | 0.233 | 0.077 | 0.663 | 0.420 | 1.046 |
| Frailty | TBIL | MR Egger | 6 | 0.020 | 0.640 | 0.976 | 1.021 | 0.291 | 3.579 |
| Frailty | TBIL | Weighted median | 6 | -0.187 | 0.121 | 0.121 | 0.829 | 0.655 | 1.050 |
| Frailty | TBIL | IVW (multiplicative random effects) | 6 | -0.412 | 0.233 | 0.077 | 0.663 | 0.420 | 1.046 |
| TBIL | SA | IVW | 100 | 0.005 | 0.036 | 0.893 | 1.005 | 0.937 | 1.078 |
| TBIL | SA | MR Egger | 100 | 0.016 | 0.049 | 0.749 | 1.016 | 0.922 | 1.119 |
| TBIL | SA | Weighted median | 100 | 0.020 | 0.049 | 0.675 | 1.021 | 0.928 | 1.122 |
| TBIL | SA | IVW (multiplicative random effects) | 100 | 0.005 | 0.036 | 0.893 | 1.005 | 0.937 | 1.078 |
| Frailty | TP | IVW | 6 | 0.093 | 0.279 | 0.740 | 1.097 | 0.634 | 1.897 |
| Frailty | TP | MR Egger | 6 | -0.460 | 0.757 | 0.577 | 0.632 | 0.143 | 2.787 |
| Frailty | TP | Weighted median | 6 | -0.199 | 0.168 | 0.236 | 0.820 | 0.590 | 1.139 |
| Frailty | TP | IVW (multiplicative random effects) | 6 | 0.093 | 0.279 | 0.740 | 1.097 | 0.634 | 1.897 |
| TP | SA | IVW | 181 | 0.106 | 0.037 | 0.004 | 1.112 | 1.034 | 1.196 |
| TP | SA | MR Egger | 181 | 0.260 | 0.080 | 0.001 | 1.296 | 1.109 | 1.516 |
| TP | SA | Weighted median | 181 | 0.200 | 0.051 | 0.000 | 1.222 | 1.106 | 1.350 |
| TP | SA | IVW (multiplicative random effects) | 181 | 0.106 | 0.037 | 0.004 | 1.112 | 1.034 | 1.196 |
| Frailty | Glucose | IVW | 6 | 0.519 | 0.149 | 0.001 | 1.680 | 1.254 | 2.251 |
| Frailty | Glucose | MR Egger | 6 | 0.020 | 0.341 | 0.955 | 1.020 | 0.523 | 1.990 |
| Frailty | Glucose | Weighted median | 6 | 0.485 | 0.130 | 0.000 | 1.624 | 1.260 | 2.094 |
| Frailty | Glucose | IVW (multiplicative random effects) | 6 | 0.519 | 0.149 | 0.001 | 1.680 | 1.254 | 2.251 |
| Glucose | SA | IVW | 79 | -0.039 | 0.046 | 0.392 | 0.962 | 0.879 | 1.052 |
| Glucose | SA | MR Egger | 79 | 0.012 | 0.081 | 0.880 | 1.012 | 0.864 | 1.186 |
| Glucose | SA | Weighted median | 79 | -0.056 | 0.067 | 0.403 | 0.945 | 0.829 | 1.078 |
| Glucose | SA | IVW (multiplicative random effects) | 79 | -0.039 | 0.046 | 0.392 | 0.962 | 0.879 | 1.052 |
| Frailty | RBC | IVW | 31 | -0.048 | 0.133 | 0.717 | 0.953 | 0.735 | 1.236 |
| Frailty | RBC | MR Egger | 31 | 0.451 | 0.531 | 0.403 | 1.569 | 0.554 | 4.444 |
| Frailty | RBC | Weighted median | 31 | 0.085 | 0.066 | 0.199 | 1.088 | 0.957 | 1.239 |
| Frailty | RBC | IVW (multiplicative random effects) | 31 | -0.048 | 0.133 | 0.717 | 0.953 | 0.735 | 1.236 |
| RBC | SA | IVW | 371 | -0.049 | 0.025 | 0.047 | 0.952 | 0.907 | 0.999 |
| RBC | SA | MR Egger | 371 | 0.025 | 0.050 | 0.619 | 1.025 | 0.930 | 1.130 |
| RBC | SA | Weighted median | 371 | -0.038 | 0.035 | 0.281 | 0.963 | 0.898 | 1.032 |
| RBC | SA | IVW (multiplicative random effects) | 371 | -0.049 | 0.025 | 0.047 | 0.952 | 0.907 | 0.999 |
| Frailty | Hemoglobin | IVW | 31 | 0.044 | 0.118 | 0.710 | 1.045 | 0.829 | 1.317 |
| Frailty | Hemoglobin | MR Egger | 31 | 0.964 | 0.446 | 0.039 | 2.623 | 1.093 | 6.291 |
| Frailty | Hemoglobin | Weighted median | 31 | 0.021 | 0.065 | 0.750 | 1.021 | 0.899 | 1.160 |
| Frailty | Hemoglobin | IVW (multiplicative random effects) | 31 | 0.044 | 0.118 | 0.710 | 1.045 | 0.829 | 1.317 |
| Hemoglobin | SA | IVW | 312 | 0.006 | 0.029 | 0.831 | 1.006 | 0.951 | 1.065 |
| Hemoglobin | SA | MR Egger | 312 | -0.057 | 0.060 | 0.347 | 0.945 | 0.839 | 1.063 |
| Hemoglobin | SA | Weighted median | 312 | 0.013 | 0.042 | 0.752 | 1.013 | 0.933 | 1.101 |
| Hemoglobin | SA | IVW (multiplicative random effects) | 312 | 0.006 | 0.029 | 0.831 | 1.006 | 0.951 | 1.065 |
| Frailty | Hematocrit | IVW | 31 | 0.063 | 0.119 | 0.599 | 1.065 | 0.843 | 1.345 |
| Frailty | Hematocrit | MR Egger | 31 | 0.980 | 0.452 | 0.039 | 2.664 | 1.099 | 6.462 |
| Frailty | Hematocrit | Weighted median | 31 | 0.028 | 0.065 | 0.661 | 1.029 | 0.906 | 1.168 |
| Frailty | Hematocrit | IVW (multiplicative random effects) | 31 | 0.063 | 0.119 | 0.599 | 1.065 | 0.843 | 1.345 |
| Hematocrit | SA | IVW | 297 | -0.044 | 0.027 | 0.103 | 0.957 | 0.908 | 1.009 |
| Hematocrit | SA | MR Egger | 297 | 0.008 | 0.053 | 0.876 | 1.008 | 0.909 | 1.119 |
| Hematocrit | SA | Weighted median | 297 | -0.030 | 0.040 | 0.460 | 0.971 | 0.897 | 1.050 |
| Hematocrit | SA | IVW (multiplicative random effects) | 297 | -0.044 | 0.027 | 0.103 | 0.957 | 0.908 | 1.009 |
| Frailty | MRV | IVW | 31 | 0.182 | 0.123 | 0.137 | 1.200 | 0.944 | 1.526 |
| Frailty | MRV | MR Egger | 31 | 0.509 | 0.494 | 0.312 | 1.663 | 0.631 | 4.381 |
| Frailty | MRV | Weighted median | 31 | 0.156 | 0.067 | 0.020 | 1.168 | 1.025 | 1.332 |
| Frailty | MRV | IVW (multiplicative random effects) | 31 | 0.182 | 0.123 | 0.137 | 1.200 | 0.944 | 1.526 |
| MRV | SA | IVW | 338 | 0.043 | 0.019 | 0.028 | 1.044 | 1.005 | 1.084 |
| MRV | SA | MR Egger | 338 | 0.002 | 0.035 | 0.957 | 1.002 | 0.935 | 1.074 |
| MRV | SA | Weighted median | 338 | 0.038 | 0.030 | 0.208 | 1.038 | 0.979 | 1.101 |
| MRV | SA | IVW (multiplicative random effects) | 338 | 0.043 | 0.019 | 0.028 | 1.044 | 1.005 | 1.084 |
| Frailty | MSCV | IVW | 31 | 0.155 | 0.133 | 0.245 | 1.167 | 0.899 | 1.516 |
| Frailty | MSCV | MR Egger | 31 | 0.482 | 0.538 | 0.377 | 1.620 | 0.564 | 4.649 |
| Frailty | MSCV | Weighted median | 31 | 0.142 | 0.064 | 0.026 | 1.153 | 1.017 | 1.307 |
| Frailty | MSCV | IVW (multiplicative random effects) | 31 | 0.155 | 0.133 | 0.245 | 1.167 | 0.899 | 1.516 |
| MSCV | SA | IVW | 375 | 0.038 | 0.020 | 0.053 | 1.039 | 0.999 | 1.080 |
| MSCV | SA | MR Egger | 375 | -0.036 | 0.035 | 0.309 | 0.965 | 0.901 | 1.034 |
| MSCV | SA | Weighted median | 375 | 0.021 | 0.028 | 0.450 | 1.022 | 0.966 | 1.080 |
| MSCV | SA | IVW (multiplicative random effects) | 375 | 0.038 | 0.020 | 0.053 | 1.039 | 0.999 | 1.080 |
| Frailty | IRF | IVW | 31 | 0.086 | 0.098 | 0.379 | 1.090 | 0.900 | 1.321 |
| Frailty | IRF | MR Egger | 31 | 0.196 | 0.398 | 0.626 | 1.216 | 0.558 | 2.652 |
| Frailty | IRF | Weighted median | 31 | 0.034 | 0.058 | 0.558 | 1.035 | 0.923 | 1.160 |
| Frailty | IRF | IVW (multiplicative random effects) | 31 | 0.086 | 0.098 | 0.379 | 1.090 | 0.900 | 1.321 |
| IRF | SA | IVW | 242 | -0.014 | 0.027 | 0.602 | 0.986 | 0.936 | 1.039 |
| IRF | SA | MR Egger | 242 | -0.027 | 0.048 | 0.573 | 0.973 | 0.885 | 1.070 |
| IRF | SA | Weighted median | 242 | -0.011 | 0.040 | 0.786 | 0.989 | 0.915 | 1.070 |
| IRF | SA | IVW (multiplicative random effects) | 242 | -0.014 | 0.027 | 0.602 | 0.986 | 0.936 | 1.039 |
| Frailty | WBC | IVW | 31 | 0.193 | 0.175 | 0.268 | 1.213 | 0.862 | 1.709 |
| Frailty | WBC | MR Egger | 31 | 0.025 | 0.709 | 0.972 | 1.025 | 0.256 | 4.113 |
| Frailty | WBC | Weighted median | 31 | 0.180 | 0.060 | 0.003 | 1.197 | 1.064 | 1.347 |
| Frailty | WBC | IVW (multiplicative random effects) | 31 | 0.193 | 0.175 | 0.268 | 1.213 | 0.862 | 1.709 |
| WBC | SA | IVW | 346 | 0.015 | 0.028 | 0.608 | 1.015 | 0.960 | 1.073 |
| WBC | SA | MR Egger | 346 | 0.002 | 0.065 | 0.979 | 1.002 | 0.882 | 1.138 |
| WBC | SA | Weighted median | 346 | 0.049 | 0.038 | 0.193 | 1.051 | 0.975 | 1.131 |
| WBC | SA | IVW (multiplicative random effects) | 346 | 0.015 | 0.028 | 0.608 | 1.015 | 0.960 | 1.073 |
| Frailty | Neutrophil count | IVW | 31 | 0.201 | 0.163 | 0.217 | 1.223 | 0.889 | 1.682 |
| Frailty | Neutrophil count | MR Egger | 31 | 0.099 | 0.661 | 0.882 | 1.104 | 0.303 | 4.030 |
| Frailty | Neutrophil count | Weighted median | 31 | 0.224 | 0.057 | 0.000 | 1.251 | 1.120 | 1.399 |
| Frailty | Neutrophil count | IVW (multiplicative random effects) | 31 | 0.201 | 0.163 | 0.217 | 1.223 | 0.889 | 1.682 |
| Neutrophil count | SA | IVW | 307 | -0.046 | 0.032 | 0.156 | 0.955 | 0.897 | 1.018 |
| Neutrophil count | SA | MR Egger | 307 | 0.007 | 0.071 | 0.916 | 1.007 | 0.877 | 1.157 |
| Neutrophil count | SA | Weighted median | 307 | -0.005 | 0.042 | 0.909 | 0.995 | 0.917 | 1.080 |
| Neutrophil count | SA | IVW (multiplicative random effects) | 307 | -0.046 | 0.032 | 0.156 | 0.955 | 0.897 | 1.018 |
| Frailty | Lym% | IVW | 31 | -0.101 | 0.089 | 0.259 | 0.904 | 0.759 | 1.077 |
| Frailty | Lym% | MR Egger | 31 | 0.010 | 0.362 | 0.979 | 1.010 | 0.497 | 2.054 |
| Frailty | Lym% | Weighted median | 31 | -0.055 | 0.056 | 0.327 | 0.946 | 0.848 | 1.057 |
| Frailty | Lym% | IVW (multiplicative random effects) | 31 | -0.101 | 0.089 | 0.259 | 0.904 | 0.759 | 1.077 |
| Lym% | SA | IVW | 271 | 0.010 | 0.030 | 0.746 | 1.010 | 0.953 | 1.070 |
| Lym% | SA | MR Egger | 271 | 0.019 | 0.068 | 0.777 | 1.019 | 0.893 | 1.164 |
| Lym% | SA | Weighted median | 271 | -0.020 | 0.045 | 0.655 | 0.980 | 0.898 | 1.070 |
| Lym% | SA | IVW (multiplicative random effects) | 271 | 0.010 | 0.030 | 0.746 | 1.010 | 0.953 | 1.070 |
| Frailty | Neut% | IVW | 31 | 0.110 | 0.097 | 0.259 | 1.116 | 0.923 | 1.350 |
| Frailty | Neut% | MR Egger | 31 | 0.233 | 0.394 | 0.559 | 1.262 | 0.583 | 2.730 |
| Frailty | Neut% | Weighted median | 31 | 0.114 | 0.059 | 0.053 | 1.121 | 0.998 | 1.258 |
| Frailty | Neut% | IVW (multiplicative random effects) | 31 | 0.110 | 0.097 | 0.259 | 1.116 | 0.923 | 1.350 |
| Neut% | SA | IVW | 279 | 0.015 | 0.033 | 0.648 | 1.015 | 0.951 | 1.083 |
| Neut% | SA | MR Egger | 279 | -0.018 | 0.076 | 0.816 | 0.982 | 0.847 | 1.140 |
| Neut% | SA | Weighted median | 279 | 0.021 | 0.047 | 0.658 | 1.021 | 0.932 | 1.119 |
| Neut% | SA | IVW (multiplicative random effects) | 279 | 0.015 | 0.033 | 0.648 | 1.015 | 0.951 | 1.083 |
| Frailty | Platelet count | IVW | 31 | 0.086 | 0.089 | 0.334 | 1.090 | 0.915 | 1.297 |
| Frailty | Platelet count | MR Egger | 31 | -0.021 | 0.360 | 0.955 | 0.980 | 0.483 | 1.985 |
| Frailty | Platelet count | Weighted median | 31 | 0.068 | 0.063 | 0.275 | 1.071 | 0.947 | 1.211 |
| Frailty | Platelet count | IVW (multiplicative random effects) | 31 | 0.086 | 0.089 | 0.334 | 1.090 | 0.915 | 1.297 |
| Platelet count | SA | IVW | 400 | 0.026 | 0.020 | 0.204 | 1.026 | 0.986 | 1.068 |
| Platelet count | SA | MR Egger | 400 | -0.006 | 0.038 | 0.872 | 0.994 | 0.922 | 1.071 |
| Platelet count | SA | Weighted median | 400 | 0.043 | 0.032 | 0.177 | 1.044 | 0.981 | 1.110 |
| Platelet count | SA | IVW (multiplicative random effects) | 400 | 0.026 | 0.020 | 0.204 | 1.026 | 0.986 | 1.068 |

Note: For both the frailty-biomarkers and biomarkers-SA analyses, *P*-values were estimated using the corresponding Mendelian randomization (MR) methods, including inverse-variance weighted (IVW), MR-Egger, weighted median, and IVW with multiplicative random effects, as applicable. Abbreviations: SA, suicide attempt; CI, confidence interval; OR, odds ratio; GGT, Gamma glutamyltransferase; TBIL, Total bilirubin; TP, Total protein; RBC, Red blood cell; MRV, Mean reticulocyte volume; MSCV, Mean sphered cell volume; IRF, Immature reticulocyte fraction; WBC, White blood cell; Lym%, Lymphocyte percentage; Neut%, Neutrophill percentage.

# Table M. Associations of frailty status with the risk of suicide attempt after excluding the cases occurred within the first two-years of follow-up.

| **Frailty status** | **No. of cases/**  **person-years** | **Model 1 (*N* = 442,641)** | | **Model 2 (*N* = 442,641)** | | **Model 3 (*N* = 442,641)** | |
| --- | --- | --- | --- | --- | --- | --- | --- |
|  |  | **HR (95% CI)** | ***P* value** | **HR (95% CI)** | ***P* value** | **HR (95% CI)** | ***P* value** |
| Non-frail (0) | 476/3,454,479 | 1(Reference) |  | 1(Reference) |  | 1(Reference) |  |
| Pre-frail (1-2) | 635/2,218,747 | 1.83 (1.62, 2.07) | <0.001 | 1.81 (1.60, 2.04) | <0.001 | 1.61 (1.42, 1.82) | <0.001 |
| Frail (3-5) | 128/194,274 | 2.94 (2.39, 3.63) | <0.001 | 2.80 (2.27, 3.47) | <0.001 | 2.14 (1.73, 2.65) | <0.001 |
| *P* for trend |  | <0.001 | | <0.001 | | <0.001 | |
| Per 1-point increase | 1,239/5,867,501 | 1.42 (1.35, 1.50) | <0.001 | 1.40 (1.33, 1.48) | <0.001 | 1.29 (1.22, 1.36) | <0.001 |

Note: *P*-values were estimated using Cox proportional hazard models. Abbreviations: HR, hazards ratio; CI, confidence interval.

Model 1 was adjusted for age, sex, education, employment, Townsend Deprivation Index, and genetic risk.

Model 2 was adjusted for Model 1 plus smoking status, drinking frequency, and body mass index.

Model 3 was adjusted for Model 2 plus pre-existing psychiatric disorders, cardiovascular diseases, and cancer.

# Table N. Associations of frailty status with the risk of suicide attempt after using multiple imputation for missing covariates.

| **Frailty status** | **No. of cases/**  **person-years** | **Model 1 (*N* = 449,751)** | | **Model 2 (*N* = 449,751)** | | **Model 3 (*N* = 449,751)** | |
| --- | --- | --- | --- | --- | --- | --- | --- |
|  |  | **HR (95% CI)** | ***P* value** | **HR (95% CI)** | ***P* value** | **HR (95% CI)** | ***P* value** |
| Non-frail (0) | 586/3,495,735 | 1(Reference) |  | 1(Reference) |  | 1(Reference) |  |
| Pre-frail (1-2) | 804/2,260,285 | 1.87 (1.68, 2.08) | <0.001 | 1.81 (1.62, 2.02) | <0.001 | 1.61 (1.44, 1.80) | <0.001 |
| Frail (3-5) | 172/200,636 | 3.11 (2.59, 3.73) | <0.001 | 2.84 (2.35, 3.42) | <0.001 | 2.18 (1.80, 2.64) | <0.001 |
| *P* for trend |  | <0.001 | | <0.001 | | <0.001 | |
| Per 1-point increase | 1,562/5,867,501 | 1.44 (1.37, 1.51) | <0.001 | 1.41 (1.34, 1.48) | <0.001 | 1.29 (1.23, 1.36) | <0.001 |

Note: *P*-values were estimated using Cox proportional hazard models. Abbreviations: HR, hazards ratio; CI, confidence interval.

Model 1 was adjusted for age, sex, education, employment, and Townsend Deprivation Index.

Model 2 was adjusted for Model 1 plus smoking status, drinking frequency, and body mass index.

Model 3 was adjusted for Model 2 plus pre-existing psychiatric disorders, cardiovascular diseases, and cancer.

# Table O. Associations of frailty status with the risk of suicide attempt after excluding participants with baseline CVD, cancer, psychiatric disorders, or higher deprivation.

| **Frailty status** | **Excluding participants with baseline CVD or cancer or** **psychiatric disorders**  **(*N* = 316,825)** | | **Excluding participants with higher levels of deprivation (TDI > median)**  **(*N* = 221,998)** | |
| --- | --- | --- | --- | --- |
|  | **HR^a^ (95% CI)** | ***P* value** | **HR^b^ (95% CI)** | ***P* value** |
| Non-frail (0) | 1(Reference) |  | 1(Reference) |  |
| Pre-frail (1-2) | 1.71 (1.45, 2.03) | <0.001 | 1.88 (1.58, 2.23) | <0.001 |
| Frail (3-5) | 2.61 (1.80, 3.78) | <0.001 | 2.02 (1.35, 3.04) | <0.001 |
| *P* for trend | <0.001 | | <0.001 | |
| Per 1-point increase | 1.43 (1.31, 1.56) | <0.001 | 1.40 (1.28, 1.53) | <0.001 |

Note: *P*-values were estimated using Cox proportional hazard models. Abbreviations: HR, hazards ratio; CI, confidence interval; TDI, Townsend Deprivation Index.

^a^Models were adjusted for age, sex, education, employment, TDI, genetic risk, smoking status, drinking frequency, and body mass index.

^b^Models were adjusted for age, sex, education, employment, genetic risk, smoking status, drinking frequency, body mass index, pre-existing psychiatric disorders, cardiovascular diseases, and cancer.

# Table P. Associations of frailty status with the risk of suicide attempt after further adjustment for the Charlson Comorbidity Index.

| **Frailty status** | **Model 3 + the Charlson Comorbidity Index (*N* = 442,920)** | |
| --- | --- | --- |
|  | **HR (95% CI)** | ***P* value** |
| Non-frail (0) | 1(Reference) |  |
| Pre-frail (1-2) | 1.60 (1.43, 1.79) | <0.001 |
| Frail (3-5) | 2.11 (1.74, 2.57) | <0.001 |
| *P* for trend | <0.001 | |
| Per 1-point increase | 1.29 (1.22, 1.35) | <0.001 |

Note: *P*-values were estimated using Cox proportional hazard models. Abbreviations: HR, hazards ratio; CI, confidence interval.

Model 3 was adjusted for age, sex, education, employment, Townsend Deprivation Index, genetic risk, smoking status, drinking frequency, body mass index, pre-existing psychiatric disorders, cardiovascular diseases and cancer.

# Table Q. E-values for the associations between frailty status and suicide attempt.

| **Exposure comparison** | **HR (95% CI) in Model 3** | **E-value**  **(point estimate)** | **E-value**  **(lower 95% CI)** |
| --- | --- | --- | --- |
| Pre-frailty vs non-frailty | 1.61 (1.44, 1.80) | 2.60 | 2.24 |
| Frailty vs non-frailty | 2.16 (1.78, 2.61) | 3.74 | 2.96 |

Note: HRs (95% CIs) were estimated using Cox proportional hazard models. Abbreviations: HR, hazards ratio; CI, confidence interval.

Model 3 was adjusted for age, sex, education, employment, Townsend Deprivation Index, smoking status, drinking frequency, body mass index, pre-existing psychiatric disorders, cardiovascular diseases, and cancer. E-values quantify the minimum strength of association that an unmeasured confounder would need to have with both the exposure (frailty status) and the outcome (suicide attempt), beyond the measured covariates, to fully explain away the observed associations.

# Table R. Associations of frailty status with the risk of suicide attempt accounting for death as a competing risk.

| **Frailty status** | **Accounting for death as a competing risk (*N* = 442,920)** | |
| --- | --- | --- |
|  | **sHR (95% CI)** | ***P* value** |
| Non-frail (0) | 1(Reference) |  |
| Pre-frail (1-2) | 1.56 (1.37, 1.77) | <0.001 |
| Frail (3-5) | 2.01 (1.60, 2.52) | <0.001 |
| *P* for trend | <0.001 | |
| Per 1-point increase | 1.25 (1.18, 1.32) | <0.001 |

Note: *P*-values were estimated using Cox proportional hazard models. Models were adjusted for age, sex, education, employment, Townsend Deprivation Index, genetic risk, smoking status, drinking frequency, body mass index, pre-existing psychiatric disorders, cardiovascular diseases and cancer.

Abbreviations: sHR, subdistribution hazards ratio; CI, confidence interval.

**Table S. Stratified analysis for the association of the frailty status with the risk of suicide attempt.**

| **Subgroups** |  |  | **Risk of suicide attempt [HR (95% CI)]** | | | |
| --- | --- | --- | --- | --- | --- | --- |
|  | ***N*** | **Cases** | **Non-frailty** | **Pre-frailty** | **Frailty** | ***P* for interaction** |
| **Age groups** |  |  |  |  |  | 0.212 |
| <65 years | 359422 | 1,301 | Reference | 1.67 (1.48, 1.88) | 2.09 (1.70, 2.57) |  |
| ≥65 years | 83498 | 217 | Reference | 1.39 (1.21, 1.80) | 2.71 (2.70, 4.33) |  |
| **Sex** |  |  |  |  |  | 0.960 |
| Male | 203,234 | 699 | Reference | 1.61 (1.37, 1.90) | 2.20 (1.65, 2.93) |  |
| Female | 239,686 | 819 | Reference | 1.65 (1.41, 1.92) | 2.15 (1.67, 2.77) |  |
| **Education level** |  |  |  |  |  | 0.107 |
| College or university | 147,969 | 359 | Reference | 1.79 (1.44, 2.23) | 3.18 (2.03, 4.98) |  |
| Others | 294,951 | 1,159 | Reference | 1.57 (1.38, 1.78) | 2.00 (1.62, 2.46) |  |
| **Smoking status** |  |  |  |  |  | 0.332 |
| Previous/never | 397,947 | 1,125 | Reference | 1.62 (1.42, 1.83) | 2.30 (1.83, 2.89) |  |
| Current | 44,973 | 393 | Reference | 1.61 (1.28, 2.03) | 1.90 (1.35, 2.68) |  |
| **Drinking frequency** |  |  |  |  |  | 0.710 |
| < 3 times/week | 245,868 | 928 | Reference | 1.60 (1.38, 1.85) | 2.28 (1.82, 2.85) |  |
| ≥3 times/week | 197,052 | 590 | Reference | 1.66 (1.40, 1.97) | 1.97 (1.36, 2.86) |  |
| **Body mass index** |  |  |  |  |  | 0.876 |
| < 25 kg/m^2^ | 147,907 | 476 | Reference | 1.57 (1.30, 1.91) | 1.74 (1.16, 2.63) |  |
| ≥ 25 kg/m^2^ | 295,013 | 1,042 | Reference | 1.65 (1.44, 1.89) | 2.31 (1.86, 2.87) |  |

Models were adjusted for age, sex, education, employment, Townsend Deprivation Index, genetic risk, drinking frequency, smoking status, body mass index, pre-existing psychiatric disorders, cardiovascular diseases, and cancer (excluding stratification factors). *P*-values for interaction were estimated by including the cross-product term between frailty status and subgroup variables in the Cox proportional hazards model.

Abbreviations: CI, confidence interval; HR, hazard ratio.

**Table T**. **Estimates from MR analysis using the IVW method for the association between frailty and suicide attempt, and replicated estimates from MR analyses using the MR-Egger regression and weighted median methods for the same association**

| Exposure | Inverse variance weighted | | | MR Egger | | | Weighted median | | |
| --- | --- | --- | --- | --- | --- | --- | --- | --- | --- |
|  | No. of SNPs | OR (95% CI) | *P* value | No. of SNPs | OR (95% CI) | *P* value | No. of SNPs | OR (95% CI) | *P* value |
| Frailty | 30 | 2.06 (1.21, 3.52) | 8.04E-03 | 30 | 4.46 (0.53, 37.71) | 0.18 | 30 | 2.44 (1.51, 3.95) | 2.77E-04 |

Note: *P*-values were estimated using the corresponding MR methods, including the IVW method, MR-Egger regression, and the weighted median method.

Abbreviations: MR, Mendelian randomization; CI, confidence interval; OR, odds ratio; IVW, inverse-variance weighted; SNP; single nucleotide polymorphisms.

**Table U**. **Replicated estimates from MR analysis using the random-effects IVW method for the association between frailty and suicide attempt, along with MR-Egger intercept test results for horizontal pleiotropy and heterogeneity test results using Cochran’s Q statistic.**

| Exposure | Heterogeneity Test | | | Random-effects IVW Estimates | | | MR-Egger Tests | | |
| --- | --- | --- | --- | --- | --- | --- | --- | --- | --- |
|  | Q | Q_df | Q_pvalue | No. of SNPs | OR (95% CI) | *P* value | Egger_intercept | Std. error | *P* value |
| Frailty | 105.50 | 29 | 1.27E-10 | 30 | 2.06 (1.21, 3.52) | 8.04E-03 | -0.01 | 0.01 | 0.47 |

Note: *P*-values were estimated using the corresponding statistical tests, including Cochran’s Q statistic for heterogeneity, the random-effects IVW method for the main MR analysis, and the MR-Egger intercept test for horizontal pleiotropy. Abbreviations: MR, Mendelian randomization; CI, confidence interval; OR, odds ratio; IVW, inverse-variance weighted; SNP; single nucleotide polymorphisms.

# Table V. Associations between physical frailty and suicidal ideation, and mediating role of suicidal ideation in the frailty-SA association

| **Frailty status** | **Association between frailty and suicidal ideation**^a^ **(*N* = 143,773)** | | **Mediating role of suicidal ideation in the frailty-SA association (*N* = 143,773)** | |
| --- | --- | --- | --- | --- |
|  | **HR (95% CI)** | ***P* value** | **PM (95% CI)** | ***P* value** |
| Non-frail (0) | 1(Reference) |  | 32.8%  (31.3%, 34.1%) | < 0.001 |
| Pre-frail (1-2) | 1.75 (1.65, 1.84) | <0.001 |  |  |
| Frail (3-5) | 3.55 (3.12, 4.03) | <0.001 |  |  |
| *P* for trend | <0.001 | |  |  |
| Per 1-point increase | 1.53 (1.48, 1.58) | <0.001 |  |  |

Note: HRs (95% CIs) for the associations between frailty status and suicidal ideation were estimated using Cox proportional hazards models, and corresponding P-values were derived from these models. The significance of the PM was estimated using a nonparametric bootstrap method with 1,000 resamples, and corresponding P-values were derived from this procedure. ^a^Suicidal ideation was measured based on self-reported data from participants in the online follow-up, with approximately one-third of baseline participants providing relevant information. Models were adjusted for age, sex, education, employment, Townsend Deprivation Index, genetic risk, smoking status, drinking frequency, body mass index, pre-existing psychiatric disorders, cardiovascular diseases, and cancer.

Abbreviations: HR, hazard ratio; CI, confidence interval; SA, suicide attempt; PM, Proportion mediated.

**Table W. Comparison of baseline characteristics between included and excluded populations**

| **Characteristics** | **Included** | **Excluded** | ***P* value** | **SMD** **absolute value** |
| --- | --- | --- | --- | --- |
|  | **(*N* = 442,920)** | **(*N* = 59,381)** |  |  |
| Age, mean (SD) | 56.5 (8.1) | 56.7 (8.3) | <0.001 | 0.024 |
| Female, n (%) | 239,686 (54.1) | 33,569 (56.5) | <0.001 | 0.048 |
| College or university degree, n (%) | 147,969 (33.4) | 13,109 (26.6) | <0.001 | 0.148 |
| Employed, n (%) | 409,567 (92.5) | 48,053 (85.2) | <0.001 | 0.232 |
| Townsend deprivation index, mean (SD) | -1.4 (3.0) | -0.5 (3.4) | <0.001 | 0.281 |
| Drinking alcohol ≥ 3 times/week, n (%) | 197,052 (44.5) | 20,079 (34.7) | <0.001 | 0.200 |
| Current smokers, n (%) | 44,973 (10.2) | 7978 (14.1) | <0.001 | 0.119 |
| Overweight or obesity, n (%) | 295,013 (66.6) | 39,231 (66.1) | 0.009 | 0.011 |
| Medical histories, n (%) |  |  |  |  |
| Psychiatric disorders | 73,022 (16.5) | 12,343 (20.8) | <0.001 | 0.110 |
| CVD | 28,942 (6.5) | 5,477 (9.2) | <0.001 | 0.100 |
| Cancer | 40,078 (9.0) | 5,695 (9.6) | <0.001 | 0.021 |

Note: P-values were estimated using t tests for normally distributed continuous variables, the Wilcoxon rank-sum test for non-normally distributed continuous variables, and the chi-square test for categorical variables. Abbreviations: CVD, cardiovascular diseases; SD, standard deviation; SMD: standardized mean difference.
