# Supplementary material for: Physical frailty, genetic risk, mediating biomarkers, and risk of suicide attempt: A prospective cohort study
Source: PLoS Med. 2026 Apr 6;23(4):e1005045. doi: 10.1371/journal.pmed.1005045 (PMC13065332; doi:10.1371/journal.pmed.1005045)
Supplement: Attachment — Submitted filename: Point-by-Point-Response-R1.docx [file pmed.1005045.s006.docx]

Dear Editor and reviewers:

On behalf of all the contributing authors, I would like to express our sincere appreciation for your letter and the reviewers’ constructive comments and suggestions concerning our article, which helped us to improve the quality of our manuscript (PMEDICINE-D-25-03230R1). According to these comments and suggestions, we have made careful modifications accordingly. In the revised version, changes to our manuscript were all marked with a tracked pattern. Point-by-point responses to the nice associate editor and nice reviewers are listed below this letter.

Yours sincerely,

Bao-Peng Liu, PhD

E-mail: baopeng.liu@sdu.edu.cn

Department of Epidemiology, School of Public Health, Cheeloo College of Medicine, Shandong University, Jinan, China.

Cun-Xian Jia, PhD

E-mail: jiacunxian@sdu.edu.cn

Department of Epidemiology, School of Public Health, Cheeloo College of Medicine, Shandong University, Jinan, China.

**Comments from the academic editor and the editorial team:**

**Please acknowledge the lack of validation in an independent dataset as a limitation of the study.**

**Response:** We thank the editor and the editorial team for the valuable suggestion. We acknowledge that the lack of validation in another independent dataset is a limitation of the study. While the UK Biobank provides a large and well-characterized cohort, the findings of this study should be interpreted with caution until they can be replicated in other independent cohorts. We have included this limitation in the revised manuscript under the Discussion section of the revised manuscript.

**Discussion section (Page 25, Lines 522-524):** “*Additionally, the lack of validation in another independent dataset is a limitation. While the UK Biobank cohort is large and well-characterized, these findings should be replicated in other independent cohorts.*”

**Comments from the reviewers:**

**Reviewer #1: Review of the article PMEDICINE-D-25-03230R1 "Physical frailty, genetic risk, mediating biomarkers, and risk of suicide attempt: a prospective cohort study"**

**This article is a prospective analysis of the association of frailty and suicidal attempt in the UK Biobank cohort study. The authors also analysed the modifying and mediating roles of genetic risk and biomarkers. They found that pre-frail and frail status was associated with increased risk of suicidal attempt.**

**The article is well-written, the methodology is robust, the research question is relevant and quite novel, and the results are derived from the data.**

**Some comments:**

**1. There is a missing reference:**

**Draper B, Wand APF. Suicide in later life: the role of frailty and depression. Curr Opin Psychiatry. 2025;38(5):383-388. doi:10.1097/YCO.0000000000001009**

**Response:** We thank you for this suggestion. We have now cited the review by Draper and Wand (2025) and incorporated it into both the Introduction and the Discussion sections to better contextualize the existing evidence on frailty and suicide-related outcomes in later life.

**Introduction section (Page 5, Lines 75-77):** “*In line with this perspective, a recent narrative review has highlighted frailty as a potentially important factor in late-life suicidal ideation (SI) and SA, although the existing evidence is largely qualitative in nature [16].*”

**Discussion section (Page 20, Lines 405-407):** “*Although a recent review suggested that frailty may be an important underlying factor in SA during later life, the evidence is primarily based on qualitative studies [16], with only one quantitative study available [15].*”

**2. In the sensitivity analyses, the authors used multiple imputation to deal with missing covariates. Can they give more details about the imputation procedure? Did they apply Rubin's rules to pool estimates and calculate confidence intervals? What was the percentage of missing values for each covariate?**

**Response:** We thank you for this comment. We have now provided additional details on the multiple imputation procedure in the Methods section. Specifically, we report that missing data were minimal overall, with a summary of missing values for each covariate presented in Supplementary Table S4; the highest proportion of missingness was observed for educational level (0.87%). For the sensitivity analyses, missing covariates were handled using multiple imputation. We generated five imputed datasets with 20 iterations to ensure convergence and stability, conducted Cox proportional hazards regression analyses within each imputed dataset, and pooled the resulting estimates and confidence intervals using Rubin’s rules. These clarifications have been added to the Methods section of the revised manuscript.

**Methods section (Page 11, Lines 214 and 215):** “*A summary of missing data is provided in Table S4, with the highest proportion of missing values recorded for educational level (0.87%).*”

**Methods section (Pages 14 and 15, Lines 284-287):** “*Specifically, we generated five imputed datasets with 20 iterations to ensure convergence and stability of the imputation process and pooled the estimates from Cox proportional hazards regression models across imputed datasets using Rubin’s rules [43].*”

**3. How were the variables for adjustment chosen? Was a directed acyclic graph (DAG) used for the identification of confounders, mediators and colliders?**

**Response:** We thank you for this important suggestion. In the original analysis, covariates were selected a *priori* based on existing literature demonstrating associations of sociodemographic factors, lifestyle behaviors, and medical histories with both physical frailty and suicide-related outcomes. Following your recommendation, we have now additionally developed and incorporated a directed acyclic graph (DAG) to more explicitly clarify the assumed causal relationships among exposure, outcome, and covariates. The DAG supports the covariate selection used in the primary models and provides a transparent conceptual framework for the analytic strategy. We have provided the corresponding clarifications in the Methods section of the revised manuscript.

**Methods section (Page 11, Lines 211-213):** “*These covariates were selected a priori based on previous studies demonstrating their associations with both physical frailty and SA [3, 6, 13], with a directed acyclic graph (DAG) additionally used to support the identification of confounders (S2 Fig).*”

**4. How were the selected biomarkers selected?**

**Response:** We thank you for this question. The biomarkers were selected a priori based on existing epidemiological literature. Specifically, we focused on blood biomarkers that reflect key biological pathways relevant to frailty, psychiatric disorders, or suicide-related outcomes and that have been commonly examined in prior UK Biobank-based studies. For example, Gao et al. used UK Biobank data to investigate the potential mediating roles of a broad panel of blood biomarkers in the association between physical frailty and neuropsychiatric disorders [1], while Zhang et al. examined behaviour-related and physiological risk factors for suicide attempt in the UK Biobank, similarly incorporating these biomarkers [2]. Guided by these prior studies, we selected 61 externally validated blood biomarkers representing immunometabolic, endocrine, hepatic, renal, hematological, bone, and platelet-related pathways as potential mediating biomarkers. We have made the corresponding clarifications in the Methods section of the revised manuscript.

**Methods section (Pages 10 and 11, Lines 198-202):** “*Guided by prior knowledge and existing literature [5, 6, 8, 10-15], 61 blood biomarkers reflecting diverse biological pathways, such as liver and kidney function, immunometabolism, endocrine function, red and white blood cells, bone health, and platelets, were selected as potential mediators linking physical frailty and SA. Further details on the selected biomarkers are provided in the S1 Methods and S2 Table.*”

**Reviewer #2: This is an interesting study on the association among physical frailty, genetic risk, mediating biomarkers, and risk of suicide attempt. However, there are a few major issues needing attention.**

**1. Not sure or convinced by the premise of the study that physical frailty is the main driver of suicide attempt with all the other factors as either covariates or mediation factors. It could be other way around that people with severe long-term conditions both physically and/or mentally plus siocioeconomic disadvantages are more likely to develop frailty as both a condition and a set of symptoms in the course leading to SA, therefore frailty could be a mediator itself for assessing the associations of the above mentioned diseases.**

**Response:** We thank you for this thoughtful and important comment. We agree that the temporal and causal relationships among chronic physical and mental conditions, socioeconomic disadvantage, frailty, and suicide attempt (SA) are complex, and that frailty could plausibly act as either an exposure or a mediator in different conceptual frameworks.

**First**, our primary aim was to prospectively examine whether baseline physical frailty is associated with an increased risk of incident SA. To reduce reverse causation, we excluded participants with SA at baseline, and outcomes were ascertained during follow-up. Also, in the prior sensitivity analysis, we excluded participants who experienced SA within the first two years of follow-up, thereby further reducing the potential for reverse causality bias. **Second**, prior studies have shown that physical frailty is a strong risk factor for severe physical conditions (e.g., cardiovascular disease and cancer) as well as psychiatric disorders [1, 3, 4], and these conditions are, in turn, well-established risk factors for SA. Therefore, severe physical and psychiatric disorders could plausibly lie on the pathway linking frailty to SA and may act as potential mediators of the frailty-SA association. However, in our main association analyses we adjusted for these factors that could contribute to both frailty and SA, including severe physical conditions (CVD and cancer), psychiatric disorders, and Townsend Deprivation Index (TDI, with higher values indicating greater socioeconomic deprivation). The frailty-SA association remained statistically significant after these adjustments, indicating that frailty is associated with SA risk, independent of these risk factors. **Further**, we conducted a series of additional sensitivity analyses aimed at minimizing the possibility that the observed association is primarily driven by pre-existing severe physical illness, psychiatric disorders, or socioeconomic disadvantage. We firstly excluded participants with baseline cardiovascular disease, cancer, or psychiatric disorders. As shown in the **Response Table 1**, the association between physical frailty and SA remained materially unchanged. To further account for socioeconomic disadvantage, we restricted the analysis to participants with TDI values at or below the median, thereby excluding individuals with higher levels of deprivation. The association between physical frailty and SA persisted in this restricted sample (**Response Table 1**). Hence, although causality cannot be inferred from observational studies, these findings indicate that the association between frailty and SA is not driven solely by any one category of severe physical illness, psychiatric morbidity, or socioeconomic disadvantage, lending support to frailty as a potential independent contributor to SA risk. We have made corresponding revisions in the Methods and Results sections of the revised manuscript. The results are presented in the Supplementary Materials.

**Methods section (Page 15, Lines 287-290):** “*Third, to further minimize potential confounding from severe pre-existing physical and mental conditions and socioeconomic disadvantage, additional sensitivity analyses were conducted excluding participants with baseline CVD, cancer, or psychiatric disorders, as well as those with greater socioeconomic deprivation.*”

**Results section (Page 18, Lines 370-372):** “*When participants with baseline CVD, cancer, or psychiatric disorders and those with higher socioeconomic deprivation were excluded, the associations between frailty status and SA risk remained robust (S15 Table).*”

# Response Table 1. Associations of frailty status with the risk of suicide attempt after excluding participants with baseline CVD, cancer, psychiatric disorders, or higher deprivation.

| **Frailty status** | **Excluding participants with baseline CVD or cancer or** **psychiatric disorders (*N* = 316,825)** | | **Excluding participants with higher levels of deprivation (TDI > median) (*N* = 221,998)** | |
| --- | --- | --- | --- | --- |
|  | **HR^a^ (95% CI)** | ***P* value** | **HR^b^ (95% CI)** | ***P* value** |
| Non-frail (0) | 1(Reference) |  | 1(Reference) |  |
| Pre-frail (1-2) | 1.71 (1.45-2.03) | <0.001 | 1.88 (1.58-2.23) | <0.001 |
| Frail (3-5) | 2.61 (1.80-3.78) | <0.001 | 2.02 (1.35-3.04) | <0.001 |
| *P* for trend | <0.001 | | <0.001 | |
| Per 1-point increase | 1.43 (1.31-1.56) | <0.001 | 1.40 (1.28-1.53) | <0.001 |

Note: Abbreviations: HR, hazards ratio; CI, confidence interval; TDI, Townsend Deprivation Index.

^a^Models were adjusted for age, sex, education, employment, TDI, genetic risk, smoking status, drinking frequency, and body mass index.

^b^Models were adjusted for age, sex, education, employment, genetic risk, smoking status, drinking frequency, body mass index, pre-existing psychiatric disorders, cardiovascular diseases, and cancer.

**2. Table 2 - Associations of frailty status with the risk of suicide attempt, is the main results table of the study. However, only Model 3 is needed and rest models are redundant. Two issues, 1) Without fully adjusting for all the co-morbidities with detailed subtype, serverity and stages, the reliability and robustness of results of Model 3 are subject to scrutiny. So far only pre-existing psychiatric disorders, cardiovascular diseases, and cancer were included in the model, and it is far from comprehensive and complete.**

**Response:** We thank you for this insightful comment. We agree that Model 3 represents the fully adjusted primary model and is the most relevant for inference. Models 1 and 2 were retained to demonstrate the stepwise impact of covariate adjustment, which is common practice in epidemiological studies [5, 6]. Moreover, we have clarified in the revised manuscript that Model 3 represents the primary inference. Regarding residual confounding by comorbidities, we acknowledge that it is not feasible to comprehensively adjust for all diseases with detailed subtypes, severity, and stages in a single model. To address this concern and to strengthen the robustness of our findings, we conducted two additional sensitivity analyses. First, we further adjusted the fully adjusted model by incorporating the Charlson Comorbidity Index (CCI), which captures overall comorbidity burden in a standardized manner [7, 8]. As shown in the **Response Table 2**, the findings of the sensitivity analysis were consistent with the primary findings, supporting the robustness of the observed association. Second, we calculated E-values to quantify the minimum strength of association that an unmeasured confounder would need to have with both frailty and SA to fully explain away the observed associations [9]. The E-values indicated that the frailty-SA association showed strong robustness, as an unmeasured confounder would need to be associated with both exposure and outcome by a risk ratio of nearly 3.0 to fully explain the observed association (**Response Table 3**). Relevant descriptions have been added to the Methods and Results sections of the revised manuscript, and the results are presented in the Supplementary Materials.

**Methods section (Page 12, Line 233):** “*…and Model 3 (the primary model) further incorporated medical histories.*”

**Methods section (Page 15, Lines 290-293):** “*Fourth, to further address potential residual confounding by comorbidities, we additionally adjusted for the Charlson Comorbidity Index (CCI) (S6 Table) [44]. We also calculated E-values to quantify the minimum strength of association that an unmeasured confounder would need to have with both frailty status and SA to fully explain the observed associations [45].*”

**Results section (Pages 18 and 19, Lines 372-377):** “*Additional adjustment for the CCI also did not materially alter the results (S16 Table), suggesting that residual confounding by comorbidity burden was unlikely to fully explain the observed associations. E-value analyses indicated substantial robustness to unmeasured confounding, as an unmeasured confounder would need to be associated with both frailty status and SA by a risk ratio of approximately 3.0 to fully account for the observed associations (S17 Table).*”

# Response Table 2. Associations of frailty status with the risk of suicide attempt after further adjustment for the Charlson Comorbidity Index.

| **Frailty status** | **Model 3 + the Charlson Comorbidity Index (*N* = 442,920)** | |
| --- | --- | --- |
|  | **HR (95% CI)** | ***P* value** |
| Non-frail (0) | 1(Reference) |  |
| Pre-frail (1-2) | 1.60 (1.43-1.79) | <0.001 |
| Frail (3-5) | 2.11 (1.74-2.57) | <0.001 |
| *P* for trend | <0.001 | |
| Per 1-point increase | 1.29 (1.22-1.35) | <0.001 |

Abbreviations: HR, hazards ratio; CI, confidence interval.

Note: Model 3 was adjusted for age, sex, education, employment, Townsend Deprivation Index, genetic risk, smoking status, drinking frequency, body mass index, pre-existing psychiatric disorders, cardiovascular diseases, and cancer.

# Response Table 3. E-values for the associations between frailty status and suicide attempt.

| **Exposure comparison** | **HR (95% CI) in Model 3** | **E-value**  **(point estimate)** | **E-value**  **(lower 95% CI)** |
| --- | --- | --- | --- |
| Pre-frailty vs non-frailty | 1.61 (1.44-1.80) | 2.60 | 2.24 |
| Frailty vs non-frailty | 2.16 (1.78-2.61) | 3.74 | 2.96 |

Abbreviations: HR, hazards ratio; CI, confidence interval.

Note: Model 3 was adjusted for age, sex, education, employment, Townsend Deprivation Index, genetic risk, smoking status, drinking frequency, body mass index, pre-existing psychiatric disorders, cardiovascular diseases, and cancer. E-values quantify the minimum strength of association that an unmeasured confounder would need to have with both the exposure (frailty status) and the outcome (suicide attempt), beyond the measured covariates, to fully explain away the observed associations.

**3. A complete results table for Table 2 is needed so that we can see the contribution from all the factors, especially the PRS contribution is missing from the table.**

**Response:** We thank you for this constructive suggestion. Following your recommendation, we have revised Table 2 to include the association between polygenic risk score (PRS) and the risk of suicide attempt. By incorporating PRS into the main results table, Table 2 now provides a more complete presentation of the contributions from both frailty status and genetic risk within the same analytical framework. The manuscript has been updated accordingly.

# Table 2. Associations of frailty status and genetic risk with the risk of suicide attempt.

|  | **Cases/**  **person-years** | **Model 1** | | **Model 2** | | **Model 3** | |
| --- | --- | --- | --- | --- | --- | --- | --- |
|  |  | **HR (95% CI)** | ***P* value** | **HR (95% CI)** | ***P* value** | **HR (95% CI)** | ***P* value** |
| **Frailty status**^a^ |  |  |  |  |  |  |  |
| Non-frail (0) | 570/3,454,579 | 1(Reference) |  | 1(Reference) |  | 1(Reference) |  |
| Pre-frail (1-2) | 782/2,218,892 | 1.86 (1.67-2.08) | <0.001 | 1.80 (1.61-2.02) | <0.001 | 1.61 (1.44-1.80) | <0.001 |
| Frail (3-5) | 166/194,313 | 3.06 (2.54-3.69) | <0.001 | 2.79 (2.30-3.38) | <0.001 | 2.16 (1.78-2.61) | <0.001 |
| *P* for trend |  | <0.001 | | <0.001 | | <0.001 | |
| Per 1-point increase | 1,518/5,867,785 | 1.44 (1.37-1.51) | <0.001 | 1.40 (1.33-1.47) | <0.001 | 1.29 (1.23-1.36) | <0.001 |
| PARP^b^ |  | 28.5% (23.5%-33.4%) | | 27.4% (22.3%-32.6%) | | 21.3% (15.5%-27.1%) | |
| **Genetic risk**^c^ |  |  |  |  |  |  |  |
| Low | 337/1,958,029 | 1(Reference) |  | 1(Reference) |  | 1(Reference) |  |
| Intermediate | 506/1,956,827 | 1.42 (1.23-1.63) | <0.001 | 1.40 (1.21-1.60) | <0.001 | 1.36 (1.18-1.56) | <0.001 |
| High | 675/1,952,929 | 1.77 (1.55-2.02) | <0.001 | 1.72 (1.50-1.96) | <0.001 | 1.62 (1.42-1.85) | <0.001 |
| Per SD increment | 1,518/5,867,785 | 1.30 (1.24-1.37) | <0.001 | 1.29 (1.22-1.36) | <0.001 | 1.25 (1.19-1.32) | <0.001 |

*Note*: ^a^Frailty status was assessed using a frailty phenotype score ranging from 0 to 5 and categorized as non-frail (0), pre-frail (1-2), and frail (3-5). ^b^PARP indicated the proportion of suicide attempt that could theoretically be prevented in this study population if the entire population could have been maintained non-frail. Population attributable risk at the median follow-up time of the study population was reported. ^c^Genetic risk was evaluated using a polygenic risk score and categorized into low, intermediate, and high genetic risk based on tertiles of the score distribution. For the genetic risk model, Model 1 was additionally adjusted for the first 10 principal components of ancestry.

Model 1 adjusted for age, sex, education, employment, Townsend Deprivation Index, and genetic risk (for the frailty model) or frailty status (for the genetic risk model).

Model 2 adjusted for Model 1 plus smoking status, drinking frequency, and body mass index.

Model 3 adjusted for Model 2 plus pre-existing psychiatric disorders, cardiovascular diseases, and cancer.

Abbreviations: HR, hazards ratio; CI, confidence interval; SD, standard deviation; PARP, population attributable risk percent.

**4. Competing risk. How many people died during the 13-year follow up? For survival analysis with an end point other that all cause mortality, a competing risk analysis is needed (competing risk due to death).**

**Response:** We thank you for raising this important methodological point. During the 13-year follow-up period, a total of 37,315 participants died. To account for death as a competing event in the analysis of suicide attempt, we additionally performed a competing risk analysis using the Fine-Gray subdistribution hazards model, treating death as the competing risk. The results from the competing risk model were consistent with those of the primary survival analysis (**Response Table 4**). These findings suggest that the observed associations are robust and not driven by competing risk due to death. Relevant descriptions have been added to the Methods and Results sections of the revised manuscript, and the results are presented in the Supplementary Materials.

**Methods section (Page 15, Lines 294-296):** “*Fifth, given that death could preclude the occurrence of SA during follow-up, we conducted a competing risk analysis using the Fine-Gray subdistribution hazards model, treating death as a competing event.*”

**Methods section (Page 19, Lines 377-378):** “*Furthermore, results from the competing risk analysis accounting for death as a competing event were consistent with those from the primary Cox proportional hazards models (S18 Table).*”

# Response Table 4. Associations of frailty status with the risk of suicide attempt accounting for death as a competing risk.

| **Frailty status** | **Accounting for death as a competing risk (*N* = 442,920)** | |
| --- | --- | --- |
|  | **sHR (95% CI)** | ***P* value** |
| Non-frail (0) | 1(Reference) |  |
| Pre-frail (1-2) | 1.56 (1.37-1.77) | <0.001 |
| Frail (3-5) | 2.01 (1.60-2.52) | <0.001 |
| *P* for trend | <0.001 | |
| Per 1-point increase | 1.25 (1.18-1.32) | <0.001 |

Abbreviations: sHR, subdistribution hazards ratio; CI, confidence interval.

Note: Models were adjusted for age, sex, education, employment, Townsend Deprivation Index, genetic risk, smoking status, drinking frequency, body mass index, pre-existing psychiatric disorders, cardiovascular diseases, and cancer.

**5. Page 5, it says "participants with incomplete information on frailty components (n = 14,824) were excluded". This could bias the results. A comparison table is needed to compare those included with those excluded.**

**Response:** We thank you for this important comment and apologize for the confusion caused by an error in the originally reported number. Due to a typographical mistake, the number of participants excluded because of incomplete information on frailty components was incorrectly stated. In fact, 37,211 participants were excluded for missing frailty component data, as correctly shown in the study flowchart provided in the Supplementary Material (**Figure S1**). We have now corrected this number consistently throughout the manuscript. In addition, following your suggestion, we compared the baseline characteristics between participants included in the final analysis (*N* = 442,920) and those excluded from the study population (*N* = 59,381). As shown in **Response Table 5**, excluded participants were slightly older, more likely to be female, had lower educational attainment and employment rates, higher socioeconomic deprivation, and more likely to be current smokers, as well as a higher prevalence of psychiatric disorders, cardiovascular diseases, and cancer. We have now explicitly addressed the potential selection bias arising from participant exclusion in the **Limitations** section of the revised manuscript. Specifically, we discuss the differences in baseline characteristics between included and excluded participants and note that, while *P* values were statistically significant for most comparisons due to the large sample size, the standardized mean differences were generally small, suggesting that the risk of substantial selection bias is likely limited. The following revisions have been added to the Methods and Discussion sections of the revised manuscript, and the corresponding results are presented in the Supplementary Materials.

**Methods section (Page 7, Line 130):** “*Next, participants with incomplete information on frailty components (n = 37,211) were excluded.*”

**Discussion section (Page 25, Lines 512-515):** “*Also, potential selection bias due to participant exclusion cannot be entirely ruled out. While P values were statistically significant for most comparisons due to the large sample size, the standardized mean differences were generally small, suggesting that the risk of substantial selection bias is likely limited (S23 Table).*”

**Figure S1. Flowchart of the selection of the study population**

**Response Table 5. Comparison of baseline characteristics between included and excluded populations**

| **Characteristics** | **Included** | **Excluded** | ***P* value** | **SMD** **absolute value** |
| --- | --- | --- | --- | --- |
|  | **(*N* = 442,920)** | **(*N* = 59,381)** |  |  |
| Age, mean (SD) | 56.5 (8.1) | 56.7 (8.3) | <0.001 | 0.024 |
| Female, n (%) | 239,686 (54.1) | 33,569 (56.5) | <0.001 | 0.048 |
| College or university degree, n (%) | 147,969 (33.4) | 13,109 (26.6) | <0.001 | 0.148 |
| Employed, n (%) | 409,567 (92.5) | 48,053 (85.2) | <0.001 | 0.232 |
| Townsend deprivation index, mean (SD) | -1.4 (3.0) | -0.5 (3.4) | <0.001 | 0.281 |
| Drinking alcohol ≥ 3 times/week, n (%) | 197,052 (44.5) | 20,079 (34.7) | <0.001 | 0.200 |
| Current smokers, n (%) | 44,973 (10.2) | 7978 (14.1) | <0.001 | 0.119 |
| Overweight or obesity, n (%) | 295,013 (66.6) | 39,231 (66.1) | 0.009 | 0.011 |
| Medical histories, n (%) |  |  |  |  |
| Psychiatric disorders | 73,022 (16.5) | 12,343 (20.8) | <0.001 | 0.110 |
| CVD | 28,942 (6.5) | 5,477 (9.2) | <0.001 | 0.100 |
| Cancer | 40,078 (9.0) | 5,695 (9.6) | <0.001 | 0.021 |

Abbreviations: CVD, cardiovascular diseases; SD, standard deviation; SMD, standardized mean difference.

**6. MR analysis is difficult to follow, and a bit redundant as it seems to force the causality issue between fraity and SA, which doesn't really make sense.**

**Response:** We thank you for this insightful comment and appreciate the opportunity to clarify the role of the Mendelian randomization (MR) analysis in our study. We understand the concern regarding the potential overemphasis on causality in our original manuscript. The primary focus of our study is the prospective association between physical frailty and suicide attempt (SA), and the MR analysis, as a complementary approach, was intended to provide genetic evidence to explore the association rather than to establish causality. The inclusion of MR analyses follows a common practice in prior UK Biobank-based cohort studies [10-12], where genetic instruments are used to strengthen causal inference and to address residual confounding and reverse causation that cannot be fully eliminated in observational analyses. In the revised manuscript, we have clarified the supportive role of MR analyses. Specifically, we emphasize that MR provided additional genetic evidence that is consistent with the observed association between frailty and SA, without over-interpreting this as definitive proof of causality. We have also modified the relevant sections to avoid overstating causal claims and to ensure that the primary findings from the cohort analysis remain the central focus of the paper. The following revisions can be found in the revised manuscript.

**Abstract (Methods and findings):** “*Mendelian randomization (MR) analyses were utilized to examine the association between genetically determined physical frailty and SA.*”

**Abstract (Methods and findings):** “*Genetically predicted frailty was also positively associated with SA (odds ratio = 2.06, 95% CI [1.21-3.52]; P = 0.008).*”

**Introduction section (Page 7, Lines 117 and 118):** “*Further, we employed two-sample MR analyses as a complementary approach to explore whether the observed association shows genetic support consistent with the cohort findings.*”

**Methods section (Page 12, Lines 237-239):** “*Two-sample MR analyses were conducted as a secondary and supportive analysis to provide genetic evidence. Compared with conventional observational analyses, this approach is less susceptible to confounding and reverse causation [37].*”

**Results section (Page 16, Lines 321-323):** “*In secondary analyses, MR results were directionally consistent with the findings from the cohort analysis, showing that genetically determined frailty was associated with an increased risk of SA.*”

**Discussion section (Page 19, Lines 387 and 388):** “*Two-sample MR analyses provided complementary genetic evidence supporting the observed association.*”

**Discussion section (Page 20, Lines 416-418):** “*Two-sample MR analyses provided additional support for the genetic association between FP-measured physical frailty and SA, suggesting that the observed association may be consistent with a potential causal relationship.*”

**Conclusions section (Page 26, Lines 533-535):** “*The cohort study demonstrated that physical frailty was associated with an increased risk of SA, with MR analyses providing complementary genetic evidence consistent with this association.*”

**Reviewer #3: Hu and colleagues investigate the role of frailty in the risk of suicide attempt. This is an interesting goal, and the approach used is justified. However, there are several issues that the authors should consider to increase the impact of their investigation.**

**1. In addition to suicide attempt, it could be interesting to assess whether frailty has a similar impact on suicidal ideation and whether the effect on suicidal attempt is related at least partially to its relationship with suicidal ideation. This may have important public health implications.**

**Response:** We appreciate your insightful suggestion to investigate the impact of frailty on suicidal ideation (SI) alongside suicide attempt (SA). While our initial focus was primarily on the association between frailty and SA, we agree that exploring the relationship between frailty, SI, and SA could have important public health implications. We have since incorporated an analysis of the association between frailty and SI in our study. Our findings indicate that frailty does indeed have a similar impact on SI, and that SI partially mediates the relationship between frailty and SA, accounting for approximately one-third of the frailty-SA association (**Response Table 6**). We have updated the discussion section of our manuscript to highlight these new findings and emphasize the role of SI as a crucial factor in the frailty-SA relationship.

**Discussion section (Page 22, Lines 454-460):** “*These psychosocial sequelae may accumulate as frailty progresses, intensifying emotional distress and diminishing perceived social connectedness and coping resources [50]. In line with this, we observed that frailty also significantly impacts SI, and that SI plays a key role in the frailty-SA relationship. Approximately one-third of the frailty-SA association could be explained by SI (S22 Table). Thus, in addition to physical vulnerability, frailty may contribute to SA through both psychosocial adversity and emotional distress that facilitate the transition from SI to SA.*”

# Response Table 6. Associations between physical frailty and suicidal ideation, and mediating role of suicidal ideation in the frailty-SA association

| **Frailty status** | **Association between frailty and suicidal ideation**^a^ **(*N* = 143,773)** | | **Mediating role of suicidal ideation in the frailty-SA association (*N* = 143,773)** | |
| --- | --- | --- | --- | --- |
|  | **HR (95% CI)** | ***P* value** | **PM (95% CI)** | ***P* value** |
| Non-frail (0) | 1(Reference) |  | 32.8%  (31.3%-34.1%) | < 0.001 |
| Pre-frail (1-2) | 1.75 (1.65-1.84) | <0.001 |  |  |
| Frail (3-5) | 3.55 (3.12-4.03) | <0.001 |  |  |
| *P* for trend | <0.001 | |  |  |
| Per 1-point increase | 1.53 (1.48-1.58) | <0.001 |  |  |

Abbreviations: HR, hazard ratio; CI, confidence interval; SA, suicide attempt; PM, Proportion mediated.

Note: ^a^Suicidal ideation was measured based on self-reported data from participants in the online follow-up, with approximately one-third of baseline participants providing relevant information. Models were adjusted for age, sex, education, employment, Townsend Deprivation Index, genetic risk, smoking status, drinking frequency, body mass index, pre-existing psychiatric disorders, cardiovascular diseases, and cancer.

**2. The biomarker analysis is interesting. However, the authors should consider expanding it further using a one-sample MR design to assess whether the associations observed are due to cause-and-effect relationships.**

**Response:** We thank you for this insightful and constructive comment. We agree that the MR analysis can provide an important complementary approach to strengthen causal inference beyond observational mediation analyses. However, in the present study, a one-sample MR design was not feasible, as the genetic associations for suicide attempt (SA) were derived from large external genome-wide association studies, whereas individual-level data on SA within the UK Biobank are limited due to the rarity of the outcome and concerns regarding statistical power. Accordingly, our primary analyses assessing the genetic association between frailty and SA were conducted using a two-sample MR framework, which is widely adopted in psychiatric genetics and suicide research.

To address your concern regarding causality in the biomarker mediation analysis, we therefore implemented a two-step MR approach within a two-sample MR framework, which has been recommended and widely applied to evaluate putative mediators in exposure-outcome relationships [13, 14]. Specifically, in the first step, we assessed the causal effect of genetically predicted frailty on the biomarker, and in the second step, we examined the causal effect of the biomarker on suicide risk. Evidence for a causal mediating role was considered present only if both steps demonstrated statistically significant associations in the expected direction. Notably, although the observational mediation analyses suggested partial mediation by biomarkers related to liver function, metabolism, immunity, and inflammation, the two-step MR analyses did not provide statistically significant evidence supporting a causal mediating role for these biomarkers. The discrepancy between the observational mediation analysis and the MR findings may be attributable to several factors. First, genetic instruments for circulating biomarkers typically explain only a small proportion of phenotypic variance, which substantially limits statistical power in MR analyses, particularly when the outcome of interest, SA, is relatively rare and etiologically heterogeneous. Second, the biomarkers identified in the observational analyses may predominantly reflect downstream consequences of frailty or correlated biological processes, rather than functioning as direct causal mediators in the pathway linking frailty to suicide risk. The following revisions can be found in the revised manuscript.

**Abstract section (Page 3, Lines 24-26):** “*Biomarkers related to liver function, metabolism, immunity, and inflammation may have partially explained this association, accounting for a collective 14.15% (95% CI: 8.94%-19.74%) of the total effect, although MR analyses did not support causal mediating effects.*”

**Methods section (Page 14, Lines 271-278):** “*To further investigate whether the biomarkers identified in the observational mediation analysis lie on the causal pathway linking physical frailty to SA, we conducted two-step MR analyses [42]. In the first step, genetic instruments for physical frailty were used to estimate the causal effect of frailty on each candidate biomarker. In the second step, genetic instruments for the corresponding biomarker were applied to assess its causal effect on SA. Details regarding the selection of genetic instruments, quality control procedures, and MR estimation methods are provided in the S1 Methods. Evidence for causal mediating roles was considered present only if both steps demonstrated statistically significant associations in the expected direction.”*

**Results section (Page 18, Lines 356-360):** “*Each of these biomarkers demonstrated a statistically significant indirect effect in the observational mediation analyses (all P_FDR_ < 0.05), with the PM ranging from 0.23% (95% CI: 0.06%-0.41%) to 3.53% (95% CI: 1.72%-5.23%) (Figure 3).*”

**Results section (Page 18, Lines 361 and 362):** “*However, subsequent two-step MR analyses did not provide statistically significant evidence supporting a causal mediating role for these biomarkers (S12 Table).*”

**Discussion section (Page 19, Lines 390-392):** “*Furthermore, we found that approximately 14.2% of the frailty-SA association could be statistically explained by biomarkers related to liver function, metabolism, immune function, and inflammation.*”

**Discussion section (Page 24, Lines 486-494):** “*Notably, subsequent two-step MR analyses did not provide statistically significant evidence supporting a causal mediating role for these biomarkers. The discrepancy between the observational mediation and MR findings may reflect several factors. First, genetic instruments for circulating biomarkers typically explain limited phenotypic variance, substantially reducing statistical power in MR analyses, particularly for rare and etiologically heterogeneous outcomes such as SA. Second, the biomarkers identified in observational analyses may primarily represent downstream correlates of frailty or shared biological processes, rather than direct causal mediators in the frailty-SA pathway. Therefore, these findings should be interpreted as indicating potential biological pathways rather than confirmed causal intermediates.*”

**Conclusion section (Page 26, Lines 537-540):** “*Furthermore, a range of biomarkers related to liver function, metabolism, immune function, and inflammation statistically explained a modest proportion of this association, highlighting potential biological pathways accompanying frailty. However, these biomarkers should be interpreted as potential contributors rather than confirmed causal mediators.*”

**3. In addition to the STROBE checklist for genetic studies, the authors should report whether their MR analyses are in line with the STROBE-MR checklist (PMID: 34698778).**

**Response:** We thank you for this helpful suggestion. In addition to reporting the cohort study according to the STROBE guideline, we have now clarified that our Mendelian randomization analyses were conducted and reported in line with the STROBE-MR checklist [15]. The relevant statement has been added to the Methods section of the revised manuscript.

**Methods section (Page 13, Lines 252 and 253):** “*The MR study followed the STROBE-MR guidelines (S2 Checklist) [39].*”

**4. The authors provide limited information regarding the genetically informed ancestry assignment to define their cohort. Additionally, they use incorrect terminology to describe population groups (e.g., "... constructed for White populations"). They should follow the recommendations of the National Academies regarding population descriptors for genetic studies (PMID: 36989389). Also, I recommend them the genetically informed ancestry assignment of the Pan-UKBB Initiative (PMID: 40968291).**

**Response:** Thank you for this important comment. We agree that our previous description of the cohort definition provided insufficient detail on genetically informed ancestry assignment and used imprecise population terminology (e.g., “White populations”). Following the National Academies’ recommendations on population descriptors in genetics and genomics research [16], we revised the manuscript to (i) clearly distinguish genetically inferred ancestry from self-identified race/ethnicity, (ii) avoid typological racial terms such as “White/non-White,” and (iii) transparently report how ancestry was assigned and how exclusions were performed. Specifically, we updated our methods to use the Pan-UK Biobank (Pan-UKBB) genetically inferred ancestry assignments [17], which compare each UKB participant’s genotype data to global reference panels (HGDP + 1000 Genomes) and assign individuals to the ancestry group they are most genetically similar to, while excluding individuals without a confident assignment. This approach does not rely on self-reported ethnicity. Because the polygenic risk score (PRS) used in our study was derived primarily in individuals of European genetic ancestry [18], we excluded participants with missing genetic data or assigned to non-European genetically inferred ancestry groups. The relevant statement has been added to the Methods section of the revised manuscript.

**Methods section (Pages 7 and 8, Lines 130-139):** “*To define genetically informed ancestry for analyses involving polygenic risk scores, we used genetically inferred ancestry assignments following the Pan-UK Biobank (Pan-UKBB) framework [27], which compares each participant’s genome-wide genotype data to global reference panels (HGDP and the 1000 Genomes Project) and assigns individuals to the ancestry group they are most genetically similar to, while excluding individuals without a confident assignment. This assignment is based on genetic similarity and does not rely on self-reported ethnicity [28]. Because the genetic tool used to compute the SA-PRS was derived and validated primarily in individuals of European genetic ancestry [25], we excluded participants with missing genetic data or assigned to non-European genetically inferred ancestry groups (n = 11,253).*”

**5. Since the suicide-attempt GWAS of the Psychiatric Genomics Consortium includes the UK Biobank, the authors should clearly state that they obtained a version of the GWAS data that exclude UK Biobank, and this is different from the one described in the reference cited.**

**Response:** Thank you for the important comment. We agree that this issue requires clearer clarification to avoid any potential misunderstanding. In the originally published genome-wide association study (GWAS) meta-analysis of suicide attempt, a total of 15 cohorts of European ancestry were included, one of which was the UK Biobank. Detailed descriptions of the contributing cohorts have been provided in the original publication [18]. As UK Biobank data were also used in other analyses of the present study, we did not use the publicly released GWAS summary statistics described in that publication in order to avoid potential sample overlap. Instead, we used a UK Biobank-excluded SA GWAS meta-analysis that was officially generated and provided by the Psychiatric Genomics Consortium (PGC). This UK Biobank-excluded GWAS was based on the remaining 14 independent cohorts and included a total sample size of 4,779,791 participants (**Response Table 7**). Notably, this version of the SA GWAS has been used in previous UK Biobank studies for the construction of SA polygenic risk scores (SA-PRS) [2]. Importantly, this UK Biobank-excluded GWAS summary dataset was produced by the PGC using the same analytical pipeline and methodology as the originally published PGC suicide attempt GWAS, differing only in the exclusion of the UK Biobank cohort from the sample composition. In the original submission, information regarding the source of the GWAS summary statistics and the handling of potential sample overlap was provided in the Supplementary Methods. To improve clarity, transparency, and accessibility for readers, we have now explicitly incorporated these details into the main Methods section of the revised manuscript. We clearly state that the GWAS summary statistics used in the present study differ from the originally published suicide attempt GWAS and correspond to the UK Biobank–excluded version obtained directly from the PGC through the Suicide Attempt Data Access Portal (https://pgc.unc.edu/for-researchers/data-access-committee/data-access-portal/).

The following revisions have been added to the Methods sections of the revised manuscript.

**Methods section (Pages 9 and 10, Lines 169-194):** “*This study utilized genetic data from the UK Biobank, restricting analyses to participants of European genetic ancestry, selected following established procedures [31]. Detailed descriptions of the genotyping and quality control procedures implemented by the UK Biobank have been reported previously [31]. Variants were filtered using standard quality control criteria, including minor allele frequency ≥ 0.01, imputation INFO score ≥ 0.3, Hardy-Weinberg equilibrium P-value ≥ 1×10^-6^, and a genotyping rate of ≥ 95%. After filtering, 658,886 variants remained for PRS construction. The SA-PRS was derived using GWAS summary statistics from a large meta-analysis of SA in individuals of European genetic ancestry [25]. The originally published Psychiatric Genomics Consortium (PGC) GWAS meta-analysis included 15 cohorts, one of which was the UK Biobank. To avoid sample overlap between the discovery GWAS and the UK Biobank target sample used in the present study, and consistent with previous studies [13], we utilized a re-generated version of the GWAS summary statistics in which the UK Biobank cohort was explicitly excluded. This resulted in a new GWAS meta-analysis based on the remaining 14 independent cohorts, comprising 33,353 SA cases and 444,626 controls.* *The GWAS summary statistics used in the present study were obtained directly from the PGC through the Suicide Attempt Data Access Portal (https://pgc.unc.edu/for-researchers/data-access-committee/data-access-portal/) [accessed: 28/04/2025]). The SA-PRS was constructed using PRS-CS [32], a Bayesian polygenic prediction method that estimates SNP effect sizes by integrating GWAS summary statistics with external linkage disequilibrium (LD) reference panels. Given that both the discovery GWAS and the target dataset consisted of individuals of European genetic ancestry, LD was estimated using the European reference panel from the 1000 Genomes Project [33]. All eligible SNPs were combined to calculate the PRS for each participant using PLINK software. Higher PRS values were associated with increased genetic risk for SA. We standardized the PRS and classified participants into low, intermediate, and high genetic risk groups according to tertiles.*”

**Response Table 7.** **The 14 cohorts included in the SA GWAS meta-analysis**

| **Cohort** | **SA Cases** | **Controls** |
| --- | --- | --- |
| Army STARRS | 670 | 10,637 |
| Australian Genetics of Depression Study | 2,792 | 20,193 |
| Columbia University | 577 | 1,233 |
| GISS Ukraine | 660 | 660 |
| German Borderline Genomics Consortium | 481 | 1,653 |
| iPSYCH | 7,003 | 52,227 |
| Janssen | 255 | 1,684 |
| Million Veteran Program | 9,196 | 287,370 |
| PGC Bipolar Disorder | 3,214 | 17,642 |
| PGC Eating Disorders | 170 | 5,070 |
| PGC Major Depressive Disorder | 1,528 | 16,626 |
| PGC Schizophrenia | 1,640 | 7,112 |
| University of Utah | 4,692 | 20,702 |
| Yale–Penn | 475 | 1,817 |
| Total | 33,353 | 444,626 |

**6. Hazard model analysis was conducted using SA information available from electronic health records. However, in the methods, the authors mentioned that self-reported information was also used to define SA cases at baseline. The authors should clarify this point to clearly explain how these different data sources were used.**

**Response:** We thank you for highlighting this point and agree that further clarification was needed. We have revised the Methods section to clearly distinguish the data sources used for defining SA at baseline and during follow-up. Specifically, incident SA cases during follow-up were ascertained exclusively using hospital inpatient records, based on ICD-10 diagnostic codes recorded in linked electronic health records. Self-reported information was not used to define SA events during follow-up. In contrast, baseline SA cases were identified using both hospital inpatient records and participant self-reports collected at baseline. Individuals with evidence of prior SA from either source were excluded from the analytic cohort to ensure that all SA events analyzed in the hazard models represented incident cases occurring after baseline. We have clarified this distinction in the Methods section of the revised manuscript.

**Methods section (Page 9, Lines 157-161):** “*Incident SA cases during follow-up were ascertained exclusively from hospital inpatient records, based on diagnoses recorded under the International Classification of Diseases, 10th Revision (ICD-10) coding system (X60-X84, Y870) [25]. Self-reported information was not used to define SA events during follow-up.*”

**Methods section (Page 9, Lines 163-167):** “*Baseline SA cases were identified using both hospital inpatient records and self-reported information collected at baseline (Data-Field 20002, code 1290). Participants with evidence of SA from either source prior to baseline were excluded from the analysis to ensure that all SA events analyzed in the study represented incident cases occurring after baseline.*”

**7. In the main text, the authors should expand the description of the sensitivity analyses in the main text to guide the readers through the supplemental material describing them.**

**Response:** Thank you for this helpful suggestion. Now, we have expanded and clarified the description of the sensitivity analyses in the main text. Specifically, we now provide a structured overview of the rationale, analytical strategy, and purpose of each sensitivity analysis, with explicit references to the corresponding supplementary tables. This revision is intended to guide readers through the supplemental material more effectively and to demonstrate the robustness of our findings across multiple analytical scenarios. The revised text has been added to both the Methods and Results sections.

**Methods section (Pages 14 and 15, Lines 279-296):** “*To assess the robustness of the primary findings, we conducted a series of pre-specified sensitivity analyses addressing potential sources of bias and confounding. First, to mitigate potential reverse causation, we excluded SA cases occurring within the first two years of follow-up and re-estimated the associations. Second, to account for missing covariate data, we repeated the association analyses using imputed data, processing missing covariates via multiple imputation techniques. Specifically, we generated five imputed datasets with 20 iterations to ensure convergence and stability of the imputation process and pooled the estimates from Cox proportional hazards regression models across imputed datasets using Rubin’s rules [43]. Third, to minimize confounding from severe pre-existing conditions and socioeconomic disadvantage, an additional sensitivity analysis was performed by excluding participants with baseline CVD or cancer, as well as those with higher socioeconomic deprivation. Fourth, to further address potential residual confounding by comorbidities, we additionally adjusted for the Charlson Comorbidity Index (CCI) (S6 Table) [44]. We also calculated E-values to quantify the minimum strength of association that an unmeasured confounder would need to have with both frailty status and SA to fully explain the observed associations [45]. Fifth, given that death could preclude the occurrence of SA during follow-up, we conducted a competing risk analysis using the Fine-Gray subdistribution hazards model, treating death as a competing event. The detailed results of these sensitivity analyses are presented in the supplementary materials.*”

**Results section (Pages 18 and 19, Lines 364-378):** “*Overall, the results of the sensitivity analyses were consistent with the primary findings, supporting the robustness of the observed association between frailty status and the risk of SA. Excluding SA cases that occurred within the first two years of follow-up did not materially change the results (S13 Table). Similarly, analyses based on multiply imputed datasets yielded effect estimates comparable to those from* *the complete-case analysis (S14 Table). When participants with baseline CVD or cancer and those with higher socioeconomic deprivation were excluded, the associations between frailty status and SA risk remained largely unchanged in magnitude and direction (S15 Table). Additional adjustment for the CCI also did not materially alter the results (S16 Table), suggesting that residual confounding by comorbidity burden was unlikely to fully explain the observed associations. E-value analyses indicated substantial robustness to unmeasured confounding, as an unmeasured confounder would need to be associated with both frailty status and SA by a risk ratio of approximately 3.0 to fully account for the observed associations (S17 Table). Furthermore, results from the competing risk analysis accounting for death as a competing event were consistent with those from the primary Cox proportional hazards models (S18 Table).*”

**Reviewer #4: This study examined the association between frailty (measured using an index composed of a series of indicators) and suicide attempt in the UK Biobank. More specifically, it merged UK Biobank survey data with administrative data on suicide attempt prospectively collected over a median follow-up of 13 years. The study further analyzed potential biological mediation mechanisms using blood biomarkers. Mendelian randomization was used to strengthen causal inference regarding the association between frailty and suicide attempt. Overall, the paper is well written and methodologically sound, and, in my opinion, it represents an important addition to the evidence on risk factors for suicide attempt. The authors appropriately identify methodological limitations.**

**My main suggestions are:**

**1. The Introduction could be reworked to provide more substantive justification for the analyses conducted in this study. In several instances, the rationale relies on the fact that a topic "has not been investigated before," which, on its own, is not a sufficiently strong justification.**

**Response:** We thank you for this insightful comment and agree that a research gap alone does not constitute a sufficiently strong rationale. Now, we have substantially reworked the Introduction to strengthen the conceptual, clinical, and methodological justification for the analyses conducted in this study. Specifically, we now frame the study rationale around several complementary considerations rather than novelty alone. **First**, we emphasize the substantial public health relevance of suicide attempt (SA), particularly in older adults, by highlighting their strong association with suicide mortality and the higher transition rate from SA to suicide death in later life [19, 20]. This framing underscores the importance of identifying age-related and potentially modifiable risk factors for SA, beyond established psychiatric and psychosocial determinants. **Second**, we provide a clearer theoretical rationale linking aging-related vulnerability to suicide risk. We elaborate on how age-related declines in physiological resilience and stress tolerance may contribute to susceptibility to SA, and position physical frailty as a clinically meaningful and modifiable manifestation of this vulnerability. We further outline biologically and clinically plausible mechanisms, spanning functional impairment, loss of independence, inflammation, endocrine and metabolic dysregulation, and reduced stress-coping capacity, through which frailty may influence suicide-related outcomes [2, 21-27]. This conceptual framework grounds the analyses in established aging and suicide biology, rather than solely in unexplored associations. **Third**, we clarify the methodological motivation for focusing on the frailty phenotype (FP). Although one prior quantitative study has examined frailty and SA using a cumulative-deficit frailty index [27], we highlight the limited clinical feasibility of this approach and the importance of evaluating the FP, which is widely used, intuitive, and potentially actionable in clinical and public health settings. **Moreover**, we strengthen the rationale by noting that individual components of the FP (e.g., fatigue, muscle weakness, slow gait, weight loss, and low physical activity) have each been independently associated with suicide-related outcomes[28-32], providing indirect but compelling support for examining the FP as an integrated construct. **Finally**, we justify the use of complementary analytic approaches. The prospective cohort design allows us to establish temporal associations between frailty and SA, while Mendelian randomization offers genetic support that can help mitigate residual confounding and reverse causation. In addition, incorporating polygenic risk scores and biomarker analyses enables us to explore effect modification and potential biological pathways, thereby extending the study beyond association testing toward mechanistic insight. Taken together, these revisions strengthen the Introduction by articulating a multifaceted rationale for the study rather than relying primarily on the absence of prior research. The revised text has been added to both the Introduction sections.

**Introduction section (Page 4, Lines 57-64):** “*Although existing research on SA has largely focused on psychiatric disorders and psychosocial determinants, emerging evidence has suggested that aging-related vulnerability may play an important role in suicide risk [1, 3]. Suicide death rates increase with age [4], and SA among older adults is more likely to be fatal [3], suggesting that age-related declines in physiological resilience and stress tolerance may contribute to greater vulnerability to suicidal behavior.*”

**Introduction section (Page 5, Lines 72-80):** “*Conceptually, frailty may heighten susceptibility to suicide-related outcomes through multiple interrelated pathways, including functional impairment, loss of independence, chronic inflammation, endocrine dysregulation, metabolic disturbances, and a reduced capacity to cope with psychological and physical stressors [5, 6, 10-15]. In line with this perspective, a recent narrative review has highlighted frailty as a potentially important factor in late-life suicidal ideation (SI) and SA, although the existing evidence is largely qualitative in nature [16]. Despite this biological and clinical plausibility, empirical evidence linking frailty to suicide-related outcomes remains limited. Existing quantitative studies on this topic have predominantly focused on SI, consistently reporting positive associations between frailty and SI [17-19].*”

**Introduction section (Pages 5 and 6, Lines 87-100):** “*Unlike the complex FI, the frailty phenotype (FP), which captures frailty based on five components [fatigue, weakness, slowness, weight loss, and low physical activity (PA)], is a widely adopted, clinically intuitive, and modifiable measure of physical frailty [5, 20]. However, the association between FP-measured physical frailty and SA remains unexplored. Importantly, previous studies have identified individual components of the FP associated with suicide-related outcomes, including fatigue [21], reduced grip strength [17, 22], slow walking speed [17], weight loss [23], and low PA [24]. These observations support a biologically and clinically plausible hypothesis that FP-measured physical frailty may be associated with increased risk of SA. Hence, a comprehensive study integrating a prospective cohort design with Mendelian randomization (MR) is warranted to better characterize this association.*”

**Introduction section (Page 7, Lines 114-118):** “*Against this background, the present study aimed to examine the association between FP-measured physical frailty and the risk of SA in a large prospective cohort from the UK Biobank. Further, we employed two-sample MR analyses as a complementary approach to explore whether the observed association shows genetic support consistent with the cohort findings.*”

**2. Using multivariable Mendelian randomization to test the mediating role of the identified biomarker could be a valuable addition to the paper and would help address the limitation related to the simultaneous measurement of frailty and biomarkers.**

**Response:** We thank you for this insightful and constructive comment. We agree that Mendelian randomization (MR) can provide an important complementary approach to strengthen causal inference beyond observational mediation analyses, and that both two-step MR and multivariable MR can, in principle, be applied to mediation analyses [13, 14]. To address your concern regarding causality in the biomarker mediation analysis, we implemented a two-step Mendelian randomization approach within a two-sample MR framework, which has been widely recommended and applied to evaluate putative mediators in exposure-outcome relationships [13, 14, 33, 34]. Conceptually, two-step MR is analogous to the *product-of-coefficients* method in conventional mediation analysis, whereas multivariable MR estimates the indirect effect by subtracting the direct effect from the total effect, similar to the *difference-in-coefficients* approach [13]. Specifically, in the first step of the two-step MR, we assessed the causal effect of genetically predicted physical frailty on each candidate biomarker. In the second step, we examined the causal effect of each biomarker on suicide attempt (SA) risk using independent genetic instruments. Evidence for a causal mediating role was considered present only if both steps demonstrated statistically significant associations in the expected direction.

Although our observational mediation analyses suggested partial mediation by biomarkers related to liver function, metabolism, immunity, and inflammation, the two-step MR analyses did not identify any biomarker that was statistically significant in both steps. As a result, no biomarker met the criteria for a causal mediator within the MR framework. Given the absence of evidence for causal mediation at the single-biomarker level, we did not proceed with multivariable MR, as it would not provide additional informative value in the absence of any candidate mediators supported by the two-step MR analysis. Our choice of the two-step MR approach is also methodologically consistent with the observational mediation strategy used in this study, which relied on two regression models to screen potential mediators. The discrepancy between the observational mediation analysis and the MR findings may be attributable to several factors. First, genetic instruments for circulating biomarkers typically explain only a small proportion of phenotypic variance, which substantially limits statistical power in MR analyses, particularly when the outcome of interest, SA, is relatively rare and etiologically heterogeneous. Second, the biomarkers identified in the observational analyses may predominantly reflect downstream consequences of frailty or correlated biological processes, rather than functioning as direct causal mediators in the pathway linking frailty to suicide risk. We have revised the manuscript accordingly to clearly reflect these points and to adopt a cautious interpretation of the biomarker findings. The relevant revisions are summarized below:

**Abstract section (Page 3, Lines 24-26):** “*Biomarkers related to liver function, metabolism, immunity, and inflammation may have partially explained this association, accounting for a collective 14.15% (95% CI: 8.94%-19.74%) of the total effect, although MR analyses did not support causal mediating effects.*”

**Methods section (Page 14, Lines 271-278):** “*To further investigate whether the biomarkers identified in the observational mediation analysis lie on the causal pathway linking physical frailty to SA, we conducted two-step MR analyses [42]. In the first step, genetic instruments for physical frailty were used to estimate the causal effect of frailty on each candidate biomarker. In the second step, genetic instruments for the corresponding biomarker were applied to assess its causal effect on SA. Details regarding the selection of genetic instruments, quality control procedures, and MR estimation methods are provided in the S1 Methods. Evidence for causal mediating roles was considered present only if both steps demonstrated statistically significant associations in the expected direction.”*

**Results section (Page 18, Lines 356-360):** “*Each of these biomarkers demonstrated a statistically significant indirect effect in the observational mediation analyses (all P_FDR_ < 0.05), with the PM ranging from 0.23% (95% CI: 0.06%-0.41%) to 3.53% (95% CI: 1.72%-5.23%) (Figure 3).*”

**Results section (Page 18, Lines 361 and 362):** “*However, subsequent two-step MR analyses did not provide statistically significant evidence supporting a causal mediating role for these biomarkers (S12 Table).*”

**Discussion section (Page 19, Lines 390-392):** “*Furthermore, we found that approximately 14.2% of the frailty-SA association could be statistically explained by biomarkers related to liver function, metabolism, immune function, and inflammation.*”

**Discussion section (Page 24, Lines 486-494):** “*Notably, subsequent two-step MR analyses did not provide statistically significant evidence supporting a causal mediating role for these biomarkers. The discrepancy between the observational mediation and MR findings may reflect several factors. First, genetic instruments for circulating biomarkers typically explain limited phenotypic variance, substantially reducing statistical power in MR analyses, particularly for rare and etiologically heterogeneous outcomes such as SA. Second, the biomarkers identified in observational analyses may primarily represent downstream correlates of frailty or shared biological processes, rather than direct causal mediators in the frailty-SA pathway. Therefore, these findings should be interpreted as indicating potential biological pathways rather than confirmed causal intermediates.*”

**Conclusion section (Page 26, Lines 537-540):** “*Furthermore, a range of biomarkers related to liver function, metabolism, immune function, and inflammation statistically explained a modest proportion of this association, highlighting potential biological pathways accompanying frailty. However, these biomarkers should be interpreted as potential contributors rather than confirmed causal mediators.*”

**3. Frailty can be associated with important psychological consequences, including depressive symptoms, anxiety, social isolation, and feelings of hopelessness or perceived burdensomeness, which may influence individuals' risk of suicide attempt. These factors deserve to be mentioned and discussed as alternative or complementary mechanisms underlying the associations observed in this study. Changes in biomarkers may also be related to such psychological states.**

**Response:** Thank you for this insightful and important comment. We fully agree that frailty is not only a biological aging-related syndrome but may also have profound psychological and social consequences, including depressive symptoms, anxiety, social isolation, hopelessness, and perceived burdensomeness, all of which are relevant to SA risk. These psychosocial factors may act as complementary pathways linking frailty to SA and may also interact with biological alterations observed in frailty. According to your suggestions, we have revised the Discussion to explicitly acknowledge and discuss these psychological and social mechanisms as plausible contributors to the frailty-SA association. We also added text highlighting that the identified biomarker changes may partly reflect psychological distress and stress-related physiological responses, rather than representing purely biological pathways. These revisions strengthen the interpretation of our findings by integrating psychosocial and biological perspectives. The revised text has been added to the Discussion section of the revised manuscript.

**Discussion section (Page 22, Lines 451-460):** “*One plausible explanation involves psychosocial mechanisms. Specifically, frailty is often accompanied by substantial psychological and social consequences, including depressive symptoms, anxiety, social isolation, hopelessness, and perceived burdensomeness [6, 9, 18]. These factors have been identified as proximal risk correlates of suicidal behavior [1, 3]. These psychosocial sequelae may accumulate as frailty progresses, intensifying emotional distress and diminishing perceived social connectedness and coping resources [50]. In line with this, we observed that frailty also significantly impacts SI, and that SI plays a key role in the frailty-SA relationship. Approximately one-third of the frailty-SA association could be explained by SI (S22 Table). Thus, in addition to physical vulnerability, frailty may contribute to SA through both psychosocial adversity and emotional distress that facilitate the transition from SI to SA.*”

**Discussion section (Page 23, Lines 463-468):** “*Notably, these alterations in biomarkers may not stem purely from biological factors. Psychological distress and chronic stress exposure, which are common among frail individuals [51], can influence neuroendocrine regulation and immune functioning [52, 53], thereby shaping metabolic, inflammatory, and hematological profiles. As such, biomarker dysregulation may reflect an interplay between biological aging processes and stress-related psychobiological responses, both of which could contribute to elevated suicide risk.*”

**Other points:**

**1. Page 3: "SA, one of the strongest risk factor for suicide deaths, is preventable." Suggest rephrasing as "may be preventable."**

**Response:** Thank you for this helpful suggestion. We agree that the original statement may have been overly definitive. We have revised the sentence to use more cautious wording. The following revisions can be found in the Introduction section.

**Introduction section (Page 4, Line 55):** *“SA, one of the strongest risk factors for suicide deaths, may be preventable [1].”*

**2. Same page: "Suicide attempt increase with age… risk factor for SA." The logic of this sentence is unclear and may benefit from rephrasing for clarity.**

**Response:** Thank you for pointing this out. We agree that the logic of the original sentence could be clearer. We have revised this part to improve readability. The following revisions can be found in the Introduction section.

**Introduction section (Page 4, Lines 61-64):** *“Suicide death rates increase with age [4], and SA among older adults is more likely to be fatal [3], suggesting that age-related declines in physiological resilience and stress tolerance may contribute to greater vulnerability to suicidal behavior.”*

**References:**

1.	Gao PY, Fu Y, He XY, Zhang DD, Chi HC, Huang YM, et al. Genetic mechanisms, brain structures, and peripheral biomarkers mediate the relationship between physical frailty and neuropsychiatric disorders. J Adv Res. 2025;doi:10.1016/j.jare.2025.06.046

2.	Zhang B, You J, Rolls ET, Wang X, Kang J, Li Y, et al. Identifying behaviour-related and physiological risk factors for suicide attempt in the UK Biobank. Nat Hum Behav. 2024;8:1784-97. doi:10.1038/s41562-024-01903-x

3.	Zhang Z, Xu H, Zhang R, Yan Y, Ling X, Meng Y, et al. Frailty and depressive symptoms in relation to cardiovascular disease risk in middle-aged and older adults. Nature communications. 2025;16:6008. doi:10.1038/s41467-025-61089-2

4.	Liu F, Peng Y, Wang P, Qiao Y, Si C, Wang X, et al. Associations of physical frailty with incidence and mortality of overall and site-specific cancers: A prospective cohort study from UK biobank. Preventive medicine. 2023;177:107742. doi:10.1016/j.ypmed.2023.107742

5.	Petermann-Rocha F, Lyall DM, Gray SR, Esteban-Cornejo I, Quinn TJ, Ho FK, et al. Associations between physical frailty and dementia incidence: a prospective study from UK Biobank. The lancet Healthy longevity. 2020;1:e58-e68. doi:10.1016/s2666-7568(20)30007-6

6.	Geng T, Zhu K, Lu Q, Wan Z, Chen X, Liu L, et al. Healthy lifestyle behaviors, mediating biomarkers, and risk of microvascular complications among individuals with type 2 diabetes: A cohort study. PLoS medicine. 2023;20:e1004135. doi:10.1371/journal.pmed.1004135

7.	Wang C, Zhang X, Li B, Mu D. A study of factors impacting disease based on the Charlson Comorbidity Index in UK Biobank. Frontiers in public health. 2022;10:1050129. doi:10.3389/fpubh.2022.1050129

8.	Ganna A, Ingelsson E. 5 year mortality predictors in 498,103 UK Biobank participants: a prospective population-based study. Lancet (London, England). 2015;386:533-40. doi:10.1016/s0140-6736(15)60175-1

9.	Gaster T, Eggertsen CM, Støvring H, Ehrenstein V, Petersen I. Quantifying the impact of unmeasured confounding in observational studies with the E value. BMJ Med. 2023;2:e000366. doi:10.1136/bmjmed-2022-000366

10.	Jia Y, Shi M, Yang P, Wang R, Sun L, Wang Y, et al. Associations of computer gaming with incident dementia, cognitive functions, and brain structure: a prospective cohort study and Mendelian randomization analysis. Alzheimers Res Ther. 2024;16:131. doi:10.1186/s13195-024-01496-7

11.	Song Y, Zhu C, Shi B, Song C, Cui K, Chang Z, et al. Social isolation, loneliness, and incident type 2 diabetes mellitus: results from two large prospective cohorts in Europe and East Asia and Mendelian randomization. EClinicalMedicine. 2023;64:102236. doi:10.1016/j.eclinm.2023.102236

12.	Lin F, Shi Y, Song W, Weng Y, Zou X, Chen X, et al. Daytime napping and the incidence of Parkinson's disease: a prospective cohort study with Mendelian randomization. BMC medicine. 2024;22:326. doi:10.1186/s12916-024-03497-7

13.	Carter AR, Sanderson E, Hammerton G, Richmond RC, Davey Smith G, Heron J, et al. Mendelian randomisation for mediation analysis: current methods and challenges for implementation. European journal of epidemiology. 2021;36:465-78. doi:10.1007/s10654-021-00757-1

14.	Relton CL, Davey Smith G. Two-step epigenetic Mendelian randomization: a strategy for establishing the causal role of epigenetic processes in pathways to disease. International journal of epidemiology. 2012;41:161-76. doi:10.1093/ije/dyr233

15.	Skrivankova VW, Richmond RC, Woolf BAR, Yarmolinsky J, Davies NM, Swanson SA, et al. Strengthening the Reporting of Observational Studies in Epidemiology Using Mendelian Randomization: The STROBE-MR Statement. Jama. 2021;326:1614-21. doi:10.1001/jama.2021.18236

16.	National Academies of Sciences E, Medicine, Division of B, Social S, Education, Health, et al. The National Academies Collection: Reports funded by National Institutes of Health. In: Washington (DC): National Academies Press (US)

Copyright 2023 by the National Academy of Sciences. All rights reserved.; 2023.

17.	Karczewski KJ, Gupta R, Kanai M, Lu W, Tsuo K, Wang Y, et al. Pan-UK Biobank genome-wide association analyses enhance discovery and resolution of ancestry-enriched effects. Nature genetics. 2025;57:2408-17. doi:10.1038/s41588-025-02335-7

18.	Docherty AR, Mullins N, Ashley-Koch AE, Qin X, Coleman JRI, Shabalin A, et al. GWAS Meta-Analysis of Suicide Attempt: Identification of 12 Genome-Wide Significant Loci and Implication of Genetic Risks for Specific Health Factors. The American journal of psychiatry. 2023;180:723-38. doi:10.1176/appi.ajp.21121266

19.	Beghi M, Butera E, Cerri CG, Cornaggia CM, Febbo F, Mollica A, et al. Suicidal behaviour in older age: A systematic review of risk factors associated to suicide attempt and completed suicides. Neuroscience and biobehavioral reviews. 2021;127:193-211. doi:10.1016/j.neubiorev.2021.04.011

20.	Global, regional, and national age-sex-specific mortality for 282 causes of death in 195 countries and territories, 1980-2017: a systematic analysis for the Global Burden of Disease Study 2017. Lancet (London, England). 2018;392:1736-88. doi:10.1016/s0140-6736(18)32203-7

21.	Dent E, Martin FC, Bergman H, Woo J, Romero-Ortuno R, Walston JD. Management of frailty: opportunities, challenges, and future directions. Lancet (London, England). 2019;394:1376-86. doi:10.1016/s0140-6736(19)31785-4

22.	Hoogendijk EO, Afilalo J, Ensrud KE, Kowal P, Onder G, Fried LP. Frailty: implications for clinical practice and public health. Lancet (London, England). 2019;394:1365-75. doi:10.1016/s0140-6736(19)31786-6

23.	Soysal P, Stubbs B, Lucato P, Luchini C, Solmi M, Peluso R, et al. Inflammation and frailty in the elderly: A systematic review and meta-analysis. Ageing research reviews. 2016;31:1-8. doi:10.1016/j.arr.2016.08.006

24.	Clegg A, Hassan-Smith Z. Frailty and the endocrine system. The lancet Diabetes & endocrinology. 2018;6:743-52. doi:10.1016/s2213-8587(18)30110-4

25.	Mishra M, Wu J, Kane AE, Howlett SE. The intersection of frailty and metabolism. Cell Metab. 2024;36:893-911. doi:10.1016/j.cmet.2024.03.012

26.	Abou Chahla MN, Khalil MI, Comai S, Brundin L, Erhardt S, Guillemin GJ. Biological Factors Underpinning Suicidal Behaviour: An Update. Brain Sci. 2023;13:doi:10.3390/brainsci13030505

27.	Kuffel RL, Morin RT, Covinsky KE, Boscardin WJ, Lohman MC, Li Y, et al. Association of Frailty With Risk of Suicide Attempt in a National Cohort of US Veterans Aged 65 Years or Older. JAMA psychiatry. 2023;80:287-95. doi:10.1001/jamapsychiatry.2022.5144

28.	Luz LS, Cassenote AJF, Valente EP, Mariani I, Lazzerini M, Lima C, et al. Mental health of Brazilian physicians: a nationwide crosssectional study to investigate factors associated with the prevalence of suicide plans and attempt. Braz J Psychiatry. 2024;46:e20233393. doi:10.47626/1516-4446-2023-3393

29.	Smith L, López Sánchez GF, Soysal P, Veronese N, Gibson P, Pizzol D, et al. Association of handgrip strength with suicidal ideation among adults aged ≥50 years from low- and middle-income countries. Suicide Life Threat Behav. 2024;54:606-14. doi:10.1111/sltb.13071

30.	Bickford D, Morin RT, Woodworth C, Verduzco E, Khan M, Burns E, et al. The relationship of frailty and disability with suicidal ideation in late life depression. Aging & mental health. 2021;25:439-44. doi:10.1080/13607863.2019.1698514

31.	Otsuka T, Tomata Y, Sugawara Y, Tsuji I. Association between weight loss since the age of 20 years and the risk of suicide death: A population-based cohort study. Journal of affective disorders. 2021;292:746-50. doi:10.1016/j.jad.2021.05.127

32.	Fabiano N, Gupta A, Wong S, Tran J, Mohammad IY, Bal S, et al. Physical activity, suicidal ideation, suicide attempt and death among individuals with mental or other medical disorders: A systematic review of observational studies. Neuroscience and biobehavioral reviews. 2024;158:105547. doi:10.1016/j.neubiorev.2024.105547

33.	Zhang Z, Hu X, Tao W, Ma R, Zheng Y, Fang X, et al. Host-gut microbial metabolic crosstalk in postpartum depression: A multiomics insight linking blood metabolites to epigenetic modulation. Journal of affective disorders. 2026;400:121166. doi:10.1016/j.jad.2026.121166

34.	Deng J, Pan L, Huang J, Yang J, Wang M, Wang T, et al. Modifiable Dietary Habits, Inflammatory Mediation, and Senile Cataract: Unraveling Causality via Mendelian Randomization. Food Sci Nutr. 2025;13:e71366. doi:10.1002/fsn3.71366

**Respond to General editorial requests**

*** We ask every co-author listed on the manuscript to fill in a contributing author statement, making sure to declare all competing interests. If any of the co-authors have not filled in the statement, we will remind them to do so when the paper is revised. If all statements are not completed in a timely fashion this could hold up the re-review process. If new competing interests are declared later in the revision process, this may also hold up the submission. Should there be a problem getting one of your co-authors to fill in a statement we will be in contact. Please do not add or remove authors without first discussing this with the handling editor. You can see our competing interests policy here: http://journals.plos.org/plosmedicine/s/competing-interests.**

**Response:** Thank you for the information regarding the contributing author statements and competing interests. We confirm that all authors declare that they have no competing interests, and this has been clearly stated in the manuscript as: “*All authors declare that they have no competing interests*.” However, we would like to note that none of the co-authors listed on the manuscript have received an email requesting them to fill in a contributing author statement. Please let us know if these invitations will be sent at a later stage, or if any action is required from us to facilitate this process. We fully understand the importance of completing all required statements in a timely manner and are happy to follow any guidance you provide to avoid delays during revision and re-review.

*** Please upload any figures associated with your paper as individual TIF or EPS files with 300dpi resolution at resubmission; please read our figure guidelines for more information on our requirements: http://journals.plos.org/plosmedicine/s/figures. While revising your submission, we strongly recommend that you use PLOS's NAAS tool (https://ngplosjournals.pagemajik.ai/artanalysis) to test your figure files. NAAS can convert your figure files to the TIFF file type and meet basic requirements (such as print size, resolution), or provide you with a report on issues that do not meet our requirements and that NAAS cannot fix.**

**After uploading your figures to PLOS's NAAS tool - https://ngplosjournals.pagemajik.ai/artanalysis, NAAS will process the files provided and display the results in the "Uploaded Files" section of the page as the processing is complete. If the uploaded figures meet our requirements (or NAAS is able to fix the files to meet our requirements), the figure will be marked as "fixed" above. If NAAS is unable to fix the files, a red "failed" label will appear above. When NAAS has confirmed that the figure files meet our requirements, please download the file via the download option, and include these NAAS processed figure files when submitting your revised manuscript.**

**Response:** Thank you for this guidance. All figures have been uploaded as individual TIF files with a resolution of 300 dpi, in accordance with the PLOS figure guidelines. All figure files have been checked and processed using PLOS’s NAAS tool. The figures included in the revised manuscript are the NAAS-processed files that meet PLOS requirements.

***Please ensure that the paper adheres to the PLOS Data Availability Policy (see http://journals.plos.org/plosmedicine/s/data-availability), which requires that all data underlying the study's findings be provided in a repository or as Supporting Information. For data residing with a third party, authors are required to provide instructions with contact information (web or email address) for obtaining the data. Please note that a study author cannot be the contact person for the data. PLOS journals do not allow statements supported by "data not shown" or "unpublished results." For such statements, authors must provide supporting data or cite public sources that include it.**

**Response:** Thank you for your guidance. The following statement has already been included in the manuscript.

**Data Availability Statement (Page 27, Lines 555-557):** “*Data are available in a public, open access repository. This research has been conducted using the UK Biobank Resource under Application Number 91536. The UK Biobank data are available on application to the UK Biobank (www.ukbiobank.ac.uk/) with access fees.*”

*** We expect all researchers with submissions to PLOS in which author-generated code underpins the findings in the manuscript to make all author-generated code available without restrictions upon publication of the work. In cases where code is central to the manuscript, we may require the code to be made available as a condition of publication. Authors are responsible for ensuring that the code is reusable and well documented. Please make any custom code available, either as part of your data deposition or as a supplementary file. Please add a sentence to your data availability statement regarding any code used in the study, e.g. "The code used in the analysis is available from Github [URL] and archived in Zenodo [DOI link]" Please review our guidelines at https://journals.plos.org/plosmedicine/s/materials-software-and-code-sharing and ensure that your code is shared in a way that follows best practice and facilitates reproducibility and reuse. Because Github depositions can be readily changed or deleted, we encourage you to make a permanent DOI'd copy (e.g. in Zenodo) and provide the URL.**

**Response:** Thank you for this comment. No custom, author-generated code was used in this study that underpins the findings of the manuscript. All analyses were conducted using standard, publicly available software. If further clarification is required, please let us know.

*** Please ensure that the study is reported according to the STROBE guideline and include the completed STROBE checklist as Supporting Information. When completing the checklist, please use section and paragraph numbers, rather than page numbers. Please add the following statement, or similar, to the Methods: "This study is reported as per STROBE guideline (S1 Checklist)."**

**Response:** Thank you for your guidance. The following statement has already been added to the Methods section in the manuscript.

**Methods section (Page 8, Lines 142-144):** “*The current study was reported according to the Strengthening the Reporting of Observational Studies in Epidemiology (STROBE) guideline (S1 Checklist).*”

**Methods section (Page 13, Lines 251 and 252):** “*The MR study followed the STROBE-MR guidelines (S2 Checklist) [39].*”

**FORMATTING - GENERAL**

***Abstract: Please structure your abstract using the PLOS Medicine headings (Background, Methods and Findings, Conclusions). Please combine the Methods and Findings sections into one section.**

**Response:** Thank you for this comment. The abstract has been revised to follow the PLOS Medicine structured format, using the headings Background, Methods and Findings, and Conclusions, with the Methods and Findings combined into a single section as requested.

**Abstract:** *“****Background:*** *While physical frailty is linked to psychiatric disorders, its association with suicide attempt (SA) risk is unclear. We aimed to investigate the prospective association of physical frailty with SA risk and the modifying and potential mediating roles of genetic risk and blood biomarkers.*

***Methods and findings:*** *This cohort study included 442,920 UK Biobank participants free of SA at baseline. SA events were extracted by linking hospital inpatient records. Physical frailty status was assessed using the five-component Fried phenotype and categorized as non-frail, pre-frail, or frail. Genetic risk for SA was estimated through polygenic risk scores and categorized into high, intermediate, and low risk levels. Cox proportional hazard models were conducted to calculate the hazard ratios (HRs) and 95% confidence intervals (CIs) for the association. Mendelian randomization (MR) analyses were utilized to examine the association between genetically determined physical frailty and SA. Mediation analyses were performed to explore potential biological pathways involving circulating biomarkers. During a mean follow-up of 13.6 years, 1,518 (0.3%) individuals developed SA. Compared to non-frail individuals, the HRs (95% CIs) for SA among those with pre-frailty were 1.61 (95% CI [1.44-1.80]; P < 0.001) and frailty were 2.16 (95% CI [1.78-2.61]; P < 0.001). Genetically predicted frailty was also positively associated with SA (odds ratio = 2.06, 95% CI [1.21-3.52]; P = 0.008). Except for low physical activity, all frailty components were significantly associated with an increased risk of SA, with HRs ranging from 1.16 to 1.62 (all P < 0.05). The additive interaction of physical frailty and genetic risk increased the risk of SA, with the highest risk observed among frail individuals at high genetic risk (HR=3.09, 95% CI [2.30-4.17]; P < 0.001), whereas no significant multiplicative interactions were detected. Biomarkers related to liver function, metabolism, immunity, and inflammation may have partially explained this association, accounting for a collective 14.15% (95% CI: 8.94%-19.74%) of the total effect, although MR analyses did not support causal mediating effects.*

***Conclusions:*** *Pre-frail and frail states were associated with an increased risk of SA, especially among individuals with high genetic risk. Incorporating frailty assessment and management into primary prevention strategies may have implications for SA prevention.”*

***At this stage, we ask that you include a short, non-technical Author Summary of your research to make findings accessible to a wide audience that includes both scientists and non-scientists. The Author Summary should immediately follow the Abstract in your revised manuscript. This text is subject to editorial change and should be distinct from the scientific abstract. Ideally each sub-heading should contain 2-3 single sentence, concise bullet points containing the most salient points from your study. In the final bullet point of 'What Do These Findings Mean?', please include the main limitations of the study in non-technical language. Please see our author guidelines for more information:https://journals.plos.org/plosmedicine/s/revising-your-manuscript#loc-author summary.**

**Response:** Thank you for this comment. A non-technical Author Summary has been added immediately following the Abstract in the revised manuscript.

**Author Summary**

**“*Why was this study done?***

- *Suicide attempt (SA) are a major public health concern, and identifying risk factors may help improve early prevention.*
- *Physical frailty has been linked to poor mental health, but its relationship with SA remains unclear.*
- *This study aimed to examine whether physical frailty is associated with the risk of SA and whether genetic risk and blood biomarkers influence this association.*

***What did the researchers do and find?***

- *In this cohort study of more than 440,000 participants who had no history of SA at baseline, those with frailty had a substantially higher risk of SA compared with non-frail individuals, and this association was also supported by genetic analyses.*
- *Participants who were both physically frail and at high genetic risk had the highest risk of SA, and blood markers related to metabolism, liver function, inflammation, and immune response may partly explain this relationship.*

***What do these findings mean?***

- *Physical frailty may be an important and previously under-recognized risk factor for SA, particularly among individuals with higher genetic risk.*
- *Assessing and managing frailty could help identify individuals at higher risk and inform suicide prevention strategies.*”

***Please express the main results with 95% CIs as well as p values. When reporting p values please report as p<0.001 and where higher as the exact p value p=0.002, for example. Throughout, suggest reporting statistical information as follows to improve clarity for the reader "22% (95% CI [13%,28%]; p</=)". Please be sure to define all numerical values at first use.**

**Response:** Thank you for this comment. All main results are now reported with 95% confidence intervals and appropriately formatted p values, and all numerical values are defined at first use.

***Please include page numbers and line numbers in the manuscript file. Use continuous line numbers (do not restart the numbering on each page).**

**Response:** Thank you for this comment. Page numbers and continuous line numbers have been added throughout the manuscript.

***Please cite the reference numbers in square brackets. Citations should precede punctuation.**

**Response:** Thank you for this comment. All citations have been reformatted to use square brackets and placed before punctuation.

**FIGURES AND TABLES**

***Please provide titles and legends for all figures and tables (including those in Supporting Information files).**

**Response:** Thank you for your feedback. Titles and legends have been added to all figures and tables, including those in the Supporting Information files, as requested.

***Please define all abbreviations used in each figure/table (including those in Supporting Information files).**

**Response:** Thank you for the feedback. The manuscript has been carefully reviewed, and all abbreviations used in each figure and table, including those in the Supporting Information files, have been defined.

***Please consider avoiding the use of red and green in order to make your figure more accessible to those with color blindness.**

**Response:** Thank you for the suggestion. Red and green have not been used to distinguish content in the main figures in the revised manuscript.

**SUPPLEMENTARY MATERIAL**

***Please note that supplementary material will be posted as supplied by the authors. Therefore, please amend it according to the relevant comments outlined here.**

**Response:** Thank you for your feedback. The supplementary material has been amended according to the relevant comments as outlined.

***Please cite your Supporting Information as outlined here: https://journals.plos.org/plosmedicine/s/supporting-information**

**Response:** Thank you for your feedback. The Supporting Information citations have been checked and are in compliance with the guidelines.

**REFERENCES**

***PLOS uses the numbered citation (citation-sequence) method and first six authors, et al.**

**Response:** Thank you for your feedback. The references have been revised to follow the PLOS citation-sequence method, with the first six authors listed followed by “et al.” as required.

***Please ensure that journal name abbreviations match those found in the National Center for Biotechnology Information (NCBI) databases (http://www.ncbi.nlm.nih.gov/nlmcatalog/journals), and are appropriately formatted and capitalised.**

**Response:** Thank you for your feedback. The journal name abbreviations have been checked and are consistent with those found in the National Center for Biotechnology Information (NCBI) databases. They are appropriately formatted and capitalized.

***Where website addresses are cited, please include the complete URL and specify the date of access (e.g. [accessed: 12/06/2023]).**

**Response:** Thank you for this comment. All website addresses cited in the manuscript have been revised to include the complete URL and the date of access, in accordance with the journal’s requirements.

***Please also see https://journals.plos.org/plosmedicine/s/submission-guidelines#loc-references for further details on reference formatting.**

**Response:** Thank you for your feedback. We have checked the format of the references.

**OBSERVATIONAL STUDIES**

*** Abstract: Please include the study design, population and setting, number of participants, years during which the study took place (enrollment and follow up), length of follow up, and main outcome measures.**

**Response:** Thank you for your guidance. The required details have been included in the abstract, as indicated by the red markings.

**Abstract:** “**Background:** While physical frailty is linked to psychiatric disorders, its association with suicide attempt (SA) risk is unclear. We aimed to investigate the prospective association of physical frailty with SA risk and the modifying and mediating roles of genetic risk and blood biomarkers. **Methods and findings:** *This cohort study included 442,920 UK Biobank participants free of SA at baseline. SA events were extracted by linking hospital inpatient records. Physical frailty status was assessed using the five-component Fried phenotype, which classified individuals as non-frail, pre-frail, or frail. Genetic risk for SA was estimated through polygenic risk scores and further categorized into high, intermediate, and low risk levels. Cox proportional hazard models were conducted to calculate the hazard ratios (HRs) and 95% confidence intervals (CIs) for the association. Mendelian randomization (MR) analyses were utilized to examine the association between genetically determined physical frailty and SA. Mediation analyses evaluated the potential mediating effect of this association by a range of biomarkers. During a mean follow-up of 13.6 years, 1,518 (0.3%) individuals developed SA. Compared to non-frail individuals, the HRs (95% CIs) for SA among those with pre-frailty were 1.61 (95% CI [1.44-1.80]; P < 0.001) and frailty were 2.16 (95% CI [1.78-2.61]; P < 0.001). MR analyses suggested a positive association between genetically determined frailty and SA (odds ratio = 2.06, 95% CI [1.21-3.52]; P = 0.008). Except for low physical activity, all frailty components were significantly associated with an increased risk of SA, with HRs ranging from 1.16 to 1.62 (all P < 0.05). The additive interaction of physical frailty and genetic risk increased the risk of SA, with participants at high genetic risk and categorized as frail were at highest SA risk (HR=3.09, 95% CI [2.30-4.17]; P < 0.001). Across genetic risk groups, the reduced risk of SA was similar in non-frail participants compared with frail participants, with no significant multiplicative interactions observed. The biomarkers related to liver function, metabolism, immunity, and inflammation may have partially mediated this association, accounting for a collective 14.15% (95% CI: 8.94%-19.74%) of the total effect.*”

*** Please ensure that the study is reported according to the STROBE (or appropriate STOBE extension) guideline (available from: https://www.equator-network.org/reporting-guidelines/strobe) and include the completed STROBE (or STROBE extension) checklist as Supporting Information. Please add the following statement, or similar, to the Methods: "This study is reported as per the Strengthening the Reporting of Observational Studies in Epidemiology (STROBE) guideline (S1 Checklist)." When completing the checklist, please use section and paragraph numbers, rather than page numbers.**

**Response:** The necessary modifications have been made, and the corresponding content has been added.

**Methods (Page 8, Lines 142-144):** “*The current study was reported according to the Strengthening the Reporting of Observational Studies in Epidemiology (STROBE) guideline (S1 Checklist).*”

***[FOR POPULATION HEALTH/REGISTRY STUDIES] Please ensure that the study is reported according to the RECORD guideline (available from https://www.record-statement.org) and include the completed checklist as Supporting Information. Please add the following statement, or similar, to the Methods: "This study is reported as per the Reporting of Studies Conducted using Observational Routinely-Collected Data (RECORD) guideline (S1 Checklist)." When completing the checklist, please use section and paragraph numbers, rather than page numbers.**

**Response:** Not applicable.

***[FOR POPULATION HEALTH ESTIMATES] Please ensure that the study is reported according to the GATHER statement (available from https://www.equator-network.org/reporting-guidelines/gather-statement) and include the completed checklist as Supporting Information. Please add the following statement, or similar, to the Methods: "This study is reported as per the Guidelines for Accurate and Transparent Health Estimates Reporting (GATHER) statement (S1 Checklist)." When completing the checklist, please use section and paragraph numbers, rather than page numbers.**

**Response:** Not applicable.

***[FOR MEDIATION ANALYSES] We recommend that the study is reported according to the AGReMA statement (https://agrema-statement.org/#:~:text=AGReMA%20is%20an%20evidence%2D%20and,randomised%20trials%20and%20observational%20studies) and include the completed checklist as Supporting Information. Please add the following statement, or similar, to the Methods: "This study is reported as per the Guideline for Reporting Mediation Analyses (AGReMA) statement (S1 Checklist)." When completing the checklist, please use section and paragraph numbers, rather than page numbers.**

**Response:** Not applicable.

***For all observational studies, in the manuscript text, please indicate: (1) the specific hypotheses you intended to test, (2) the analytical methods by which you planned to test them, (3) the analyses you actually performed, and (4) when reported analyses differ from those that were planned, transparent explanations for differences that affect the reliability of the study's results. If a reported analysis was performed based on an interesting but unanticipated pattern in the data, please be clear that the analysis was data driven.**

**Response:** Thanks for your feedback. We have reviewed the entire manuscript and confirmed that it includes the required content.

***Please state in the Methods section whether the study had a prospective protocol or analysis plan. If a prospective analysis plan (from your funding proposal, IRB or other ethics committee submission, study protocol, or other planning document written before analyzing the data) was used in designing the study, please include the relevant document(s) with your revised manuscript as a Supporting Information file to be published alongside your study and cite it in the Methods section. A legend for this file should be included at the end of your manuscript. If no such document exists, please make sure that the Methods section transparently describes when analyses were planned, and when/why any data-driven changes to analyses took place. Changes in the analysis, including those made in response to peer review comments, should be identified as such in the Methods section of the paper, with rationale.**

**Response:** Thanks for your feedback. This is a retrospective study using the UK Biobank; thus, it is not applicable.

**MENDELIAN RANDOMIZATION STUDIES**

***Please ensure that the study is reported according to the STROBE-MR guideline (https://www.equator-network.org/reporting-guidelines/strobe/) and include the completed STROBE-MR checklist as Supporting Information. Please add the following statement, or similar, to the Methods: "This study is reported as per the Strengthening the Reporting of Observational Studies in Epidemiology (STROBE) guideline, specific for mendelian randomization (S1 Checklist)." When completing the checklist, please use section and paragraph numbers, rather than page numbers.**

**Response:** The necessary modifications have been made, and the corresponding content has been added.

**Methods (Page 13, Lines 251 and 252):** “*The MR study followed the STROBE-MR guidelines (S2 Checklist) [39].*”

***In the Introduction, please describe the exposure and the evidence for a potential causal relationship between exposure and outcome.**

**Response:** Thank you for this comment. The Introduction has been revised to clearly describe the exposure (physical frailty) and to strengthen the rationale for a potential causal relationship between physical frailty and suicide attempt. Specifically, we have expanded the discussion of biological and clinical mechanisms linking frailty to suicide-related outcomes, including reduced physiological reserve, impaired stress tolerance, functional decline, chronic inflammation, endocrine dysregulation, and metabolic disturbances, supported by existing empirical and theoretical evidence. This strengthened rationale provides the basis for the Mendelian randomization analyses, which were conducted as a complementary approach to support the observational cohort findings.

*** In the Methods, please explicitly state the 3 core instrumental variable assumptions for the main analysis (relevance, independence, and exclusion restriction), as well assumptions for any additional or sensitivity analysis.**

**Response:** Thank you for this comment. We have revised the Methods section to explicitly state the three core instrumental variable assumptions for the main Mendelian randomization (MR) analysis, namely relevance, independence, and exclusion restriction. We also clarified the assumptions underlying the additional and sensitivity analyses. Specifically, the relevance assumption was ensured by selecting genome-wide significant and independent SNPs; the independence and exclusion restriction assumptions were evaluated through multiple sensitivity analyses, including MR-Egger regression, weighted median, MR-PRESSO, heterogeneity tests, and leave-one-out analyses. Additional methodological details are provided in the Supplementary Methods (S1 Methods), in accordance with the STROBE-MR guidelines.

***In the Methods, please describe the MR estimator (e.g., 2-stage least squares, Wald ratio) and related statistics. Detail the included covariates and, in case of 2-sample MR, whether the same covariate set was used for adjustment in the 2 samples.**

**Response:** Thank you for this comment. The MR estimators (including the IVW estimator based on Wald ratio estimates), related statistics, and details on covariate adjustment are described in the Methods section of the main text and in the Supplementary Methods (S1 Methods). As this study employed a two-sample MR design, covariate adjustment was performed within the original GWAS analyses, and this information is also detailed in S1 Methods.

***If you are presenting an instrumental variable estimate, please compare this to the conventional observational estimate.**

**Response:** Thank you for this comment. We have compared the instrumental variable estimates from the MR analysis with the conventional observational estimates in the Results section. The MR results were directionally consistent with the conventional observational estimates. Both analyses showed that frailty is associated with an increased risk of suicidal attempt (SA), with genetically determined frailty showing an OR of 2.06 (95% CI: 1.21-3.52, *P* = 0.008), and the observational analysis showing an HR of 2.16 (95% CI: 1.78-2.61, *P* < 0.001).

***Report the associations between genetic variant and exposure and between genetic variant and outcome, preferably on an interpretable scale.**

**Response:** We have now reported the associations between each genetic variant and the exposure, as well as between each genetic variant and the outcome, on interpretable effect scales. The corresponding results have been added to the supplementary material (S5 Table).

***Report MR estimates of the relationship between exposure and outcome and the measures of uncertainty from the MR analysis, on an interpretable scale, such as odds ratio or relative risk per SD difference.**

**Response:** Thank you for this comment. The MR estimate of the association between exposure and outcome is reported in the Results section on an interpretable scale. Specifically, genetically determined frailty was associated with an increased risk of suicidal attempt, with an odds ratio of 2.06 (95% CI: 1.21-3.52, *P* = 0.008), as shown in Figure 1.

***If relevant, please consider translating estimates of relative risk into absolute risk for a meaningful time period.**

**Response:** Not applicable.

***Please consider including plots to visualize results (e.g., forest plot, scatterplot of associations between genetic variants and outcome vs between genetic variants and exposure).**

**Response:** Thank you for this suggestion. We have included visualizations such as forest plots to better illustrate our findings, as shown in Figure 1.
