# Supplementary material for: Physical frailty, genetic risk, mediating biomarkers, and risk of suicide attempt: A prospective cohort study
Source: PLoS Med. 2026 Apr 6;23(4):e1005045. doi: 10.1371/journal.pmed.1005045 (PMC13065332; doi:10.1371/journal.pmed.1005045)
Supplement: Attachment — Submitted filename: Point-by-Point-Response-R3.docx [file pmed.1005045.s007.docx]

**Can you please make sure to add all the tests used for p-value calculation in the main and supplementary figures and tables?**

**Response:** Thank you for your careful suggestion. We have now reviewed all main and supplementary figures and tables and added the corresponding statistical tests used for p-value calculation in each case, where applicable.

**Since you will be making amendments, I am pasting here a few more points that need to be addressed:**

**Line 51: in author summary: please slightly amend the sentence to make it clearer that the genetic risk refers to SA.**

**Response:** Thank you for your suggestion. We have revised the sentence to clarify that the genetic risk specifically refers to SA.

**Revised Line 51: “***Physical frailty may represent an important and previously under-recognized risk factor for SA, particularly among individuals with a higher genetic risk for SA.***”**

**Line 137: substitute "were" with "was"**

**Response:** Thank you for your careful review. We have revised “were” to “was” in line 137 as suggested.

**Revised Line 137:** “*The primary analytical framework was specified in advance and was conducted between March 2025 and May 2025.*”

**Line 377: please add: "although MR did not support a causal mediating role.” to clearly state the lack of confirmed causal association**

**Response:** Thank you for this helpful suggestion. We have revised the sentence to include “although MR did not support a causal mediating role,” thereby clearly indicating the lack of a confirmed causal association.

**Revised Line 377:** “*Furthermore, we found that approximately 14.2% of the frailty-SA association could be statistically explained by biomarkers related to liver function, metabolism, immune function, and inflammation, although MR did not support a causal mediating role.*”
